# Genome-Wide Identification of Regulatory RNAs in the Human Pathogen *Clostridium difficile*

**DOI:** 10.1371/journal.pgen.1003493

**Published:** 2013-05-09

**Authors:** Olga A. Soutourina, Marc Monot, Pierre Boudry, Laure Saujet, Christophe Pichon, Odile Sismeiro, Ekaterina Semenova, Konstantin Severinov, Chantal Le Bouguenec, Jean-Yves Coppée, Bruno Dupuy, Isabelle Martin-Verstraete

**Affiliations:** 1 Laboratoire Pathogenèse des Bactéries Anaérobies, Institut Pasteur, Paris, France; 2 Université Paris Diderot, Sorbonne Paris Cité, Cellule Pasteur, Paris, France; 3 Unité de Biologie des Bactéries Pathogènes à Gram Positif, Institut Pasteur, Paris, France; 4 Plate-forme Transcriptomes et Epigénome, Departement Génomes et Génétique, Institut Pasteur, Paris, France; 5 Waksman Institute, Piscataway, New Jersey, United States of America; 6 Institutes of Molecular Genetics and Gene Biology, Russian Academy of Sciences, Moscow, Russia; 7 Department of Molecular Biology and Biochemistry, Rutgers, The State University, Piscataway, New Jersey, United States of America; Universidad de Sevilla, Spain

## Abstract

*Clostridium difficile* is an emergent pathogen, and the most common cause of nosocomial diarrhea. In an effort to understand the role of small noncoding RNAs (sRNAs) in *C. difficile* physiology and pathogenesis, we used an *in silico* approach to identify 511 sRNA candidates in both intergenic and coding regions. In parallel, RNA–seq and differential 5′-end RNA–seq were used for global identification of *C. difficile* sRNAs and their transcriptional start sites at three different growth conditions (exponential growth phase, stationary phase, and starvation). This global experimental approach identified 251 putative regulatory sRNAs including 94 potential *trans* riboregulators located in intergenic regions, 91 *cis*-antisense RNAs, and 66 riboswitches. Expression of 35 sRNAs was confirmed by gene-specific experimental approaches. Some sRNAs, including an antisense RNA that may be involved in control of *C. difficile* autolytic activity, showed growth phase-dependent expression profiles. Expression of each of 16 predicted c-di-GMP-responsive riboswitches was observed, and experimental evidence for their regulatory role in coordinated control of motility and biofilm formation was obtained. Finally, we detected abundant sRNAs encoded by multiple *C. difficile* CRISPR loci. These RNAs may be important for *C. difficile* survival in bacteriophage-rich gut communities. Altogether, this first experimental genome-wide identification of *C. difficile* sRNAs provides a firm basis for future RNome characterization and identification of molecular mechanisms of sRNA–based regulation of gene expression in this emergent enteropathogen.

## Author Summary

The emergent human pathogen *Clostridium difficile* is a major cause of nosocomial diarrhea associated with antibiotic therapy. During the last few years, severe forms of *C. difficile* infections became more frequent due to the emergence of hypervirulent isolates. Despite intensive studies, many questions regarding the mechanisms controlling *C. difficile* virulence remain unanswered. We hypothesized that *C. difficile*, a member of an ancient group of bacteria, might widely use ancestral RNA–based mechanisms to control its gene expression for better adaptation to host conditions. Indeed, using next-generation sequencing technology, we identified a great number and a large diversity of potential RNA regulators in this pathogen. We obtained experimental evidence for regulatory roles of a particular class of regulatory RNAs responding to c-di-GMP, a universal bacterial signaling molecule regulating motility, biofilm formation, and virulence. We also detected abundant small RNA products of recently discovered adaptive prokaryotic immunity CRISPR-Cas systems that might be important for *C. difficile* survival in gut communities. Our findings suggest that small RNA molecules may play a major role in regulatory processes during *C. difficile* infection cycle and as such are promising targets of new therapeutic strategies.

## Introduction

In recent years, the importance of regulatory mechanisms based on the action of RNA molecules became widely appreciated. In bacteria, regulatory RNAs play a critical role in adaptive responses and in various metabolic, physiological, and pathogenic processes. In particular, small noncoding RNAs (sRNAs) have been recently identified in many pathogenic bacteria [1]–[3]. Such sRNAs rely on a variety of mechanisms to control their targets, including *i*) direct binding to low-molecular weight effector molecules (riboswitches); *ii*) binding to proteins; *iii*) interaction with double-stranded DNA or RNA (CRISPR, clustered regularly interspaced short palindromic repeats RNAs); and *iv*) RNA/RNA duplex formation with mRNA targets [4]. Riboswitches are *cis*–acting elements commonly involved in the control of vitamin, amino acid, and nucleotide base biosynthesis gene expression in bacteria. Upon interaction with ligands (usually the products of biosynthetic operons they control), riboswitches undergo a conformational change leading to positive or negative effects on transcription termination or translation [5]. Protein-binding sRNAs often directly antagonize the function of their targets [6], [7]. For example, the widely distributed 6S RNA acts as a promoter decoy for RNA polymerase holoenzyme containing major sigma 70 factor by mimicking an open promoter complex and globally regulating transcription during adaptation to stationary phase of growth [7]. CRISPR RNAs contain short regions of complementarity to bacteriophage and plasmid sequences. In complex with Cas proteins, CRISPR RNAs interfere with bacteriophage infection and plasmid invasion by recognizing foreign DNA and targeting it for destruction [8]. The largest and most extensively studied set of sRNA regulators acts by modulating the translation and/or stability of specific mRNA targets in response to changes in the environment [9]. These regulatory RNAs can be divided into *cis*-encoded RNAs, which are transcribed from a DNA strand opposite to the one from which mRNA to be regulated is transcribed and are thus fully complementary to their targets [10], [11], and *trans*-encoded RNAs, which are transcribed from separate loci and are only partially complementary to target mRNAs [9]. In many cases, the RNA-chaperone Hfq protein is required for *trans*-encoded sRNA-mediated control [12].

With the exception of recent identification of cyclic-di-guanosyl-5′monophosphate (c-di-GMP) riboswitches [13]–[15], no experimental data on regulatory RNAs in *Clostridium difficile* has been reported. However, recent genome-wide *in silico* searches for sRNAs within intergenic regions (IGRs) [16]–[18] suggested that riboregulators may be abundant in clostridia including *C. difficile* and could be involved in the control of metabolism and virulence. In *Clostridium perfringens*, four sRNAs controlling expression of toxin-encoding genes were experimentally identified [19]–[21], including VirX, which was the first clostridial regulatory RNA discovered by Ohtani and Shimizu [20], [21]. In *Clostridium acetobutylicum*, we have recently described a novel mechanism of control of a sulfur metabolic operon by an antisense RNA [22].

*C. difficile* is an anaerobic spore-forming bacterium that is a major cause of nosocomial infections associated with antibiotic therapy. This enteropathogen can lead to antibiotic-associated diarrhea and pseudomembranous colitis, a potentially lethal disease. Transmission of *C. difficile* is mediated by contamination of the gut by its spores. The disruption of colonic microflora by antimicrobial therapy precipitates colonization of the intestinal tract by *C. difficile* and ultimately leads to infection [23]. After spore germination, vegetative forms multiply and major virulence factors, the two large toxins, TcdA and TcdB, are produced causing alterations in the actin cytoskeleton of intestinal epithelial cells [24]. Many aspects of *C. difficile* infection cycle, including the identification of additional virulence and colonization factors and the determination of molecular mechanisms controlling their production in response to environmental signals, still remain poorly understood [25], [26]. We hypothesize that sRNAs may contribute to these important processes.

To systematically search for regulatory RNAs in *C. difficile*, we here used a robust *in silico* approach that has been successfully applied for the identification of sRNAs in *Escherichia coli* and *Streptococcus agalactiae* [27]. This led to the identification of a large number of sRNA candidates in both intergenic and coding regions of the *C. difficile* genome. RNA-seq and differential 5′-end RNA-seq approaches were then used to validate these predictions and to globally identify *C. difficile* sRNAs and their transcriptional start sites. In this way, the expression of numerous sRNAs located in IGRs and representing potential *trans* riboregulators, *cis*-antisense RNAs, riboswitches, and CRISPR RNAs was detected. The expression of several sRNAs was independently confirmed by gene-specific approaches, and potential involvement of some of them in growth-stage specific control in *C. difficile* was revealed. This study thus constitutes a basis for the future detailed functional characterization of RNA-based regulatory mechanisms in *C. difficile*.

## Results/Discussion

### Identification of putative sRNA candidates by *in silico* approach in *C. difficile* genome

**Figure 1:**
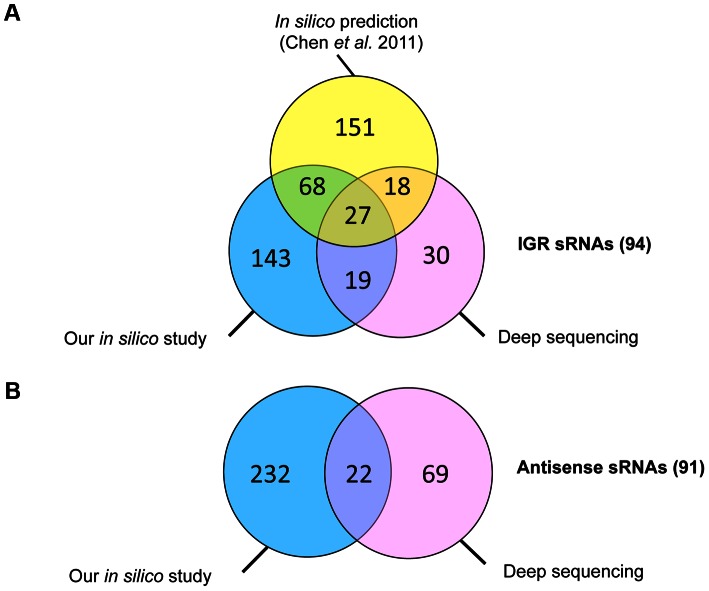
Comparative analysis of sRNAs identified in this study by comparative genomics and bioinformatics and by deep sequencing with those predicted in IGR in previously published study. Venn diagram representations of the number of sRNAs in IGR (A) and antisense RNAs to CDSs (B) identified by our bioinformatics analysis (in blue), predicted by previously published *in silico* analysis (in yellow) [16] and experimentally observed in our deep sequencing data (in pink).

Predicted *C. difficile* Rho-independent terminator (RIT) sites, which often terminate sRNA gene transcription, were used as a starting point for an *in silico* sRNA search according to a method previously described for *S. agalactiae* [27]. In the 4,290,252-bp genome of *C. difficile* strain 630, 2644 putative RIT sites were found. To identify the transcription termination signals associated with sRNA genes, we filtered out the RIT sites associated with protein coding genes (*i.e.*, located within −25 to +60 nt of stop codons of preceding open reading frames (ORFs)) as well as the RIT sites with calculated free Gibbs energy ΔG^0^_37_>−4 kcal/mol. sRNA gene candidates located antisense to coding sequences (CDSs) were filtered out if ΔG°_37_ of their RIT sites was more than −8 kcal/mol. The conservation of remaining sequences and the presence of covariations in predicted RNA secondary structure elements of RIT was next analysed using comparison with available clostridial sequences in GenBank database. sRNA candidates were then scored on the basis of their RIT score, which was weighted by the number of covariation pairs found. Threshold ΔG^0^_37_ values of −4 kcal/mol or −8 kcal/mol and a requirement for at least two covariations, including one in the RIT stem left 511 sRNA candidates (257 sRNAs in IGRs and 254 antisense RNAs) that were classified according to their position relative to adjacent CDSs. These predicted sRNAs are summarized in Table 1 and a detailed list of sRNA candidates (named from “SQ1” to “SQ2610”) is given in Table S1. A recent study searching for potential sRNAs in IGRs in clostridial genomes that combined comparative genomics with promoter and RIT predictions [16] identified 264 sRNA candidates in the *C. difficile* 630 strain. The comparison of our data with the previous study revealed 95 IGRs containing common potential sRNAs (Figure 1A). The strategy of Chen *et al* [16] does not detect potential regulatory RNAs located antisense to known CDSs. 147 such sRNAs were predicted by our method alongside with 107 additional antisense RNAs overlapping 5′ or 3′ untranslated regions of *C. difficile* genes (Table 1, Figure 1B).

**Table 1:** Summary of sRNA candidates in *C. difficile*.

Method of detection	Total	IGR	Antisense CDS	Antisense 3′UTR	Antisense 5′UTR
***In silico*** ** prediction**	511	257	147	79	28
Confirmation by RT-PCR	28	19	4	5	0
Confirmation by Northern blot	9	8	1	0	0
Confirmation by deep sequencing (RNA-seq and/or TSS mapping)	68	46	10	9	3
**Deep sequencing**	**Total**	**IGR**	***Cis*** **-antisense sRNAs**	**Riboswitches**	
Predicted *in silico*	126	46	22	58	
New	125	48	69	8	
Total regulatory RNAs	251	94	91	66	

sRNA localization: IGR, intergenic region; antisense CDS, sRNA antisense to coding sequence, antisense 3′UTR, antisense overlapping the 3′untranslated region of CDS; antisense 5′UTR, antisense overlapping the 5′untranslated region of CDS.

### Experimental detection of sRNAs by RNA–seq and 5′-end RNA–seq

High-throughput sequencing of cDNA allows strand-specific identification of transcripts with single-nucleotide resolution. For several bacterial species, such deep sequencing technologies revealed an unexpected transcriptome complexity and identified a large number of novel sRNAs [28], [29]. For genome-wide detection of expressed sRNAs in *C. difficile*, we combined two independent sequencing approaches - whole-transcript cDNA sequencing (RNA-seq) and differential 5′-end sequencing (5′-end RNA-seq allowing global identification of transcriptional start sites (TSS)). The RNA-seq analysis was performed with RNAs extracted from *C. difficile* 630Δ*erm* strain after 6 h of growth (late exponential phase). For 5′-end sequencing, we mixed three different RNA samples extracted from cells harvested in exponential growth phase (4 h of growth), at the onset of stationary phase (10 h of growth), and under nutrient starvation conditions. For RNA-seq, a non-orientated whole-transcript library was generated from late exponential phase RNA sample depleted for sRNAs. For 5′-end RNA-seq, two strand-specific cDNA libraries were generated from mixed RNA sample depleted for rRNAs. The first library was constructed from RNA sample treated with Tobacco Acid Pyrophosphatase (TAP+) allowing inclusion of reads associated with the TSS. A second library was constructed from the same RNA sample without TAP treatment (TAP−). The libraries were subjected to Illumina sequencing yielding a total of 37 million of whole-transcript reads (RNA-seq) and a total of 75.5 million and 38 million of 5′-end reads for TAP+ and TAP− libraries (5′-end RNA-seq), respectively. After removal of reads mapping to rRNA genes, about 6 to 10% of total reads were mapped to the genome of *C. difficile* strain 630 (for details see Table S2). For TSS identification, the sequencing data were compared between TAP+ and TAP− libraries. This approach allowed us to identify 251 TSSs upstream of potential sRNA genes. 66 TSSs are located upstream of riboswitches and 185 upstream of other sRNAs (Table 1). Within the latter group, 94 sRNAs are encoded within IGRs and may therefore represent *trans* RNA regulators, while 91 sRNAs are transcribed in an antisense direction to coding regions and may therefore represent *cis* RNA regulators (Table 1, Table S3). Potential riboswitches were first defined as sRNAs having a characteristic RNA-seq profile and TSS located at a significant distance from a downstream coding gene, usually with a metabolic function. The riboswitch nature of most of these RNAs was further confirmed by Rfam search (see below).

Some of the 185 non-riboswitch sRNAs identified by deep sequencing were also predicted by the *in silico* analysis (Figure 1). The incomplete overlap between experimental sRNA detection and bioinformatics predictions has been already noted in other studies [30], [31]. This may be due to a requirement for a particular growth condition for expression of some of *in silico* predicted sRNAs, the restriction of bioinformatics predictions to sRNAs conserved in closely related species, the RIT criteria eliminating sRNAs derived from 3′-ends of coding genes, or the presence of false positive candidates among *in silico* predictions. We preserved the SQ names for *in silico*-predicted sRNA candidates (Table S1) and named additional sRNA candidates identified by deep sequencing according to their position in the genome, from CD630_n00010 to CD630_n01130 in agreement with recently published *C. difficile* gene nomenclature [32] (Table S3).

### Experimental validation of sRNA candidates

We confirmed several selected sRNAs predicted *in silico* and/or identified by deep sequencing using gene-specific experimental approaches: reverse-transcription-PCR (RT-PCR) and Northern blot analysis. Expression of selected sRNAs was monitored under different growth conditions of *C. difficile* 630Δ*erm* cultures to reveal the cases of growth phase-dependent expression. To determine if selected sRNAs are also present in another *C. difficile* strain, we extracted RNA from a PCR-ribotype 027 hypervirulent strain (R20291) during late exponential growth phase and also subjected it to analysis.

**Figure 2:**
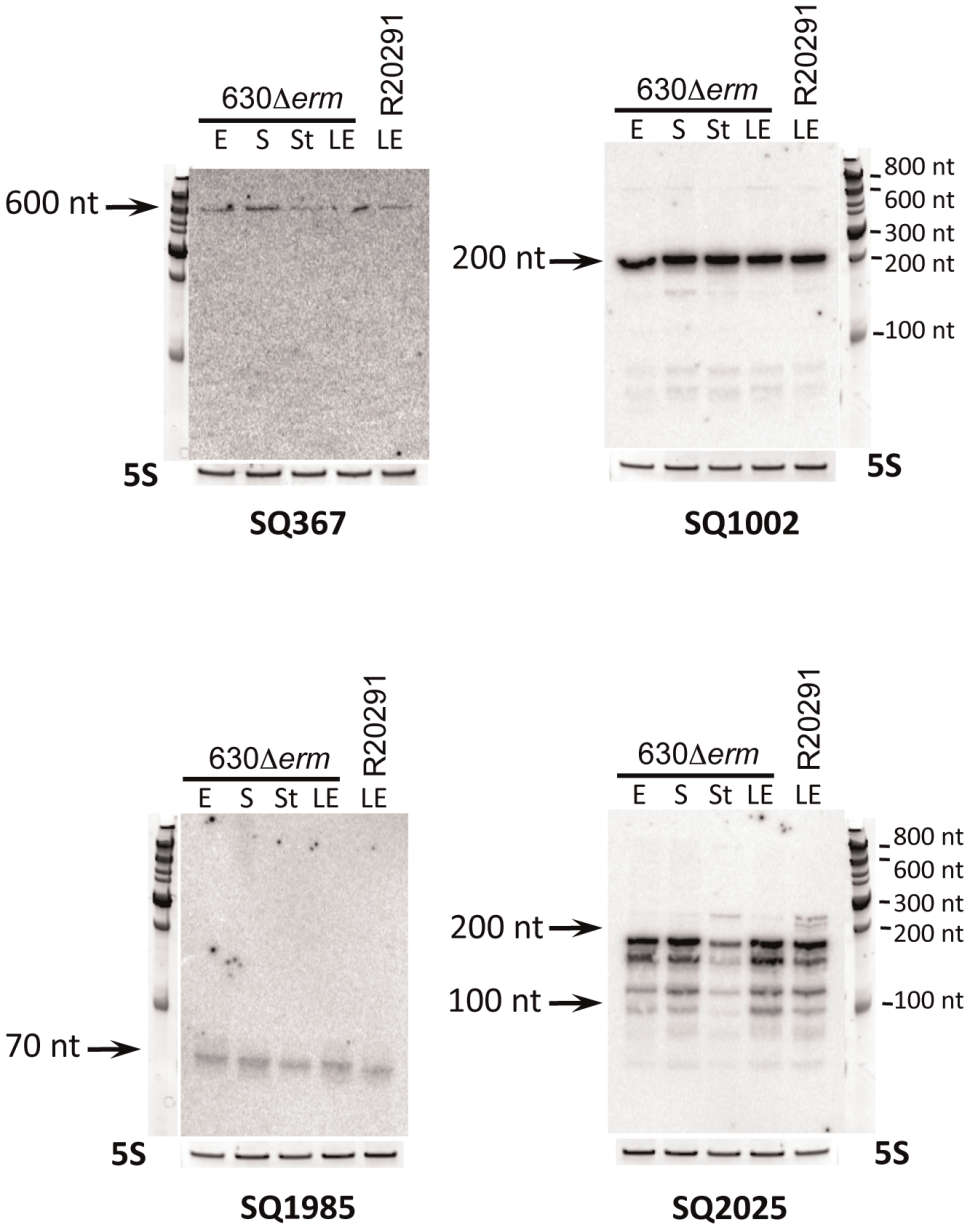
Experimental validation of *in silico* identified sRNAs. Northern blot was performed for detection of selected sRNAs: SQ367, SQ1002, SQ1985 and SQ2025. RNA samples were extracted from 630Δ*erm* strain grown at exponential phase (E, 4 h of growth), late exponential phase (LE, 6 h of growth), entry to stationary phase (S, 10 h of growth) or under nutriment starvation conditions (St) and from R20291 strain grown at late exponential phase (LE). 5S RNA at the bottom serves as loading control. The arrows show the detected transcripts with their size estimated by comparison with RNA molecular weight standards.

RT-PCR analysis revealed that 28 out of 30 *in silico* predicted sRNA candidates were transcribed in strain 630Δ*erm* during the exponential growth phase and/or at the onset of stationary phase. A representative example of experimental validation of five sRNA candidates expression by RT-PCR is presented in Figure S1. Expression of 9 and 7 out of 13 *in silico* predicted sRNA candidates in strain 630Δ*erm* and R20291, respectively, was detected by Northern blotting. Figure 2 shows a representative result for four sRNAs predicted *in silico* and detected by RNA-seq in both strains. As can be seen, Northern blot analysis with probes specific for SQ1002 candidate sRNA revealed an abundant ∼200-nt transcript, which agrees well with the 236-nt *in silico* predicted length and the 151-nt length deduced from RNA-seq data. For SQ1985 (predicted length of 233-nt), a much shorter, ∼70-nt low-abundance transcript was detected in accordance with the 84-nt length revealed from RNA-seq data. The discrepancy in lengths may be explained by *in silico* prediction criteria (maximum size limit fixed at 250 nt) as well as the fact that predictions did not take in account the positions of promoters. No growth phase dependence was observed for the expression of sRNA candidates tested. However, examples of growth phase-dependent regulation of *C. difficile* sRNAs will be discussed later in the text.

**Figure 3:**
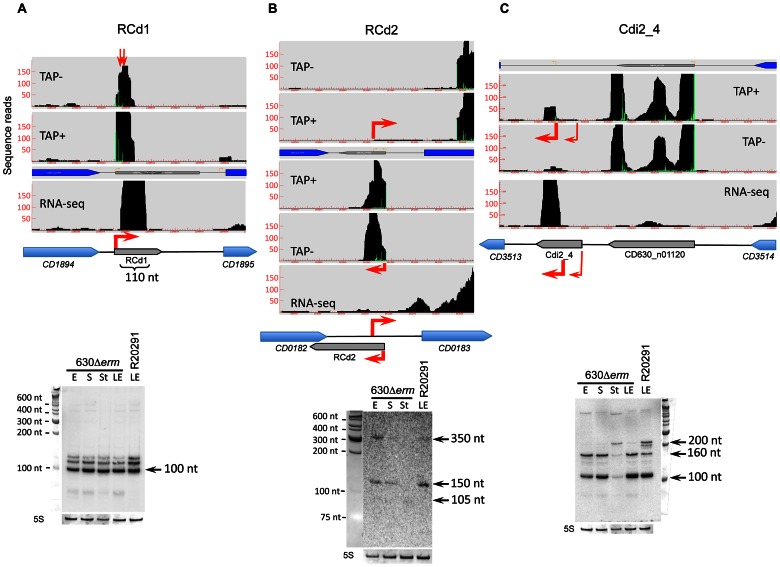
Detection of selected sRNAs by RNA–seq, 5′-end RNA–seq, and Northern blot. A representative example of 5′-end RNA-seq (TAP-/TAP+ profile comparison), RNA-seq and Northern blot detection of sRNA located in IGR (A); *cis*-antisense RNA (B) and riboswitch (C). On a RNA-seq and 5′-end RNA-seq sequence read mapping visualization, coding sequences are indicated by blue arrows and new sRNA candidates identified in this study are indicated by grey arrows. The 5′-end RNA-seq data for both strands are presented in upper and lower parts of B panel. The TSS identified by 5′-end RNA-seq are indicated by red broken arrows in accordance with the positions of 5′-transcript ends shown by vertical green lines on the sequence read graphs corresponding either to TSS (broken arrows) or to processing sites (vertical arrows). The TSS corresponds to position with significantly greater number of reads in TAP+ sample, potential cleavage site corresponds to position with large number of reads in both TAP− and TAP+ samples. 5′-end RNA-seq data show 51-bp reads matching to the 5′-transcript ends, while RNA-seq data show reads covering whole transcript. For Northern blot analysis, RNA samples correspond to those indicated for Figure 2. 5S RNA at the bottom serves as a loading control. The arrows show the detected transcripts with their estimated size.

**Figure 4:**
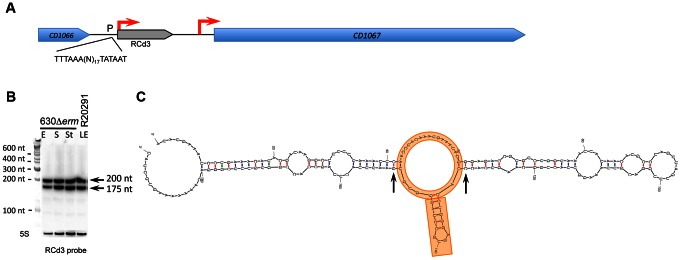
Detection and secondary structure prediction of abundant 6S RNA. In RCd3 (6S RNA) genomic region (A) coding sequences are indicated by blue arrows and the 6S RNA is indicated by a grey arrow. For Northern blot analysis (B) RNA samples correspond to those indicated for Figure 2. 5S RNA at the bottom serves as loading control. The arrows on the right show the detected transcripts with their estimated size. (C) The RNA secondary structure prediction was performed by Mfold software. Central asymmetric bubble mimicking open promoter structure and conserved short stem-loop structure on one side of this bubble are underlined in orange. Two generally conserved G-C base pairs surrounding this structure are indicated by arrows.

**Figure 5:**
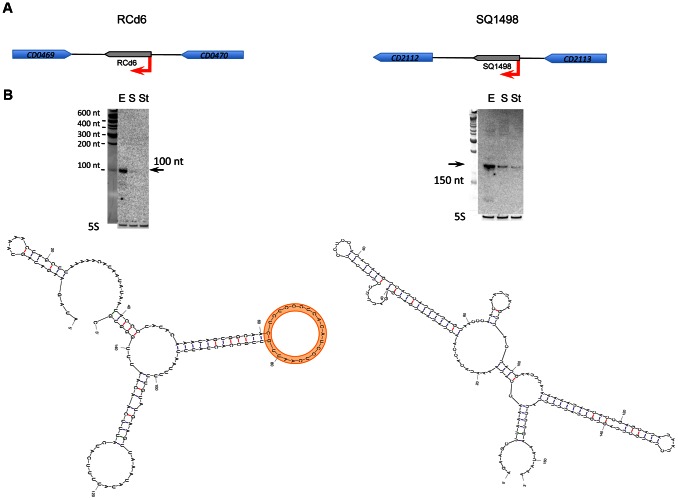
Expression analysis and secondary structure prediction of growth phase-regulated sRNAs. The results are given at the left for RCd6 sRNA and at the right for SQ1498. In corresponding genomic region (A) coding sequences are indicated by blue arrows and new sRNA candidates identified in this study are indicated by grey arrows. For Northern blot analysis (B) RNA samples correspond to those indicated for Figure 2 for 630Δ*erm* strain. 5S RNA at the bottom serves as loading control. The arrows show the detected transcripts with their estimated size. The RNA secondary structure prediction was performed by Mfold software. The loop region of predicted target interaction for RCd6 is highlighted in orange.

**Figure 6:**
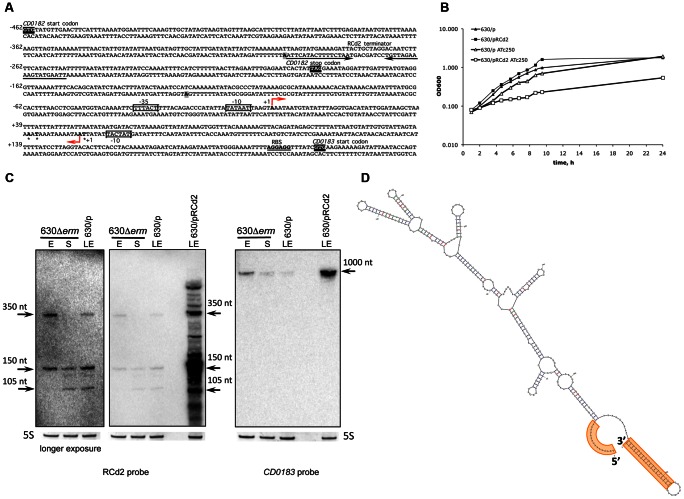
Effect of RCd2 RNA overexpression on target gene control. (A) Sequence of RCd2 RNA within *CD0182* and *CD0183* IGR. The TSS “+1” for antisense RNA and *CD0183* mRNA identified by 5′-end RNA-seq are indicated by broken arrows. The 5′- and 3′-ends of RCd2 RNA identified by 5′/3′RACE are shown in bold and are indicated by stars and in grey boxes, respectively. The −10 and −35 regions are boxed. The transcriptional terminator for RCd2 is indicated by convergent arrows. The *CD0182* and *CD0183* start codons and the *CD0182* stop codon are shown in black. The ribosome binding site (RBS) of *CD0183* is inderlined. The numbers indicate positions relative to the *CD0183* TSS. (B) Growth of 630/p strain (triangles) and 630/pRCd2 strain (squares) in TY medium at 37°C in the presence (open symbols) or absence (closed symbols) of 250 ng/mL ATc. (C) Effect of RCd2 overexpression on the *CD0183* transcript abundance. For Northern blot analysis, RNA samples were extracted from 630Δ*erm* strain during exponential growth phase (E, 4 h of growth), at the onset of stationary phase (S, 10 h of growth) and from 630/p control strain or from 630/pRCd2 strain overexpressing RCd2 grown at late exponential growth phase (LE) in the presence of 250 ng/mL ATc (630/p, 630/pRCd2). Detection with RCd2-specific probe is shown on the left and with *CD0183*-specific probe on the right. 5S RNA at the bottom serves as loading control. Longer exposure time was required for better detection of RCd2 transcripts in 630Δ*erm* and 630/p strains. The RNA secondary structure prediction (D) was performed by Mfold software. The terminator at the 3′-end and loop region at the 5′-end overlapping 5′-part of *CD0183* mRNA are highlighted in orange.

**Figure 7:**
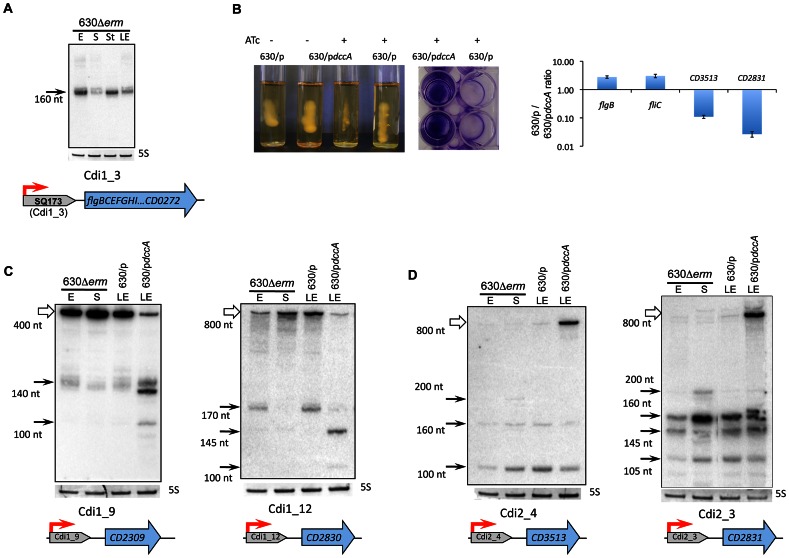
Functional analysis of c-di-GMP riboswitches in *C. difficile*. (A) Northern blot detection of SQ173 located upstream of the *flgB* flagella operon. Coding sequences are indicated by blue arrows and SQ173 is indicated by a grey arrow. For Northern blot analysis, RNA samples correspond to those indicated for Figure 2. 5S RNA at the bottom serves as loading control. The arrow on the left shows the detected transcripts with their estimated size. (B) Effect of overexpression of *CD1420* (*dccA*) encoding diguanylate cyclase on motility and biofilm formation. A representative result of motility and biofilm formation assay of strains 630/p*dccA* (pRPF185-*CD1420*) and 630/p (pRPF185) performed in the absence or in the presence of ATc (500 ng/mL) inducing the expression of the *CD1420* gene. qRT-PCR data show fold change for 630/p*dccA*/630/p ratio for the *flgB* gene positively regulated by Cdi1_3 riboswitch, flagellin-encoding *fliC* gene and *CD3513, CD2831* genes negatively regulated by c-di-GMP riboswitches of type II. Northern blot analysis of c-di-GMP-I (C) and c-di-GMP-II (D) riboswitches. RNA samples correspond to those indicated for Figure 6, 630/p and 630/p*dccA* strain RNA samples were extracted at late exponential growth phase (LE) in the presence of 500 ng/mL ATc. White arrows correspond to target gene full-length transcripts and black arrows to premature terminated transcripts and processed forms (C) or stable spliced forms (D).

**Figure 8:**
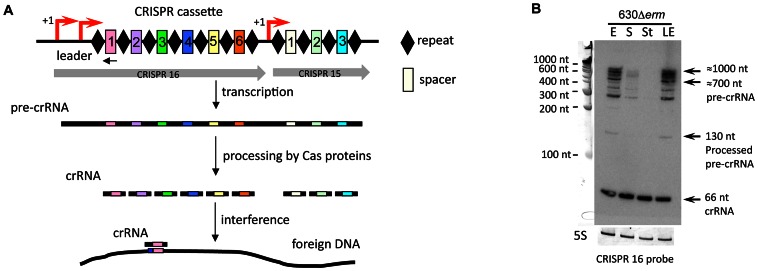
Expression of CRISPR 16 cassette. The structure of CRISPR 16 and CRISPR 15 cassettes on *C. difficile* strain 630 chromosomal region is schematically shown (A) with spacers indicated as rectangles numbered according to the transcription order and repeats indicated as diamonds. Regions corresponding to CRISPR 15 and CRISPR 16 cassettes are indicated by grey arrows. The TSS with “leader” region is indicated by red arrow. The position of the oligonucleotide complementary to the first spacer sequence of CRISPR 16 locus used as probe for Northern blot is indicated by black arrow. CRISPR cassette is transcribed into a single RNA transcript (pre-crRNA), which is cleaved by the Cas proteins to generate small CRISPR RNAs (crRNAs), each containing one spacer. Spacers match foreign DNA and this recognition lead to interference process resulting in bacterial cell protection. For Northern blot analysis (B), RNA samples were extracted from 630Δ*erm* strain grown at exponential phase (E), late exponential phase (LE), entry to stationary phase (S) or under nutriment starvation conditions (St). 5S RNA serves as loading control. Arrows indicate full-length pre-crRNA transcripts, incompletely processed crRNAs and crRNAs with their estimated size.

Among the 185 *C. difficile* 630Δ*erm* sRNAs identified by deep sequencing, 35 out of 40 candidates assayed were detected by Northern blotting of 630Δ*erm* strain RNA samples, while 23 were detected in RNA prepared from the R20291 strain samples. Transcript lengths agreed well with the sizes deduced from RNA-seq approach (Figure 2, Figure 3, Figure 4, Figure 5, Figure 6, Figure 7, Figure 8; Figures S2, S3, S4, S5) and were confirmed by independent 5′/3′RACE analysis for 4 selected RNAs (Table S4). 5′RACE experiments unambiguously identified TSSs for eight potential sRNAs and were in complete agreement with the TSSs identified by 5′-end RNA-seq (Table S4).

We identified TSS of antisense transcripts in genes required for flagella synthesis, encoding prophage proteins, transcriptional regulators, and membrane proteins (Table S3). Generally, the expression of *cis*-antisense RNAs was more difficult to detect than that of sRNAs located in IGRs. Figure 3 shows representative examples of each regulatory RNA type detected in 630Δ*erm* and R20291 strains: an IGR (CD630_n00660, named RCd1), a *cis*-antisense (CD630_n00030, named RCd2), and a riboswitch (Cdi2_4). The RCd1 sRNA is transcribed from IGR between *CD1894* and *CD1895* genes encoding conserved hypothetical proteins. An abundant transcript of about 100-nt was stably detected under all tested conditions by Northern blotting (Figure 3A). Interestingly, the presence of two additional less abundant longer transcripts was consistent with the detection of possible processing sites downstream from the TSS in 5′-end RNA-seq analysis (indicated by arrows in Figure 3A). The RCd2 RNA is transcribed in antisense orientation to the 3′UTR of *CD0182* encoding a conserved hypothetical protein. As can be seen from Figure 3B, two major transcripts of about 150 and 350 nt in length were detected. The type II c-di-GMP riboswitch (Cdi2_4) is located upstream of a pilin encoding gene *CD3513* (Figure 3C) [14]. A major transcript of about 100 nt was detected in Northern blot together with two additional less abundant 160-nt and 200-nt transcripts (Figure 3C). The role of RCd2 and Cdi2_4 sRNAs is discussed later in the text.

Overall, the results of validation of *in silico* prediction and deep sequencing indicate that both approaches are valuable and robust tools for identification of sRNAs in *C. difficile*. Since each method has its own advantages and disadvantages, the combination of both approaches appears to be optimal for the systematic detection of sRNAs. However, focusing only on predicted sRNAs confirmed by RNA-seq is too restrictive since a number of potentially interesting candidates was identified by deep sequencing alone. The presence of multiple validated sRNAs from different riboregulator families demonstrates the diversity and wide spread use of RNA-mediated control in *C. difficile*.

### Features of sRNAs

We analysed the nucleotide sequences upstream of experimentally identified sRNA TSSs to search for promoters. In most cases, we detected sequences matching the −10 or −10 and −35 promoter consensus elements recognized by the RNA polymerase sigma A holoenzyme (Table 2). However, since promoter elements recognized by the 22 other sigma factors encoded in the *C. difficile* genome are still poorly defined, transcription of sRNAs by other holoenzymes is possible and deserves further investigation.

**Table 2:** Features of sRNA candidates identified by RNA–seq.

	IGR sRNAs (94)	*Cis*-antisense sRNAs (91)	Riboswitches*** (66)
**Predicted promoters**	33 sigma A promoters	63 sigma A promoters	13 sigma A promoters (c-di-GMP I, c-di-GMP II)
**Presence of putative CDSs**	48 (9 RBS, 9 ? RBS; 11 conserved ORFs*)	22 (2 ? RBS; 2 conserved ORFs*)	3
**Function prediction by Rfam database**	1 6S; 1 tRNA-Sec; 12 CRISPR-DR14; 1 tmRNA		20 T-box [36]; 5 SAM [37]; 3 FMN; 4 Lysine [38]; 1 TPP; 2 Purine; 2 Glycine; 1 Cobalamin; 12 c-di-GMP I (GEMM RNA motif) [15]; 4 c-di-GMP II [13], [14]; 2 *ykkC-yxkD*-like leader
**Conservation among 14 ** ***C. difficile*** ** strains**	69	60**	32****

“RBS” Shine Dalgarno sequence for ribosome binding site, “? RBS”, questionable ribosome binding site.

* Conservation of ORFs among 14 sequenced *C. difficile* strains. RNA sequences were analysed for matches in a collection of RNA families Rfam database (http://rfam.sanger.ac.uk/).

** The analysis for *cis*-antisense RNA was biased by the conservation of overlapping coding sequences.

*** Riboswitches responding to S-adenosyl methionine (SAM), flavin mononucleotide (FMN), thiamine pyrophosphate (TPP).

**** CD37 genome without gene annotation was excluded from this analysis.

Since small proteins of less than 50 amino acids have been largely disregarded in genome annotations [33], one cannot exclude that some sRNAs identified in this study encode short proteins or peptides. We therefore searched for ORFs within our sRNA sequences collection. 73 sRNAs encoding at least one putative ORF of more than 15 amino acids were identified. Since some sRNAs contained multiple ORFs, the total number of putative sRNA-encoded ORFs is 106. However, only 9 sRNAs contained a predicted start codon connected to a reasonably located recognizable putative ribosome binding-site (RBS), while 11 sRNAs contained only a questionable RBS (Table 2). 13 of these 20 ORFs were conserved in all 14 sequenced *C. difficile* strains (Table 2). Comparative genomic analysis showed that only 7 sRNA-encoded sequences were similar to fragments of known proteins. The similarity was due to an overlap of these sRNAs with 3′ends of adjacent protein-encoding genes. Therefore, we conclude that most of identified *C. difficile* sRNAs are unlikely to encode small proteins. Nevertheless, definitive exclusion of peptide encoding by corresponding DNA regions needs further experimental verification.

We next used the Rfam database (a collection of noncoding RNA families) to search for *C. difficile* sRNAs similar to known sRNAs [34]. The summary of this analysis is given in Table 2. For the majority of IGR and *cis*-antisense *C. difficile* sRNAs, no functional class can be assigned suggesting that they may represent novel sRNA regulators. As expected for relatively understudied Gram-positive bacterium *C. difficile*, only few identified sRNAs corresponded to known functional classes. Among them, CD630_n00410 (RCd3) sRNA matches the housekeeping 6S RNA and CD630_n00840 corresponds to selenocysteine tRNA. A total of 24 annotated tRNAs were also detected by deep sequencing. 12 other highly expressed sRNAs originated from *C. difficile* CRISPR cassettes (see below). Most of identified regulatory RNAs for which a functional class can be assigned correspond to riboswitches. Thus, in addition to 16 c-di-GMP-dependent riboswitches carrying either GEMM RNA or c-di-GMP II motif [14], [15], we could predict a function for 39 of remaining riboswitches (Table 2, Table S3). T-box antitermination is one of the main mechanisms controlling amino acid biosynthesis in Gram-positive bacteria [35], [36]. Accordingly, 20 *C. difficile* RNA regulatory elements detected in our RNA-seq analysis corresponded to T-box riboswitches. We also detected that all of the 5 predicted SAM-riboswitches and all of the 4 predicted L-box riboswitches are located upstream of genes involved in methionine biosynthesis or uptake [37] and in lysine biosynthesis and transport [38], respectively. In addition, we detected several known ribozymes including 8 group I introns upstream of transposase genes and a ribozyme corresponding to RNase P of type A (Table S3).

In accordance with Northern blot results general BLAST analysis revealed that about 80% of all *C. difficile* 630Δ*erm* sRNAs detected by deep sequencing were also present in the hypervirulent R20291 strain, which has about 80% genome sequence similarity with the 630 strain [39]. To better explore the conservation of sRNAs within the *C. difficile* species, we used BLAST to analyse the distribution of sRNAs among available *C. difficile* genomes. The results revealed that most sRNAs are conserved, with 69 IGR sRNAs being present in all 14 sequenced *C. difficile* strains (Table 2, Table S5). Moreover, 32 conserved riboswitches were identified upstream of orthologous genes in all 13 annotated *C. difficile* genomes. Finally, the sequences homologous to 60 *cis*-antisense RNAs were also found in all *C. difficile* strains. However, for *cis*-antisense RNAs this analysis was partially biased by the overlap with conserved coding sequences. Nevertheless, the high degree of conservation of the sRNAs identified among the *C. difficile* strains emphasizes their functional importance in this bacterium.

We next extended the analysis of sRNA conservation to other clostridia. Less stringent BLAST searches (alignment restriction length lowered from 80 to 50% of sRNA length) revealed that only few of *C. difficile* sRNA genes are present in at least one other clostridial species (Table S5). Two IGR sRNAs, CD630_n01090 and CD630_n00840, are found in *C. perfringens* and *C. sticklandii*. Two antisense RNAs, CD630_n00090 and SQ771, are found in *C. beijerinckii*, and in *C. botulinum, C. ljungdahlii, C. perfringens*, and *C. tetani*, respectively, but are located within conserved coding sequences. Our apparent inability to detect homologues of most sRNAs in clostridia is somewhat contradictory to conclusions from previously published *in silico* analysis [16], which showed that about half of predicted clostridial sRNAs in IGRs are conserved among most clostridial species. However, in accordance with previous studies, the conservation of some riboswitches was revealed. Only few sRNA candidates were conserved outside Clostridia (Table S5). Obviously, further work will be necessary to experimentally detect and catalogue regulatory RNAs in other clostridial species. The regulatory RNAs revealed in this work for the most part are unique to *C. difficile* and could be associated with specific physiopathology of this bacterium.

### Examples of RNA potentially involved in regulatory functions

Below, we present a more detailed analysis of several *C. difficile* sRNAs belonging to different functional classes of riboregulators including IGR sRNAs, *cis*-antisense RNAs, riboswitches, and CRISPR RNAs.

#### Identification of *C. difficile* 6S RNA

*C. difficile* expressed one abundant not previously annotated sRNA (CD630_n00410, RCd3) corresponding to the widely distributed housekeeping 6S RNA. The TSS identified by our 5′-end RNA-seq analysis is preceded by consensus −35 and −10 promoter elements of the sigma A RNA polymerase holoenzyme (Figure 4A, Figure S2). Two RCd3 probe-hybridizing bands, of about 200 and 175 nt, were detected by Northern blotting (Figure 4B) as also observed in *Bacillus subtilis* and *S. pneumoniae* [40], [41]. These bands might correspond to a primary transcript and a processed form of *C. difficile* 6S RNA. Secondary structure prediction by Mfold [42] indicated that *C. difficile* RCd3 RNA can adopt a characteristic 6S RNA structure mimicking the structure of DNA in open promoter complex (Figure 4C) and tightly binding to the RNA polymerase holoenzyme [7], [43]. 6S RNA is present in various clostridial genomes. Our study provides the first experimental validation of the 6S sRNA expression in clostridia further confirming that global regulation of RNA polymerase activity by this sRNA is a widely conserved mechanism.

#### sRNAs regulated by growth phase

The synthesis of virulence factors in *C. difficile* is growth phase-dependent [26], [44], [45]. In particular, the entry into stationary phase is a signal that triggers expression of pathogenic determinants including toxins or adhesion and colonization factors [46]. Expression of six sRNAs detected by Northern blotting was growth phase-dependent (Table 3). Three sRNAs (CD630_n00210 (RCd4), CD630_n00680 (RCd5), and SQ1002) were induced at the onset of stationary phase, whereas the expression of three others (CD630_n00030 (RCd2), CD630_n00170 (RCd6), and SQ1498) was high during exponential phase and decreased at the onset of stationary phase. Figure 5 shows two examples of growth phase-regulated sRNAs: RCd6 located in the IGR between *CD0469* and *CD0470* genes encoding a saccharose-type IIABC component of the PTS system and a beta-lactamase-inducing penicillin-binding protein (BlaR), respectively, and SQ1498 located in IGR between *CD2112* and *CD2113* genes encoding a conserved hypothetical protein and a two-component sensor histidine kinase, respectively (Figure 5A). For RCd6 and SQ1498, ∼100 and 150-nt transcripts were detected by Northern blotting in accordance with sizes deduced from RNA-seq data (Figure 5B, Figure S3). Quantitative real-time PCR (qRT-PCR) analysis showed that RCd6 and SQ1498 were, respectively, 12- and 10-fold more abundant during exponential growth than in stationary phase (Table 3).

**Table 3:** Growth phase-regulated sRNAs.

Name	S/E fold change	Adjacent gene	Strand	5′_start	Score	Promoter	3′_end	Size, nt	Northern blot, nt	Rfam search
RCd4*	3.2	*CD0550* membrane protein	+	655072	2.94		655670	598		0
SQ1002*	2.8	*CD1518 feoA2*	−	1761105	1.24	Sigma A TATAAT 17 bp TTGACA	1760892	213	200	0
RCd5*	199	*CD1981* transcriptional regulator	+	2285913	14.43	Sigma A TATTAT 17 bp TTGAAA	2286311	398		GEMM RNA motif
RCd2*	0.14	*CD0183* cell wall hydrolase	−	241078	1.19	Sigma A TATAGT 18 bp TTGTAA	240878	200	105, 150, 350	0
RCd6*	0.08	*CD0470 blaR*	−	560340	1.24	Sigma A TATATA 17 bp TTAAAT	560200	140	100	0
SQ1498*	0.1	*CD2112* chp	−	2441927	1.51	-10 TATAAA	2441771	156	150	0

S, RNA extracted from cells at the onset of stationary phase (10 h of growth); E, RNA extracted from cells grown to exponential phase (4 h of growth). The S/E fold change was determined by qRT-PCR analysis. The position of 5′ start was identified by 5′-end RNA-seq analysis with indicated score corresponding to the read length (51 bases) coverage ratio for TAP^+^ and TAP^−^ sample. The position of 3′-end was deduced from RNA-seq data.

* The positions of 5′-start and/or 3′-end of these sRNAs were confirmed by 5′RACE or 5′/3′RACE analysis (Table S4). The presence of −10 and −35 boxes for sigma A-dependent promoters is shown. The size of the transcripts detected in Northern blot is indicated. RNA sequences were analysed for matches in Rfam database (http://rfam.sanger.ac.uk/), “0”, no matches. “chp” conserved hypothetical protein.

For four out of six growth phase-regulated sRNAs, the sigma A-dependent promoters were identified upstream of the TSS determined by 5′-end RNA-seq and confirmed by 5′RACE or 5′/3′RACE (Table 3, Table S4), while only a −10 promoter element is present upstream of SQ1498. The presence of sigma A recognition sequences suggests that growth phase-dependent control of transcription of these sRNAs does not involve the recruitment of RNA polymerase holoenzyme containing alternative sigma factors like SigH, the key *C. difficile* sigma factor during the transition phase [45]. Interestingly, we observed a 3-fold up-regulation of SQ1498 and a 7-fold down-regulation of RCd5 in a *sigH* mutant compared to 630Δ*erm* strain suggesting an indirect control by SigH. Two transcriptional regulators that control pre- or post-exponential events have been described in *C. difficile*: CodY, a global regulator monitoring the nutrient sufficiency of the environment [44] and Spo0A, a response regulator involved in the initiation of sporulation [47]. Only RCd6 was down-regulated about 2-fold in the *spo0A* mutant (data not shown). Thus, the mechanisms of regulation of these six growth-phase dependent *C. difficile* sRNAs remain to be determined.

With the exception of a c-di-GMP-dependent riboswitch, the search with Rfam database did not allow us to assign a function for the growth phase-regulated *C. difficile* sRNAs (Table 3). Since four growth phase-regulated sRNAs are encoded in IGRs, they may regulate their targets *in trans* by base-pairing with 5′UTRs of target genes mRNAs or coding regions. Three available softwares (see Materials and Methods section) predicted several common potential mRNA targets for each of these sRNAs. However, the relevance of these predictions needs further experimental investigations. Interestingly, secondary structure prediction by Mfold revealed that both growth phase-regulated sRNAs RCd6 and SQ1498 are highly structured. In RCd6, the predicted target base-pairing region is located in a bubble within the predicted secondary structure and might be therefore available for target interaction (Figure 5B). To test the effect of overexpression or depletion of these two growth phase-regulated sRNAs on the abundance of predicted target gene mRNAs, we constructed *C. difficile* strains carrying sRNA regions in sense or antisense orientation under the control of inducible *P_tet_* promoter of the pRPF185 vector derivative (Table S6). In the presence of the ATc inducer, up to 100-fold increase of RCd6 and SQ1498 sRNA levels was detected by qRT-PCR (data not shown). However, no detectable effects on the putative target genes mRNA levels or significant changes in *C. difficile* growth were observed (data not shown). One possibility would be that RCd6 and SQ1498 might affect the expression of their target genes at the translational initiation level as commonly found for known *trans* riboregulators [9]. Alternatively, *in silico* predictions may have failed to identify true RCd6 and SQ1498 targets. So, additional experiments will be required to assess the function of these sRNAs and their mechanisms of action.

The RCd2 sRNA is down-regulated at the onset of stationary phase. It is transcribed in an antisense orientation to two adjacent genes *CD0182* and *CD0183* and overlapped the 3′-end of *CD0182* (a gene of unknown function) and the 5′-end of *CD0183* (encodes a putative cell wall hydrolase) (Figure 3B and Figure 6A). Northern blotting reveals two major transcripts (∼150 and 350 nt), hybridizing with an RCd2-specific probe (Figure 6C). Identification of the RCd2 sRNA ends by 5′/3′RACE confirmed the position of TSS identified by 5′-end RNA-seq and precisely positioned the 3′-ends 175 nt and 350 nt downstream from TSS. Thus, both RCd2 transcripts overlap by 53 nt with the *CD0183* mRNA, while only the longer RCd2 transcript overlaps by 108 nt with the coding region of *CD0182* (Figure 3B and Figure 6A, Table S4). Overexpression of RCd2 led to a dramatic growth defect (Figure 6B), prompting us to analyse this RNA in more detail. *C. difficile* strains 630/p (carries the pRPF185 vector), 630/pRCd2 (overexpresses RCd2 from the *P_tet_* inducible promoter of pRPF185), and 630/pAS-RCd2 (overexpresses RCd2 in antisense orientation) (Table S6) grew equally well in TY medium in the absence of ATc inducer. In the presence of inducer, the doubling time of 630/pRCd2 increased more than 3-fold compared to that of 630/p or 630/pAS-RCd2 (Figure 6B and data not shown). The growth yield of 630/pRCd2 strain in the presence of ATc was also greatly reduced. A qRT-PCR analysis showed up to 300-fold induction of RCd2 expression in 630/pRCd2 compared to 630/p. Northern blot analysis of RNA extracted from induced 630/pRCd2 strain revealed several abundant major transcripts of about 105, 150, and 350 nt that hybridized with a RCd2-specific probe (Figure 6C). Similar transcripts were detected in 630Δ*erm* or 630/p strains (Figure 6C).

The effect of RCd2 overexpression on possible target genes was analysed by Northern blots with probes specific for *CD0182* or *CD0183*. While the *CD0182* mRNA was indetectable at conditions used, we observed a band of about 1000-nt with the *CD0183*-specific probe corresponding to the expected mRNA length (Figure 6C). Interestingly, this transcript was more abundant in 630/pRCd2 strain compared to 630/p. The apparent positive effect of RCd2 overexpression on the abundance of the *CD0183* transcript was further confirmed by qRT-PCR (>9-fold increase in 630/pRCd2 compared to 630/p). One hypothesis would be that the possible base-pairing between the 5′-parts of RCd2 RNA and *CD0183* mRNA (Figure 6A, 6D) may stabilize the target mRNA by protecting it from ribonucleolytic attacks. Several bacterial sRNAs are known to activate target gene expression by direct or indirect mechanisms including translational activation and mRNA stabilization [48], [49]. However, further experiments would be needed to precisely determine the molecular mechanism of this sRNA action.

Interestingly, the *CD0183* gene encodes a putative cell wall hydrolase that shares 38% identity with a *L. monocytogenes* autolysin, which promotes bacterial infection *in vivo* and modulates the interaction with the host immune system [50], [51], and 28% identity with *B. subtilis* LytE autolysin involved in cell wall turnover, cell separation during vegetative growth, and heat survival [52], [53]. The severe growth deficiency observed upon RCd2 overexpression may be explained by postulating that increased abundance of the *CD0183* transcript leads to enhanced cell autolytic activities as suggested by the presence of cell debris observed by optic microscopy (data not shown). Indeed, *CD0183* overexpression leads to growth defect in *E. coli* and *C. difficile* (data not shown). Thus, RCd2 sRNA may represent an example of RNA-dependent control of autolysin function in *C. difficile*.

### Coordinated regulation by c-di-GMP riboswitches in *C. difficile*

Another class of riboregulators detected by our analysis includes riboswitches responding to c-di-GMP. C-di-GMP is a signaling molecule with important functions in microbiological systems controlling lifestyle switches from free-living motile state to biofilm communities and virulence in Gram-negative bacteria [54]. The c-di-GMP molecule is synthesized from 2 GTP by diguanylate cyclases and is degraded into pGpG or 2GMP by phosphodiesterases. Effector molecules bound by this messenger include transcriptional factors and other proteins on the one hand, and RNA molecules that act as riboswitches on the other hand [55]. In contrast to most Gram-positive bacteria including some closest relatives, *C. difficile* encodes a large number of c-di-GMP turnover enzymes (up to 37) [56]. In addition, 12 type I c-di-GMP specific riboswitches (c-di-GMP-I) carrying conserved GEMM RNA domain [15] and 4 type II c-di-GMP dependent riboswitches (c-di-GMP-II) carrying a distinct RNA motif triggering an allosteric self-splicing ribozyme activity have been predicted in *C. difficile* [13], [14].

RNA-seq detected the expression of all *C. difficile* c-di-GMP riboswitches (Table S7). The analysis of chromosomal organization of the regions carrying c-di-GMP riboswitches revealed several interesting features. Two adjacent genes *CD2830* (encodes a precursor of exported protein) and *CD2831* (encodes a precursor of a collagen-binding protein) are controlled by c-di-GMP-I and c-di-GMP-II riboswitches (Cdi1_12 and Cdi2_3), respectively. Two c-di-GMP-I riboswitches (Cdi1_4 and Cdi1_5) are located in distant loci within the *C. difficile* 630 genome in highly similar prophage regions (*CD0904-979* and *CD2889-2952*) corresponding to host cell lysis module of bacteriophage PhiCD119. Therefore, c-di-GMP may control two homologous prophage genes *CD2889* and *CD0977.1*. Four other regions carrying c-di-GMP-I riboswitches (Cdi1_8, Cdi1_9, Cdi1_10 and Cdi1_11) are also highly homologous to each other (from 88 to 93% nucleic acid sequence identity) and must have arisen through multiple duplication events. They are located upstream of genes encoding conserved hypothetical proteins of unknown function (*CD1990.3*, *CD2309, CD1424* and *CD3368.2*, respectively).

#### Functionality of c-di-GMP type I riboswitches

One c-di-GMP-I riboswitch (Cdi1_3) is found upstream of the large *flgB* operon involved in the synthesis of basal body, hook and motor components of flagella and flagella specific sigma factor, SigD [15]. The presence of this regulatory RNA element within the 5′UTR of *flgB* was predicted *in silico* (SQ173 RNA) and experimentally confirmed by RNA-seq (Table S3). The precise location of the *flgB* TSS was determined by 5′-end RNA-seq data and further confirmed by 5′RACE (Table S4, Figure S4, Table S7). In accordance with the RNA-seq pattern, Northern blotting with the SQ173-specific probe revealed the presence of a major ∼160-nt transcript corresponding to premature termination of transcription upstream of the *flgB* operon (Figure 7A, Figure S4). It is intriguing that the promoter is located 496 bp upstream of the *flgB* start codon and that the terminated transcript is rather small (160 nt). However, by gene-specific RT-PCR we have clearly demonstrated the co-transcription of Cdi1_3 with *flgB* (Figure S4). The role of this long 5′UTR of *flgB* deserves further investigation.

To analyse the relevance of c-di-GMP signaling in *C. difficile*, we cloned the *CD1420-dccA* gene encoding a c-di-GMP synthetase [57], under the control of the inducible *P_tet_* promoter of plasmid pRPF185 (Table S6). In the presence of inducer, the *CD1420* transcript level was up to 1000-fold higher in strain 630/p*dccA* carrying pRPF185-*CD1420* than in strain 630/p carrying the pRPF185 vector. We therefore infer that the level of c-di-GMP should be considerably higher in induced 630/p*dccA* than in the control strain or in the absence of induction as demonstrated independently with another system of expression [57]. In accordance with the presence of a c-di-GMP riboswitch upstream of the *flgB* flagella assembly operon, the overexpression of *CD1420* led to a severe decrease of motility (Figure 7B). In addition, qRT-PCR analysis revealed a three-fold decrease in expression of *flgB* and *fliC* genes in induced 630/p*dccA* strain compared to 630/p (Figure 7B). The *fliC* gene encoding the main structural component of flagella filament, flagellin, is located within a distinct operon that might be indirectly controlled by c-di-GMP for example via *fliA*, a gene encoding flagella-specific sigma factor SigD, required for flagella gene expression in other bacteria and located within the *flgB* operon.

To further demonstrate the functionality of c-di-GMP riboswitches in *C. difficile*, we used the *CD1420*-overexpressing 630/p*dccA* strain to analyse the effect of c-di-GMP levels on gene expression by Northern blotting and qRT-PCR (Figure 7C and 7D, Table S7). For c-di-GMP type I riboswitches Cdi1_9 and Cdi1_12, two different transcripts were detected by Northern blotting with riboswitch-specific probes, the smaller ones corresponding to premature termination of transcription and the longer ones corresponding to transcripts of targeted genes, *CD2309* or *CD2830*, respectively. Excess c-di-GMP should bind to the leader region of mRNA at the riboswitch motif, leading to premature termination of transcription and decrease in transcriptional read-through necessary for full-length mRNA production. Indeed, upon induction of *CD1420* expression in 630/p*dccA*, we observed the repression of *CD2309* and *CD2830* full-length transcripts compared to strain 630/p (Figure 7C). Interestingly, in strain 630/p*dccA*, we observed in addition to a terminated transcript of 140 nt (Cdi1_9) or 170 nt (Cdi1_12) smaller bands with higher intensities that may represent the products of ribonucleolytic cleavage of the terminated transcript initiated in the presence of high c-di-GMP concentrations. Indeed, specific ribonucleases including RNase Y, RNase P, RNases J1 and J2 have been shown to cleave riboswitches or T-box motifs to initiate their ligand-dependent turnover in several bacteria [58]–[60]. The homologous RNase genes are present in *C. difficile* genome and may be also involved in riboswitch processing. Finally, a more general qRT-PCR analysis revealed the c-di-GMP-dependent negative control for all but one of the 12 c-di-GMP type I riboswitches in *C. difficile* (Table S7). This “OFF” riboswitch function corresponding to premature termination of transcription in the presence of elevated concentrations of c-di-GMP has already been demonstrated by transcriptional fusion analysis in heterologous system and by *in vitro* assays for Cdi1_3 [15]. For the Cdi1_1 riboswitch located upstream of the *CD1990* gene encoding a conserved protein of unknown function, we observed a 10-fold induction in 630/p*dccA* strain as compared to control strain suggesting that this regulatory RNA element functions through a mechanism different from that used by other c-di-GMP type I riboswitches.

#### Functionality of c-di-GMP type II riboswitches

*In vitro* and gene expression studies in a heterologous host suggest that the expression of *C. difficile* genes regulated by type II c-di-GMP riboswitches is induced in the presence of high c-di-GMP concentrations by a complex mechanism of alternative splicing positively modulating translation [13], [14]. In our work, for two type II c-di-GMP riboswitches examples shown in Figure 7D (Cdi2_4 and Cdi2_3), the full-length transcript of the targeted gene, *CD3513* or *CD2831*, respectively, was observed in Northern blots with probes hybridizing with riboswitch motif only in 630/p*dccA* strain overexpressing *CD1420, i.e.*, at conditions of elevated c-di-GMP levels. Presumably, active translation of mRNA might lead to stabilization of the full-length transcript. Conversely, at low c-di-GMP concentrations, the translation of mRNA might be inhibited leading to transcript destabilization. Several bands of smaller size detected under all conditions tested could correspond either to stable spliced forms or to forms produced by GTP attack [13], [14]. qRT-PCR analysis confirmed the c-di-GMP-dependent positive control for all four type II c-di-GMP riboswitches with up to 10-fold change of expression levels between 630/p*dccA* and 630/p strains (Figure 7B, Table S7). In addition, the corresponding genes were only barely expressed both during exponenthial growth and at the onset of stationary phase in liquid culture (Figure 7D). The overexpression of *CD1420* led to a greatly increased biofilm formation (Figure 7B) and cell aggregation (data not shown). The genes positively regulated by c-di-GMP through type II riboswitches encode putative pilin, and adhesion and surface proteins that could be involved in biofilm formation (Figure 7B). In accordance with phenotypic changes induced by c-di-GMP (Figure 7B), we hypothesize that *C. difficile* ensures coordinated control of motility and biofilm formation and other related processes through this use of two distinct - type I and type II - riboswitches responding to the same molecule, c-di-GMP. Altogether, our data provide experimental evidence for the functionality of c-di-GMP riboswitches in *C. difficile* and further highlight the crucial role of this second messenger in this pathogen.

### Identification and expression analysis of CRISPR loci

Our deep sequencing analysis revealed the expression of an additional class of regulatory RNAs, CRISPR RNAs, the products of a recently discovered prokaryotic adaptive immunity system for defense against foreign nucleic acids [8]. CRISPR arrays (also called “cassettes”) are composed of almost identical direct repeats separated by similarly sized variable sequences called spacers that often match viral or plasmid DNA. A CRISPR array is transcribed into a single RNA transcript (pre-crRNA), which is cleaved by the associated Cas proteins to generate small CRISPR RNAs (crRNAs). Mature crRNAs bind Cas proteins and serve as guides to recognize foreign nucleic acids by complementary base-pairing (Figure 8A). This recognition leads to degradation of target nucleic acid and protection of bacterial cell from invasion by foreign DNA.

CRISPRdb database predicted the existence of 12 CRISPR cassettes in the genome of *C. difficile* 630 strain with a variable number of conserved 29-bp direct repeats separated by 37-bp spacers [61]. Only two cassettes (CRISPR 12 and CRISPR 17) are associated with *cas* genes. CRISPR 17 cassette carries the largest number of spacers and is associated with a complete set of *cas* genes; CRISPR 12 is associated with an incomplete *cas* gene set (Table S8). According to the recent classification of CRISPR-Cas systems, *C. difficile* posseses a Type I-B (subtype CASS7) CRISPR-Cas system [62]. The high sequence conservation among repeats suggests that RNA transcribed from CRISPR cassettes lacking associated *cas* genes might be processed by Cas proteins encoded by the operons associated with CRISPR 12 and/or 17. Our RNA-seq analysis revealed the expression of all 12 CRISPR loci in *C. difficile* 630Δ*erm*. 5′-end RNA-seq analysis allowed precise identification of the TSS for all CRISPR cassettes and most of them were associated with potential sigma A-dependent promoters (Table S8). In all cases, the presence of a “leader” 5′ region located between the TSS and the first repeat of CRISPR cassette was observed (Figure 8A, Figure S5).

Five *C. difficile* CRISPR cassettes are located within prophage regions, and three of them are highly expressed (Table S8) as exemplified by Northern blotting for CRISPR 16 cassette in Figure 8B. Using a probe complementary to the first spacer sequence of CRISPR 16, we can clearly distinguish, by Northern blotting, full-length pre-crRNA transcripts of up to 1000 nt in length, incompletely processed crRNA of various sizes, and, finally, ∼66-nt mature crRNA (Figure 8B). The apparent size of the largest transcript detected in Northern blotting (Table S8, Figure 8B, Figure S5) suggested that CRISPR 15 and 16 cassettes may be co-transcribed. In fact, this entire region is duplicated in the *C. difficile* 630 genome (giving raise to CRISPR 3 and 4, and CRISPR 15 and 16 cassettes, Table S8). The 5′-end RNA-seq and RNA-seq profile for CRISPR 15/16 locus is shown in Figure S5, the number of distinct read patterns is in accordance with the number of repeats in the locus.

The deep sequencing data indicated that crRNAs from the “leader”-proximal regions of CRISPR loci (Figure S5) were more abundant than crRNAs encoded further downstream, as it was also observed in *Pyrococcus furiosus* [63]. This gradient may reflect differences in transcription, processing, and/or stability. This observation raises then a question about the relevance of such differential crRNA expression level for the functional activity and dynamics of CRISPR-Cas system, where the most recently acquired spacers are located next to the “leader” region [64]. In addition, the deep sequencing profile and the size of crRNAs detected by Northern blot are both in accordance with a recently published cleavage pattern for a CRISPR-Cas subtype I-B [65]. Our experimental detection of crRNAs is the first demonstration of the existence of multiple CRISPR cassettes expressed in pathogenic clostridia in contrast to the presence of silent or only barely expressed additional CRISPR loci reported in some other bacteria including *S. pyogenes* and *E. coli* [66], [67].

The presence of several functional CRISPR cassettes showing different level of expression raises a question on the respective role of each CRISPR in effective invader defense under different conditions. Among the interesting features of the CRISPR-Cas system in *C. difficile*, the location of some CRISPR arrays within prophage regions suggests an efficient strategy for use of CRISPR to limit the dispersal of competing phages [68].

### Conclusion

In this study, we combined *in silico* predictions, global high-throughput sequencing, and gene-targeted experimental approaches to identify sRNAs in the human pathogen *C. difficile*. A total of about 250 sRNAs including sRNAs from IGRs, *cis*-antisense RNAs, and riboswitches are experimentally detected in this pathogen. This number is close to 200–300 sRNA genes estimated to be present in an average bacterial genome [69]. However, the number revealed by our work is almost certainly an underestimation, since some RNA regulators could be expressed at conditions other than those analysed in our work. Since only a small fraction of sRNAs identified in this study belongs to known functional families, the majority of discovered *C. difficile* sRNAs could represent novel riboregulators.

In contrast to Gram-negative bacteria, the conservation of sRNAs seems to be mostly confined to the genus or even species level in Gram-positive bacteria as has been recently shown for *B. subtilis* SR1 homologues [40], [70], [71]. In accordance with these observations, most of sRNAs identified in this study are common to *C. difficile* strains but are absent from other clostridia. This may be explained by very high heterogeneity of the Clostridium group, which is a consequence of a wide variety of ecological niches and distinct evolutionary pathways followed by different clostridial species. Thus, our results further underscore the importance of searches for regulatory RNAs in individual bacterial species for a better understanding of their role in the physiology and the infection cycle control. Moreover, the discovery of novel sRNAs by our approaches emphasizes the potential diversity of sRNAs in clostridia.

Several features of newly identified sRNAs suggest the importance and diversity of RNA-based mechanisms controlling key steps during infection cycle of *C. difficile*. The presence of a great number of functional riboswitches suggests a wide use of 5′-*cis*-RNA regulatory elements that ensure rapid and adequate response of *C. difficile* to changing conditions allowing better adaptation of metabolic pathways. Here, we demonstrated the functionality of a particular class of such riboregulators, riboswitches responding to c-di-GMP. In most microorganisms, proteins are effectors for c-di-GMP signaling pathways. In *C. difficile*, the use of RNA riboswitches as effectors sensing c-di-GMP further supports the importance of RNA-based regulatory mechanisms in this bacterium. These riboswitches might be important for coordinated control of many processes crucial for successful development of *C. difficile* inside the host such as adhesion, colonization, biofilm formation, motility and other related processes.

Several sRNAs discovered in this study showed growth phase-dependent expression profile being either induced or repressed during transition from exponential growth to stationary phase. This includes an interesting case of an IGR-located *cis*-antisense RNA overlapping two adjacent genes that may be involved in the control of autolytic activity in *C. difficile*. The synthesis of virulence determinants and associated factors in *C. difficile* is also growth phase-dependent [26], [45], [46]. One can speculate that some of growth phase-regulated *C. difficile* sRNAs may be involved in this control, as previously observed in other Gram-positive and in Gram-negative bacteria [3], [27], [72].

An important finding of this work is the demonstration that multiple CRISPR arrays are expressed in *C. difficile*. Foreign DNA defense capacities provided by CRISPR-Cas system might be important for *C. difficile* survival within bacteriophage-rich gut communities and also for the control of genetic exchanges favored within gut microbiota [73]–[75].

In summary, this study constitutes a first step towards better understanding of complex sRNA-based regulatory networks governing *C. difficile* physiology and pathogenesis. Further investigations based on our findings will help to determine the exact biological roles of many of these sRNAs and to uncover the diverse molecular mechanisms of RNA-based regulation employed by *C. difficile* to adapt to various environments it encounters outside and inside the host.

## Materials and Methods

### Plasmid and bacterial strain construction and growth conditions

*C. difficile* strains and plasmids used in this study are presented in Table S6. *C. difficile* strains were grown anaerobically (5% H_2_, 5% CO_2_, and 90% N_2_) in TY [76] or Brain Heart Infusion (BHI, Difco) media, which was used for conjugation. When necessary, Cefoxitin (Cfx; 25 µg/ml) and Thiamphenicol (Tm; 15 µg/ml) were added to *C. difficile* cultures. *E. coli* strains were grown in Luria-Bertani (LB) broth, and when indicated, ampicillin (100 µg/ml) or chloramphenicol (15 µg/ml) was added to the culture medium. The non-antibiotic analog anhydrotetracycline (ATc; 250–500 ng/ml) was used for induction of the *P_tet_* promoter of pRPF185 vector derivatives in the *C. difficile* [77].

Motility was tested on semi-solid BHI medium (0.17% agar) containing 8 µg/ml Tm and 500 ng/ml ATc. Strains were inoculated into semi-solid medium tubes and incubated for at least 24 h at 37°C. Biofilm formation was assayed as previously described [78].

All routine plasmid constructions were carried out using standard procedures [79]. All primers used in this study are listed in Table S9. For inducible expression of *C. difficile* genes, we used the pRPF185 vector expression system carrying a *gusA* gene between an ATc-inducible *P_tet_* promoter and a transcriptional terminator [77]. The SQ1498 region (−6 to +272 relative to the TSS of the sRNA), RCd6 (−2 to +146), RCd2 (+1 to +391) and the *CD1420* gene (−15 to +843 relative to the translational start site) were amplified by PCR. To investigate the effects of down-regulation of expression of SQ1498, RCd6 and RCd2, antisense fragment to these sRNA genes was also cloned under the control of the inducible *P_tet_* promoter. We used primers containing a 5′-BamHI or a 3′-SacI site for sense and 5′-SacI or a 3′-BamHI site for antisense cloning orientation. These fragments were inserted between the BamHI and SacI sites of pRPF185 vector. For sRNA overexpression the pRPF185 vector was modified by reverse PCR approach with OS618 and OS620 primers to allow cloning of sRNA region directly between *P_tet_* promoter and vector-borne terminator. The *CD0183* gene was cloned into StuI and BamHI sites of pRPF185Δ*gusA* derivative carrying SacI, StuI, XhoI and BamHI cloning sites instead of *gusA* region. DNA sequencing was performed to verify plasmid constructs using pRPF185 specific primers IMV507, OS621 and IMV508. The resulting derivative pRPF185 plasmids were transformed into the *E. coli* HB101 (RP4) and subsequently mated with *C. difficile* 630Δ*erm* [80] (Table S6). *C. difficile* transconjugants were selected by sub-culturing on BHI agar containing Tm (15 µg/ml) and Cfx (25 µg/ml).

### RNA extraction, quantitative real-time PCR, and Northern blot analysis

Total RNA was isolated from *C. difficile* 630Δ*erm* strain grown in TY medium either after 4 h, 6 h or 10 h of growth and from R20291 strain during late exponential growth phase (6 h) as previously described [22]. Starvation conditions correspond to a 1 h incubation of exponentially grown cells (6 h of growth) in PBS buffer at 37°C. Strains carrying pRPF185 derivatives were grown in TY medium in the presence of 250–500 ng/mL ATc and 7.5 µg/mL Tm for 7.5 h followed by RNA isolation. The cDNA synthesis by reverse transcription and quantitative real-time PCR analysis was performed as previously described [45]. In each sample, the relative expression for a gene was calculated relatively to the 16S gene [81]. The relative change in gene expression was recorded as the ratio of normalized target concentrations (ΔΔCt) [82]. Strand-specific RT-PCR analysis was performed as previously described [27].

For Northern blot analysis, 5 µg of total RNA was separated on a denaturing 6% or 8% polyacrylamide gel containing 8 M urea, and transferred to Hybond-N^+^ membrane (Amersham) by electroblotting using the Trans-blot cell from Bio-Rad in 1× TBE buffer (89 mM Tris-base, 89 mM boric acid and 2 mM EDTA). Following UV-cross-linking of the samples to the membrane, prehybridization was carried out for 2 h at 42°C in 7 mL of prehybridization buffer ULTRAHyb (Ambion). Hybridization was performed overnight at 42°C in the same buffer in the presence of a [gamma-^32^P]-labeled DNA oligonucleotide probe. Alternatively, the probe was synthesized using PCR with 5′end-labeled primer complementary to RNA sequence. After hybridization, membranes were washed twice for 5 min in 50 mL 2× SSC (300 mM sodium chloride and 30 mM sodium citrate) 0.1% sodium dodecyl sulphate (SDS) buffer and twice for 15 min in 50 mL 0.1× SSC 0.1% SDS buffer. Radioactive signal was detected with a Typhoon system (Amersham). The size of the transcripts was estimated by comparison with RNA molecular weight standards (Invitrogen).

### 5′RACE and 5′/3′RACE

5′RACE (Rapid amplification of cDNA ends) assays were performed on total RNA extracted from *C. difficile* 630Δ*erm* strain using a 5′RACE System kit (Invitrogen) as previously described [22]. 5′/3′RACE was performed essentially as previously described [60] with some modifications. Total RNA (5 µg) was treated with TAP (Epicentre) for 1 h at 37°C. After phenol/chloroform extraction, followed by ethanol precipitation, RNA was circularized with T4 RNA ligase (Biolabs) overnight at 16°C. Circularized RNA was again extracted with phenol/chloroform, ethanol precipitated and subjected to RT–PCR across the 5′/3′ junction. The PCR products were then cloned using the pGEM-T easy system (Promega) and sequenced.

### RNA–seq and 5′-end RNA–seq experiments

Non-orientated library for whole transcript analysis by RNA-seq was realized on the RNA sample extracted from *C. difficile* 630Δ*erm* strain during late exponential growth phase (6 h) as previously described [83]. TAP converts 5′-triphosphates into 5′-monophosphates allowing Illumina adaptor ligation to RNA 5′-ends before cDNA synthesis and thus the enrichment with primary transcript reads. The TAP+ and TAP- library construction for high-throughput sequencing (differential 5′-end RNA-seq) was realized on a mixed sample combining RNAs extracted from three different growth conditions including exponential growth (4 h), entry to stationary phase (10 h) and nutriment starvation (1 h incubation in PBS buffer). 15 µg of total RNA treated with TurboDNAse (Ambion) was used for depletion of ribosomal RNA with the MicrobExpress kit (Ambion) following the manufacturer instructions. RNA depleted for rRNA was divided into two similar fractions and 1500 ng of this partially purified mRNAs was used for each library preparation. To convert the 5′PPP ends in 5′P ends, RNA was denatured 10 min at 65°C, placed on ice and incubated 1 h at 37°C with 10 units of TAP (Epicentre) (TAP+ library). For TAP- library construction, RNA depleted for rRNA was incubated with buffer alone under the same conditions. Products were purified by phenol/chloroform extraction and ethanol precipitation. cDNA library construction for Illumina sequencing was performed as previously described [72]. 51-bp sequences were generated via Illumina HiSeq 2000 sequencing machine.

### Sequencing data processing

The Illumina reads were first scanned for adaptor removal. Remaining sequencing reads were mapped to the *C. difficile* genome using Bowtie software (Options –sam -q -k 2000) [84] then converted into BAM files with the Samtools script [85] (Table S2). The data were visualized at a strand-specific manner (for 5′-end RNA-seq libraries) or as a whole transcript coverage map (for RNA-seq) using COV2HTML (Marc Monot, submitted). All TSSs detected by 5′-end RNA-seq were inspected manually to identify potential sRNAs. To estimate the relevance of each TSS, a score was computed which corresponds to the read length (51 bases) coverage ratio for sample with and without TAP treatment (Table S3). The transcript length of sRNAs was estimated by scanning the read coverage, produced by the whole-transcript sequencing (RNA-seq), downstream to the identified 5′-end. A sharp decline in the read coverage was considered the 3′-end of the transcript. In the absence of alternative experimental data for antisense transcripts overlapping coding regions or for barely detected transcripts the arbitrary length was fixed to 100 nt (Table S3).

### *In silico* screening for new sRNA genes and their feature analysis

The *in silico* screening of the genome of *C. difficile* 630 strain was done as previously described for *S. agalactiae* [27]. Briefly, the Rho-independent terminators were predicted along the full genome sequence with Transterm HP software with default parameters. Comparative genomics of the *C. difficile* 630 strain was done against all available sequences of clostridial strains in the GenBank database (March 2010). The prediction of secondary structure of each sRNA candidate was performed with Mfold 3.4 software [42]. All other steps were done as described without modifications.

To analyse possible coding capacities of sRNAs their sequences were scanned for the presence of protein coding sequences (CDSs) starting with ATG, TTG or GTG codons representing almost the totality of start codons identified in *C. difficile* genome [86]. The size of CDSs was limited to 45 bases without stop codon (TAG, TAA or TGA). Detected CDSs were analysed for the presence of putative ribosome binding site by searching Shine-Dalgarno sequences within 20 nucleotides upstream of start codon. Predicted CDSs were also tested using BLASTP for similarity with known proteins (*E*-value of 0.05 or less) and CDSs sized greater than 15 amino acids with no homologues were classified as possible novel proteins or peptides.

The conservation of sRNAs detected in this study was determined by comparing them with sequence homologues in 13 *C. difficile*, 14 other clostridial genomes and 5 additional bacterial genomes outside Clostridia. We used BLAST with standard parameters and selected matches corresponding to the alignments covering more than 80% of sRNA length (*E*-value of 0.001 or less). We performed a search for riboswitch conservation in the upstream regulatory regions of the orthologous genes from *C. difficile* strains (80% bidirectional orthology criteria) and other clostridial species (30% bidirectional orthology criteria) and selected matches corresponding to BLAST alignments covering more than 80% of riboswitch length for *C. difficile* strains and >30% for the other clostridia. The ORF conservation within *C. difficile* strains was analyzed by TBLASTX with standard parameters.

The Rfam database was used to search for known sRNAs [34]. In order to predict target mRNA for the identified sRNAs TargetRNA [87], IntaRNA [88] and RNAPredator [89] servers were used with standard parameters. The overlapping predictions with highest score were considered for experimental validation.

### Data access

Deep sequencing data are available at http://mmonot.eu/COV2HTML/visualisation.php?str_id=-13.

## Supporting Information

Figure S1 Experimental validation of *in silico* predicted sRNAs. RT-PCR was performed using gene-specific primers (Table S9) for SQ1002 (lanes 1 and 2), SQ2025 (lanes 3 and 4), SQ0367 (lanes 5 and 6), SQ0931 (lanes 7 and 8), SQ1498 (lanes 9 and 10), with RNA extracted from 630Δ*erm* cells grown to exponential phase (4 h of growth) (lanes 1, 3, 5, 7 and 9) or to the entry to stationary phase (10 h of growth) (lanes 2, 4, 6, 8 and 10). Negative control reactions without reverse transcriptase performed using the same gene-specific primers and RNA samples are shown in lanes 11–20.
(PDF)

Figure S2 Detection of abundant 6S RNA by deep sequencing. The TAP−/TAP+ profile comparison for 5′-end RNA-seq data is aligned with RNA-seq data for RCd3 (6S RNA) genomic region. The TSS identified by 5′-end sequencing are indicated by red broken arrows in accordance with the positions of 5′-transcript ends shown by vertical green lines on the sequence read graphs corresponding either to TSS (broken arrows) or to processing sites (vertical arrows). TSS corresponds to position with significantly greater number of reads in TAP+ sample, potential cleavage site corresponds to position with large number of reads in both TAP− and TAP+ samples. 5′-end sequencing data show 51-bp reads matching to the 5′-transcript ends, while RNA-seq data show reads covering whole transcript. Coding sequences are indicated by blue arrows and the 6S RNA is indicated by a grey arrow.
(PDF)

Figure S3 Expression analysis by deep sequencing of growth phase-regulated sRNAs. The results are given at the left for RCd6 sRNA and at the right for SQ1498. The TAP−/TAP+ profile comparison for 5′-end RNA-seq data is aligned with RNA-seq data for corresponding genomic region. The TSS identified by 5′-end sequencing are indicated by red broken arrows in accordance with the positions of 5′-transcript ends shown by vertical green lines on the sequence read graphs corresponding either to TSS (broken arrows) or to processing sites. TSS corresponds to position with significantly greater number of reads in TAP+ sample. 5′-end sequencing data show 51-bp reads matching to the 5′-transcript ends, while RNA-seq data show reads covering whole transcript. Coding sequences are indicated by blue arrows and new sRNA candidates identified in this study are indicated by grey arrows.
(PDF)

Figure S4 Co-transcription of SQ173 and *flgB* operon. TAP−/TAP+ (5′-end RNA-seq) profile comparison and RNA-seq data are given for SQ173 located upstream of the *flgB* flagella operon. The positions of the primers used for RT-PCR are indicated at the bottom of the *flgB* region diagram with the estimated length of corresponding fragments. RT-PCR was performed using gene-specific primers (Table S9) for SQ173 (Cdi1_3) and *flgB* with RNA extracted from 630Δ*erm* cells grown to exponential phase (4 h of growth) (lanes 1, 2, 3) or to the entry to stationary phase (10 h of growth) (lanes 4, 5, 6). Negative control reactions without reverse transcriptase performed using the same gene-specific primers and RNA samples are shown in lanes 7–12.
(PDF)

Figure S5 Expression analysis of CRISPR 16 cassette by deep sequencing. The TAP−/TAP+ profile comparison for 5′-end RNA-seq is aligned with RNA-seq data for corresponding genomic region. The TSS identified by 5′-end sequencing are indicated by red broken arrows in accordance with the positions of 5′-transcript ends shown by vertical green lines on the sequence read graphs corresponding either to TSS (broken arrows) or to processing sites. TSS corresponds to position with significantly greater number of reads in TAP+ sample, potential cleavage site corresponds to position with large number of reads in both TAP− and TAP+ samples. 5′-end sequencing data show 51-bp reads matching to the 5′-transcript ends, while RNA-seq data show reads covering whole transcript.
(PDF)

Table S1 Complete list of predicted sRNAs.
(PDF)

Table S2 Summary of sequenced 51-bp reads from NGS of *C. difficile*.
(PDF)

Table S3 Complete list of sRNA detected by deep sequencing.
(PDF)

Table S4 sRNA extremity identification by 5′RACE and 5′/3′RACE.
(PDF)

Table S5 Conservation of regulatory RNAs in bacterial strains.
(PDF)

Table S6 Strains and plasmids used in this study.
(PDF)

Table S7 C-di-GMP riboswitches.
(PDF)

Table S8 CRISPR arrays.
(PDF)

Table S9 Oligonucleotides used in this study.
(PDF)
