# Supplementary figures and images for: Genome-Wide Identification of Regulatory RNAs in the Human Pathogen Clostridium difficile
Source: PLoS Genet. 2013 May 9;9(5):e1003493. doi: 10.1371/journal.pgen.1003493 (PMC3649979; doi:10.1371/journal.pgen.1003493)

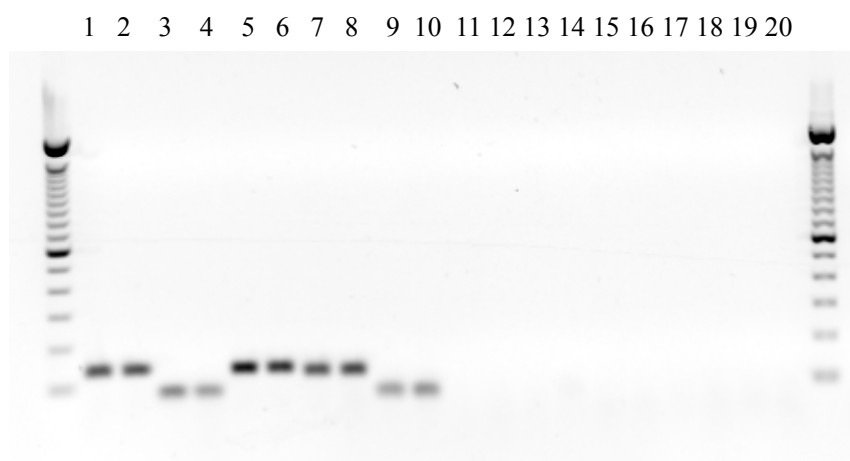

**Figure S1.**

Supplement: Figure S1 — Experimental validation of in silico predicted sRNAs. RT-PCR was performed using gene-specific primers (Table S9) for SQ1002 (lanes 1 and 2), SQ2025 (lanes 3 and 4), SQ0367 (lanes 5 and 6), SQ0931 (lanes 7 and 8), SQ1498 (lanes 9 and 10), with RNA extracted from 630Δerm cells grown to exponential phase (4 h of growth) (lanes 1, 3, 5, 7 and 9) or to the entry to stationary phase (10 h of growth) (lanes 2, 4, 6, 8 and 10). Negative control reactions without reverse transcriptase performed using the same gene-specific primers and RNA samples are shown in lanes 11–20. (PDF) [file pgen.1003493.s001.pdf]

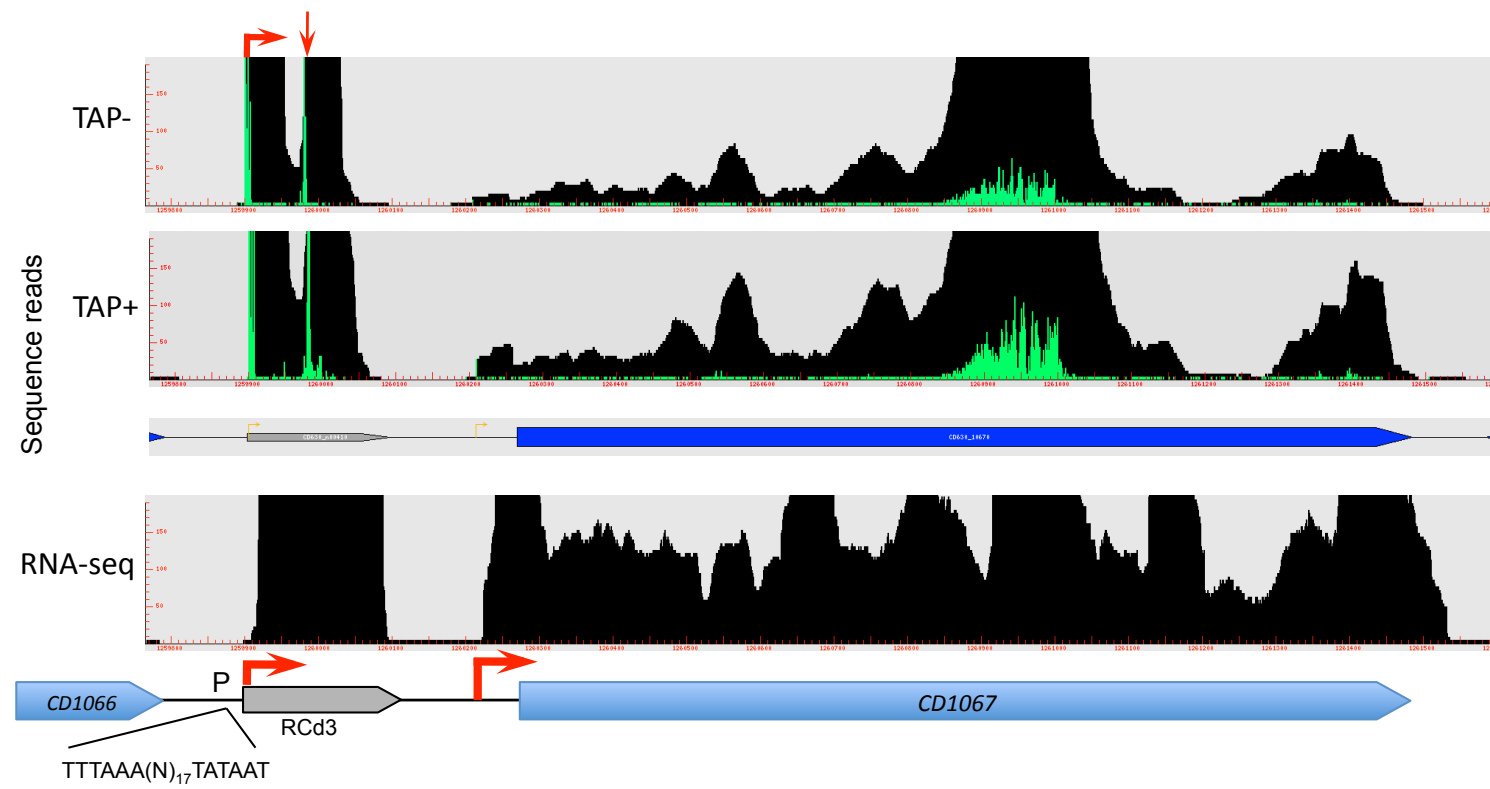

Figure S2.

Supplement: Figure S2 — Detection of abundant 6S RNA by deep sequencing. The TAP−/TAP+ profile comparison for 5′-end RNA-seq data is aligned with RNA-seq data for RCd3 (6S RNA) genomic region. The TSS identified by 5′-end sequencing are indicated by red broken arrows in accordance with the positions of 5′-transcript ends shown by vertical green lines on the sequence read graphs corresponding either to TSS (broken arrows) or to processing sites (vertical arrows). TSS corresponds to position with significantly greater number of reads in TAP+ sample, potential cleavage site corresponds to position with large number of reads in both TAP− and TAP+ samples. 5′-end sequencing data show 51-bp reads matching to the 5′-transcript ends, while RNA-seq data show reads covering whole transcript. Coding sequences are indicated by blue arrows and the 6S RNA is indicated by a grey arrow. (PDF) [file pgen.1003493.s002.pdf]

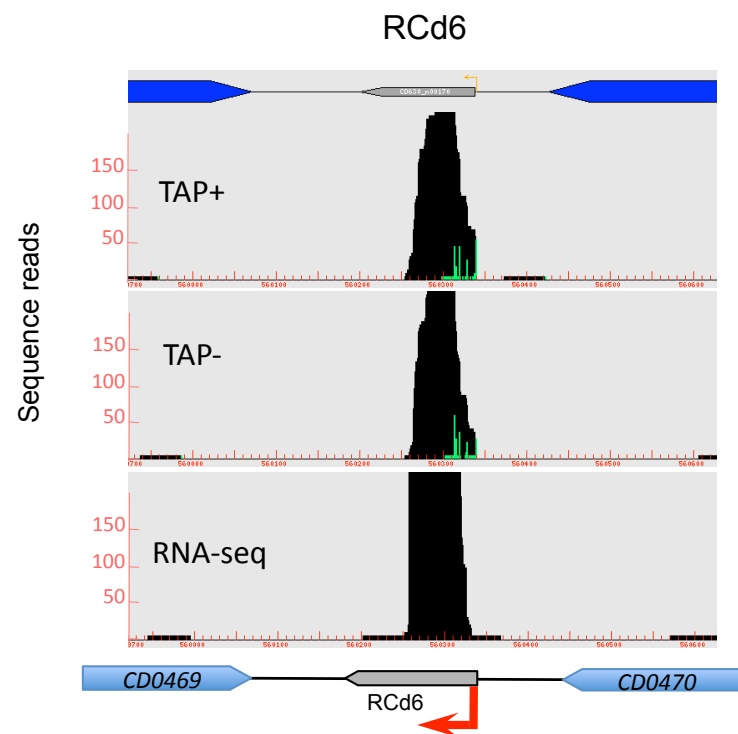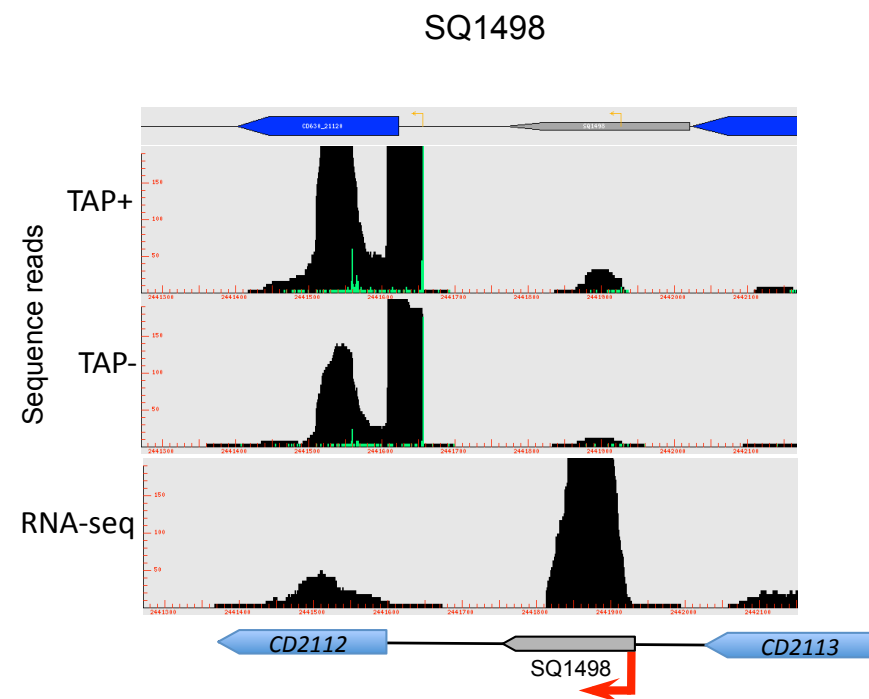

**Figure S3.**

Supplement: Figure S3 — Expression analysis by deep sequencing of growth phase-regulated sRNAs. The results are given at the left for RCd6 sRNA and at the right for SQ1498. The TAP−/TAP+ profile comparison for 5′-end RNA-seq data is aligned with RNA-seq data for corresponding genomic region. The TSS identified by 5′-end sequencing are indicated by red broken arrows in accordance with the positions of 5′-transcript ends shown by vertical green lines on the sequence read graphs corresponding either to TSS (broken arrows) or to processing sites. TSS corresponds to position with significantly greater number of reads in TAP+ sample. 5′-end sequencing data show 51-bp reads matching to the 5′-transcript ends, while RNA-seq data show reads covering whole transcript. Coding sequences are indicated by blue arrows and new sRNA candidates identified in this study are indicated by grey arrows. (PDF) [file pgen.1003493.s003.pdf]

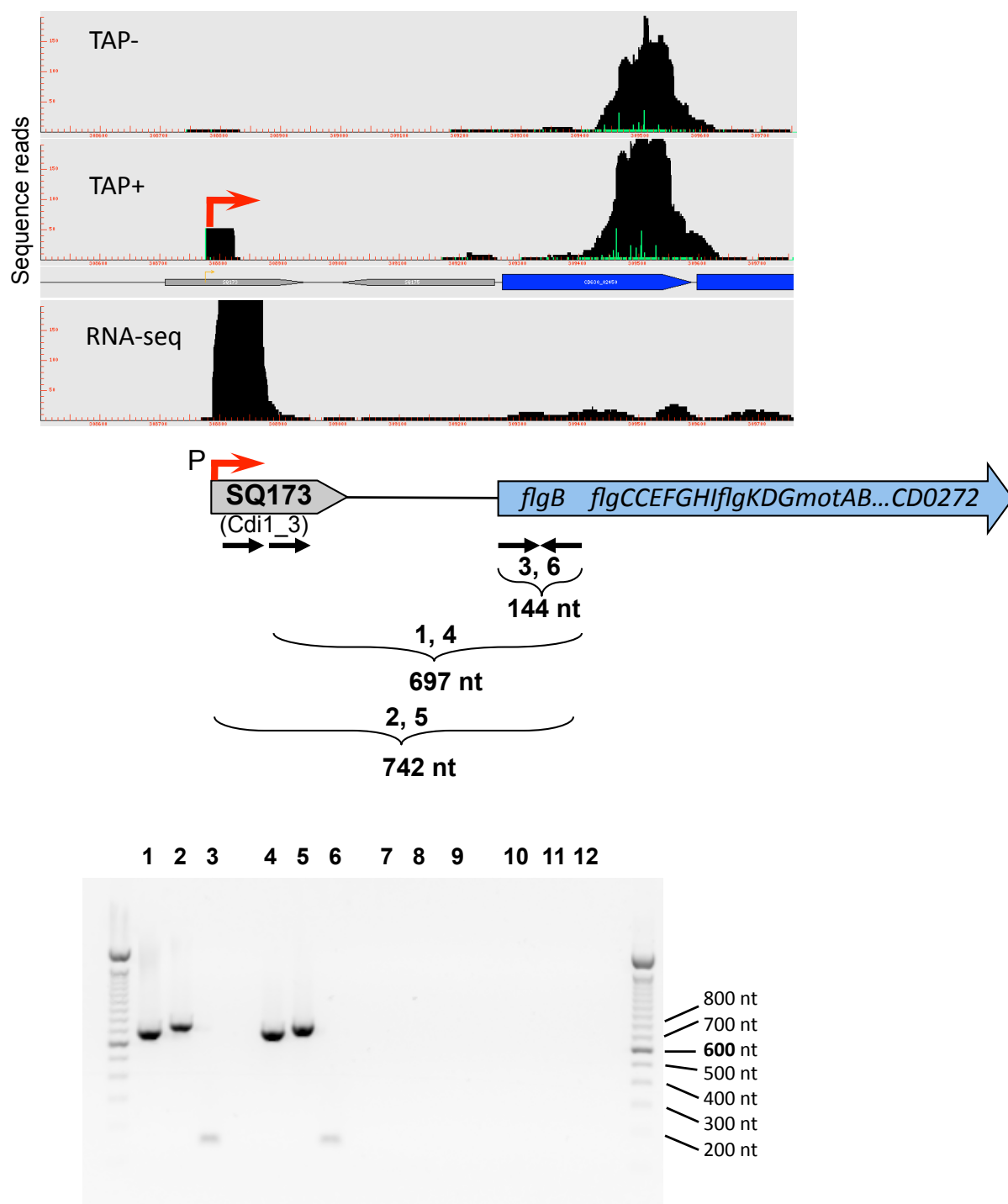

**Figure S4.**

Supplement: Figure S4 — Co-transcription of SQ173 and flgB operon. TAP−/TAP+ (5′-end RNA-seq) profile comparison and RNA-seq data are given for SQ173 located upstream of the flgB flagella operon. The positions of the primers used for RT-PCR are indicated at the bottom of the flgB region diagram with the estimated length of corresponding fragments. RT-PCR was performed using gene-specific primers (Table S9) for SQ173 (Cdi1_3) and flgB with RNA extracted from 630Δerm cells grown to exponential phase (4 h of growth) (lanes 1, 2, 3) or to the entry to stationary phase (10 h of growth) (lanes 4, 5, 6). Negative control reactions without reverse transcriptase performed using the same gene-specific primers and RNA samples are shown in lanes 7–12. (PDF) [file pgen.1003493.s004.pdf]

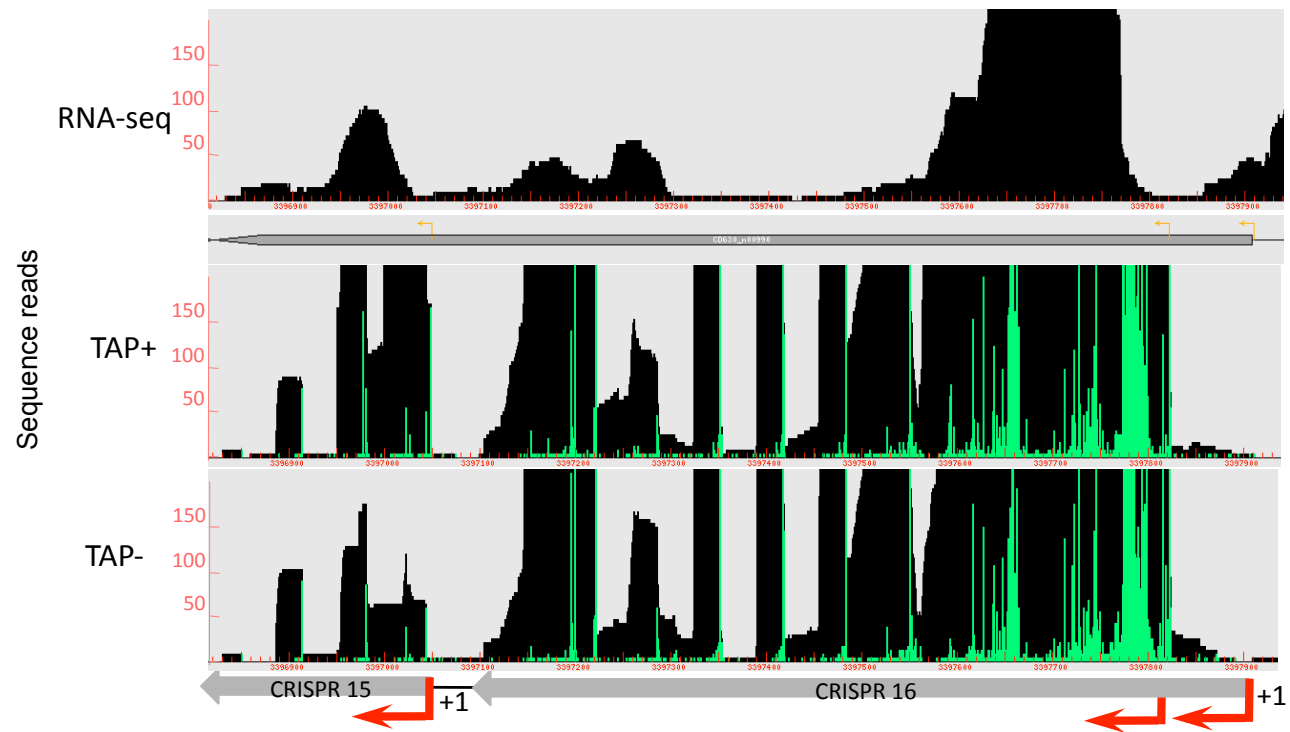

Figure S5.

Supplement: Figure S5 — Expression analysis of CRISPR 16 cassette by deep sequencing. The TAP−/TAP+ profile comparison for 5′-end RNA-seq is aligned with RNA-seq data for corresponding genomic region. The TSS identified by 5′-end sequencing are indicated by red broken arrows in accordance with the positions of 5′-transcript ends shown by vertical green lines on the sequence read graphs corresponding either to TSS (broken arrows) or to processing sites. TSS corresponds to position with significantly greater number of reads in TAP+ sample, potential cleavage site corresponds to position with large number of reads in both TAP− and TAP+ samples. 5′-end sequencing data show 51-bp reads matching to the 5′-transcript ends, while RNA-seq data show reads covering whole transcript. (PDF) [file pgen.1003493.s005.pdf]
