# Supplementary material for: Genome-Wide Identification of Regulatory RNAs in the Human Pathogen Clostridium difficile
Source: PLoS Genet. 2013 May 9;9(5):e1003493. doi: 10.1371/journal.pgen.1003493 (PMC3649979; doi:10.1371/journal.pgen.1003493)
Supplement: Table S1 — Complete list of predicted sRNAs. (PDF) [file pgen.1003493.s006.pdf]

Table S1. Complete list of predicted sRNAs

Name	5'_start	3'_end	Strand	Annotation1	Annotation2	Validation	Prediction by Chen et al.
SQ13	19529	19280	<	* Small RNA located in IGR between : CDS serS1(>) and CDS CD0015(>)	Putative sRNA candidate		sCD20
SQ40	71692	71426	<	* Small RNA located in IGR between : CDS CD0046(>) and CDS ispD(>)	Putative sRNA candidate		
SQ55	88961	88718	<	* Small RNA located in IGR between : CDS rplL(>) and CDS CD0065(>)	Putative sRNA candidate		
SQ54	88536	88776	>	* Small RNA located in IGR between : CDS rplL(>) and CDS CD0065(>)	Putative sRNA candidate		
SQ86	141186	140917	<	* Small RNA located in IGR between : CDS nrdG(>) and rRNA 16s_rRNA(>)	Putative sRNA candidate		
SQ98	153481	153737	>	* Small RNA located in IGR between : tRNA tRNA-Arg(>) and CDS CD0110(>)	Putative sRNA candidate		sCD153
SQ114	175832	175577	<	* Small RNA located in IGR between : CDS metE(>) and CDS CD0131(>)	Putative sRNA candidate		sCD175
SQ129	201768	201995	>	* Small RNA located in IGR between : CDS gcp(>) and CDS hpdB(>)	Putative sRNA candidate		
SQ131	206210	205962	<	* Small RNA located in IGR between : CDS hpdA(>) and CDS CD0156(>)	Putative sRNA candidate		
SQ154	249395	249134	<	* Small RNA located in IGR between : CDS CD0190(>) and CDS CD0191(>)	Putative sRNA candidate		sCD250
SQ155	252036	251786	<	* Small RNA located in IGR between : CDS cls(>) and CDS groES(>)	Putative sRNA candidate		
SQ158	254166	253905	<	* Small RNA located in IGR between : CDS groEL(>) and CDS CD0195(>)	Putative sRNA candidate		
SQ165	274849	274602	<	* Small RNA located in IGR between : CDS CD0210(>) and CDS licC(>)	Putative sRNA candidate		sCD275
SQ173	308709	308944	>	* Small RNA located in IGR between : CDS CD0244(>) and CDS flgB(>)	Putative sRNA candidate	NB, RT-PCR	sCD309.1
SQ175	309259	309001	<	* Small RNA located in IGR between : CDS CD0244(>) and CDS flgB(>)	Putative sRNA candidate		
SQ190	340758	341018	>	* Small RNA located in IGR between : CDS CD0279(>) and CDS CD0279A(>)	Putative sRNA candidate		sCD341
SQ203	356375	356132	<	* Small RNA located in IGR between : CDS CD0294(>) and CDS CD0295(>)	Putative sRNA candidate		
SQ205	359117	358864	<	* Small RNA located in IGR between : CDS CD0297(>) and CDS rbsR(>)	Putative sRNA candidate		
SQ227	399886	399635	<	* Small RNA located in IGR between : CDS ppiB(>) and CDS bclA1(>)	Putative sRNA candidate		
SQ268	477663	477890	>	* Small RNA located in IGR between : CDS fba(<) and CDS CD0404(>)	Putative sRNA candidate		
SQ292	521926	521672	<	* Small RNA located in IGR between : CDS CD0439(>) and CDS CD0440(>)	Putative sRNA candidate		
SQ297	526046	525811	<	* Small RNA located in IGR between : CDS rocR(>) and CDS CD0442(>)	Putative sRNA candidate		
SQ305	544363	544613	>	* Small RNA located in IGR between : CDS CD0456(<) and CDS CD0457(>)	Putative sRNA candidate		
SQ313	553042	552808	<	* Small RNA located in IGR between : CDS CD0463(<) and CDS CD0464(>)	Putative sRNA candidate		
SQ327	578736	578986	>	* Small RNA located in IGR between : CDS CD0487(<) and CDS sugE(>)	Putative sRNA candidate		
SQ350	631025	631254	>	* Small RNA located in IGR between : CDS CD0524(<) and CDS CD0527(<)	Putative sRNA candidate		
SQ353	638388	638144	<	* Small RNA located in IGR between : CDS CD0532(>) and CDS cheB(>)	Putative sRNA candidate		sCD639
SQ368	655093	654838	<	* Small RNA located in IGR between : CDS CD0549(>) and CDS CD0550(>)	Putative sRNA candidate		
SQ367	654653	654911	>	* Small RNA located in IGR between : CDS CD0549(>) and CDS CD0550(>)	Putative sRNA candidate	NB, RT-PCR	sCD655
SQ390	698574	698344	<	* Small RNA located in IGR between : CDS CD0582(>) and CDS CD0583(<)	Putative sRNA candidate	RT-PCR	
SQ403	750956	751203	>	* Small RNA located in IGR between : CDS CD0627(>) and CDS CD0627A(>)	Putative sRNA candidate		
SQ409	765663	765418	<	* Small RNA located in IGR between : CDS CD0642(>) and CDS CD0643(>)	Putative sRNA candidate		
SQ408	765236	765478	>	* Small RNA located in IGR between : CDS CD0642(>) and CDS CD0643(>)	Putative sRNA candidate		
SQ411	770110	769872	<	* Small RNA located in IGR between : CDS CD0647(>) and CDS CD0648(>)	Putative sRNA candidate		
SQ412	771662	771427	<	* Small RNA located in IGR between : CDS CD0648(>) and CDS CD0649(>)	Putative sRNA candidate		
SQ413	771733	771467	<	* Small RNA located in IGR between : CDS CD0648(>) and CDS CD0649(>)	Putative sRNA candidate		
SQ414	777008	777243	>	* Small RNA located in IGR between : CDS CD0651(>) and CDS CD0652(>)	Putative sRNA candidate		
SQ417	780850	780610	<	* Small RNA located in IGR between : CDS CD0654(>) and CDS CD0655(>)	Putative sRNA candidate		sCD781
SQ416	780428	780665	>	* Small RNA located in IGR between : CDS CD0654(>) and CDS CD0655(>)	Putative sRNA candidate		
SQ420	782470	782217	<	* Small RNA located in IGR between : CDS CD0655(>) and CDS CD0656(>)	Putative sRNA candidate		sCD782
SQ422	785940	786181	>	* Small RNA located in IGR between : CDS cdu1(>) and CDS tcdD(>)	Putative sRNA candidate	RT-PCR	sCD786
SQ426	804279	804037	<	* Small RNA located in IGR between : CDS tcdA(>) and CDS dtxA(<)	Putative sRNA candidate	RT-PCR	sCD804
SQ431	812638	812408	<	* Small RNA located in IGR between : CDS CD0669(>) and CDS CD0670(>)	Putative sRNA candidate		
SQ433	816748	816494	<	* Small RNA located in IGR between : CDS CD0672(>) and CDS CD0673(>)	Putative sRNA candidate		sCD817
SQ437	832423	832662	>	* Small RNA located in IGR between : CDS rplT(>) and CDS CD0688(>)	Putative sRNA candidate		
SQ442	840665	840904	>	* Small RNA located in IGR between : CDS CD0692(<) and CDS CD0693(<)	Putative sRNA candidate		
SQ447	843208	843466	>	* Small RNA located in IGR between : CDS CD0694(>) and CDS CD0695(<)	Putative sRNA candidate		sCD844
SQ453	849549	849317	<	* Small RNA located in IGR between : CDS CD0698(>) and misc_RNA CDs018(>)	Putative sRNA candidate		
SQ486	926103	925868	<	* Small RNA located in IGR between : CDS CD0756(>) and CDS CD0757(>)	Putative sRNA candidate	RT-PCR	sCD926
SQ523	1000248	1000001	<	* Small RNA located in IGR between : CDS CD0824(>) and CDS rbr(>)	Putative sRNA candidate	RT-PCR	
SQ526	1004629	1004390	<	* Small RNA located in IGR between : CDS CD0829(>) and CDS CD0830(>)	Putative sRNA candidate		sCD1005
SQ527	1007609	1007860	>	* Small RNA located in IGR between : CDS CD0831(<) and CDS aksA(>)	Putative sRNA candidate		
SQ528	1012728	1012497	<	* Small RNA located in IGR between : CDS CD0834(>) and CDS CD0835(>)	Putative sRNA candidate		
SQ543	1034056	1034307	>	* Small RNA located in IGR between : CDS oppD(>) and CDS CD0860(>)	Putative sRNA candidate		sCD1034
SQ542	1034053	1034310	>	* Small RNA located in IGR between : CDS oppD(>) and CDS CD0860(>)	Putative sRNA candidate		sCD1034
SQ570	1069987	1069748	<	* Small RNA located in IGR between : CDS CD0887(>) and CDS speA(>)	Putative sRNA candidate		
SQ575	1087534	1087273	<	* Small RNA located in IGR between : CDS CD0902(>) and CDS CD0904(<)	Putative sRNA candidate		
SQ590	1110730	1110488	<	* Small RNA located in IGR between : CDS CD0938(>) and CDS CD0939(>)	Putative sRNA candidate	RT-PCR	sCD1111
SQ606	1124610	1124380	<	* Small RNA located in IGR between : CDS CD0956B(>) and CDS CD0957(>)	Putative sRNA candidate		
SQ608	1126057	1125820	<	* Small RNA located in IGR between : CDS CD0956B(>) and CDS CD0957(>)	Putative sRNA candidate		sCD1125
SQ624	1144698	1144446	<	* Small RNA located in IGR between : CDS CD0979(>) and CDS CD0981(>)	Putative sRNA candidate		
SQ623	1144264	1144513	>	* Small RNA located in IGR between : CDS CD0979(>) and CDS CD0981(>)	Putative sRNA candidate		
SQ636	1162873	1162609	<	* Small RNA located in IGR between : CDS CD0996(>) and CDS CD0997(>)	Putative sRNA candidate		
SQ659	1216722	1216959	>	* Small RNA located in IGR between : CDS CD1035(>) and CDS CD1036(>)	Putative sRNA candidate		sCD1217
SQ666	1238114	1237859	<	* Small RNA located in IGR between : CDS CD1044(>) and CDS CD1045(>)	Putative sRNA candidate		
SQ670	1246479	1246231	<	* Small RNA located in IGR between : CDS CD1053(>) and CDS bcd2(>)	Putative sRNA candidate		sCD1247
SQ675	1253227	1252977	<	* Small RNA located in IGR between : CDS thlA1(>) and CDS CD1060(>)	Putative sRNA candidate		
SQ683	1257140	1256874	<	* Small RNA located in IGR between : CDS CD1063C(>) and CDS ccpA(>)	Putative sRNA candidate		
SQ695	1287443	1287192	<	* Small RNA located in IGR between : CDS CD1094(<) and CDS CD1095(<)	Putative sRNA candidate		sCD1288
SQ726	1322697	1322459	<	* Small RNA located in IGR between : CDS CD1124(>) and CDS CD1124A(>)	Putative sRNA candidate		
SQ729	1323479	1323220	<	* Small RNA located in IGR between : CDS CD1124A(>) and CDS CD1125(>)	Putative sRNA candidate		sCD1324
SQ731	1324502	1324250	<	* Small RNA located in IGR between : CDS CD1125(>) and CDS CD1126(>)	Putative sRNA candidate		
SQ756	1352716	1352454	<	* Small RNA located in IGR between : CDS mgsA(>) and CDS CD1154(>)	Putative sRNA candidate		
SQ754	1352265	1352538	>	* Small RNA located in IGR between : CDS mgsA(>) and CDS CD1154(>)	Putative sRNA candidate		sCD1353
SQ758	1354030	1353779	<	* Small RNA located in IGR between : CDS CD1155(>) and CDS CD1156(>)	Putative sRNA candidate		sCD1354
SQ781	1388944	1388691	<	* Small RNA located in IGR between : CDS CD1186(>) and CDS CD1187(>)	Putative sRNA candidate		sCD1389
SQ810	1434500	1434245	<	* Small RNA located in IGR between : CDS CD1233A(>) and CDS CD1234(>)	Putative sRNA candidate		sCD1435
SQ811	1437975	1437741	<	* Small RNA located in IGR between : CDS CD1234(>) and CDS CD1235(<)	Putative sRNA candidate		
SQ814	1441963	1442204	>	* Small RNA located in IGR between : CDS CD1238(<) and CDS CD1239(>)	Putative sRNA candidate		
SQ829	1461402	1461169	<	* Small RNA located in IGR between : CDS CD1258(>) and CDS CD1259(>)	Putative sRNA candidate		sCD1462
SQ837	1463109	1462858	<	* Small RNA located in IGR between : CDS CD1259(>) and CDS CD1260(>)	Putative sRNA candidate		
SQ836	1463104	1462863	<	* Small RNA located in IGR between : CDS CD1259(>) and CDS CD1260(>)	Putative sRNA candidate		sCD1463
SQ843	1465809	1465552	<	* Small RNA located in IGR between : CDS CD1260(>) and CDS CD1261(>)	Putative sRNA candidate		
SQ842	1465374	1465620	>	* Small RNA located in IGR between : CDS CD1260(>) and CDS CD1261(>)	Putative sRNA candidate		sCD1466
SQ860	1487275	1487007	<	* Small RNA located in IGR between : CDS CD1280(>) and CDS trmU(>)	Putative sRNA candidate		
SQ882	1539922	1539684	<	* Small RNA located in IGR between : CDS pgsA(>) and CDS recA(>)	Putative sRNA candidate		sCD1540
SQ890	1549576	1549331	<	* Small RNA located in IGR between : CDS CD1334(>) and CDS CD1335(>)	Putative sRNA candidate		
SQ901	1572408	1572173	<	* Small RNA located in IGR between : CDS cspB(>) and CDS CD1356(>)	Putative sRNA candidate		sCD1573
SQ910	1616986	1617242	>	* Small RNA located in IGR between : CDS CD1396(<) and CDS CD1397(<)	Putative sRNA candidate		sCD1617
SQ915	1622454	1622699	>	* Small RNA located in IGR between : CDS glpQ(<) and CDS CD1403(>)	Putative sRNA candidate		sCD1623
SQ923	1639252	1639016	<	* Small RNA located in IGR between : CDS CD1413(>) and CDS CD1414(<)	Putative sRNA candidate		
SQ931	1645923	1646169	>	* Small RNA located in IGR between : CDS CD1418B(>) and CDS CD1419(<)	Putative sRNA candidate	NB, RT-PCR	sCD1646
SQ951	1691754	1691511	<	* Small RNA located in IGR between : CDS CD1462(<) and CDS CD1463(>)	Putative sRNA candidate		

SQ950	1691752	1691513	<	* Small RNA located in IGR between : CDS CD1462(<) and CDS CD1463(>)	Putative sRNA candidate		
SQ955	1697912	1697654	<	* Small RNA located in IGR between : CDS CD1467(>) and CDS CD1468(>)	Putative sRNA candidate		
SQ979	1734961	1734701	<	* Small RNA located in IGR between : CDS CD1497(>) and CDS rpoD2(>)	Putative sRNA candidate		
SQ990	1747654	1747894	>	* Small RNA located in IGR between : CDS CD1510(<) and CDS CD1511(<)	Putative sRNA candidate		sCD1748
SQ994	1749685	1749443	<	* Small RNA located in IGR between : CDS CD1511(<) and CDS panC(<)	Putative sRNA candidate		sCD1750
SQ995	1749408	1749672	>	* Small RNA located in IGR between : CDS CD1511(<) and CDS panC(<)	Putative sRNA candidate		
SQ1000	1750231	1749976	<	* Small RNA located in IGR between : CDS CD1511(<) and CDS panC(<)	Putative sRNA candidate		
SQ999	1749794	1750046	>	* Small RNA located in IGR between : CDS CD1511(<) and CDS panC(<)	Putative sRNA candidate		
SQ1001	1752897	1753126	>	* Small RNA located in IGR between : CDS CD1514(<) and CDS CD1515(>)	Putative sRNA candidate		
SQ1002	1761128	1760892	<	* Small RNA located in IGR between : CDS feoA2(<) and CDS CD1519(<)	Putative sRNA candidate	NB, RT-PCR	
SQ1005	1762914	1763156	>	* Small RNA located in IGR between : CDS CD1520(>) and CDS tyrR(>)	Putative sRNA candidate		sCD1763
SQ1021	1786832	1786569	<	* Small RNA located in IGR between : CDS CD1538(>) and CDS CD1539(>)	Putative sRNA candidate		
SQ1031	1802075	1801830	<	* Small RNA located in IGR between : CDS hisI(>) and CDS CD1555(>)	Putative sRNA candidate		
SQ1035	1802356	1802101	<	* Small RNA located in IGR between : CDS hisI(>) and CDS CD1555(>)	Putative sRNA candidate		
SQ1033	1801913	1802177	>	* Small RNA located in IGR between : CDS hisI(>) and CDS CD1555(>)	Putative sRNA candidate		sCD1802
SQ1038	1803950	1804191	>	* Small RNA located in IGR between : CDS CD1555(>) and CDS CD1556(>)	Putative sRNA candidate		
SQ1051	1821341	1821592	>	* Small RNA located in IGR between : CDS tlpB(>) and CDS CD1573(>)	Putative sRNA candidate		
SQ1092	1871335	1871071	<	* Small RNA located in IGR between : CDS CD1615(>) and CDS CD1616(>)	Putative sRNA candidate		sCD1872
SQ1093	1871417	1871144	<	* Small RNA located in IGR between : CDS CD1615(>) and CDS CD1616(>)	Putative sRNA candidate		
SQ1100	1884055	1883796	<	* Small RNA located in IGR between : CDS vanS(>) and CDS vanG(>)	Putative sRNA candidate		
SQ1102	1888419	1888167	<	* Small RNA located in IGR between : CDS vanTG(>) and CDS CD1629(>)	Putative sRNA candidate		sCD1889
SQ1121	1913532	1913291	<	* Small RNA located in IGR between : CDS CD1650(>) and CDS CD1651(>)	Putative sRNA candidate		sCD1914.1
SQ1132	1926956	1926710	<	* Small RNA located in IGR between : CDS gcvPB(>) and CDS CD1659(>)	Putative sRNA candidate		sCD1927
SQ1139	1937760	1937526	<	* Small RNA located in IGR between : CDS guaD(<) and CDS CD1664(<)	Putative sRNA candidate		
SQ1140	1937462	1937702	>	* Small RNA located in IGR between : CDS guaD(<) and CDS CD1664(<)	Putative sRNA candidate		sCD1938
SQ1143	1941830	1941585	<	* Small RNA located in IGR between : CDS CD1666(>) and CDS CD1667(>)	Putative sRNA candidate		
SQ1144	1942434	1942680	>	* Small RNA located in IGR between : CDS CD1667(>) and CDS CD1668(>)	Putative sRNA candidate		
SQ1145	1942549	1942784	>	* Small RNA located in IGR between : CDS CD1667(>) and CDS CD1668(>)	Putative sRNA candidate		
SQ1147	1943616	1943370	<	* Small RNA located in IGR between : CDS CD1667(>) and CDS CD1668(>)	Putative sRNA candidate		
SQ1154	1964735	1964485	<	* Small RNA located in IGR between : CDS trxB1(>) and CDS CD1692(>)	Putative sRNA candidate		sCD1965
SQ1170	1983224	1982982	<	* Small RNA located in IGR between : CDS CD1707(>) and CDS CD1708(>)	Putative sRNA candidate		sCD1983
SQ1180	1997955	1998193	>	* Small RNA located in IGR between : CDS CD1723(<) and CDS CD1724(<)	Putative sRNA candidate		sCD1999
SQ1183	2000255	2000011	<	* Small RNA located in IGR between : CDS CD1726(>) and CDS CD1727(>)	Putative sRNA candidate		
SQ1186	2005995	2005765	<	* Small RNA located in IGR between : CDS CD1730(<) and CDS CD1731(>)	Putative sRNA candidate		
SQ1191	2015957	2015715	<	* Small RNA located in IGR between : CDS CD1739(>) and CDS grdG(>)	Putative sRNA candidate		sCD2016
SQ1194	2018911	2018654	<	* Small RNA located in IGR between : CDS grdF(>) and CDS CD1743(>)	Putative sRNA candidate		sCD2019
SQ1195	2021539	2021769	>	* Small RNA located in IGR between : CDS CD1745(>) and CDS CD1745A(<)	Putative sRNA candidate		
SQ1197	2021574	2021820	>	* Small RNA located in IGR between : CDS CD1745(>) and CDS CD1745A(<)	Putative sRNA candidate		
SQ1204	2028219	2027977	<	* Small RNA located in IGR between : CDS hgdC(>) and CDS CD1751(>)	Putative sRNA candidate		
SQ1208	2032144	2031882	<	* Small RNA located in IGR between : CDS CD1752(>) and CDS CD1753(>)	Putative sRNA candidate		
SQ1225	2054031	2053785	<	* Small RNA located in IGR between : CDS CD1772(>) and CDS CD1773(>)	Putative sRNA candidate		
SQ1251	2101253	2101001	<	* Small RNA located in IGR between : CDS ispH(>) and CDS CD1819(>)	Putative sRNA candidate		
SQ1258	2113269	2113531	>	* Small RNA located in IGR between : CDS metA(>) and CDS CD1827(>)	Putative sRNA candidate	RT-PCR	sCD2114
SQ1271	2136136	2135901	<	* Small RNA located in IGR between : CDS CD1843(<) and CDS CD1844A(>)	Putative sRNA candidate		sCD2136
SQ1273	2136217	2135973	<	* Small RNA located in IGR between : CDS CD1843(<) and CDS CD1844A(>)	Putative sRNA candidate		
SQ1296	2187027	2186776	<	* Small RNA located in IGR between : CDS CD1880(>) and CDS CD1882(<)	Putative sRNA candidate		
SQ1301	2197287	2197027	<	* Small RNA located in IGR between : CDS CD1890(>) and CDS CD1892(>)	Putative sRNA candidate		
SQ1300	2196845	2197102	>	* Small RNA located in IGR between : CDS CD1890(>) and CDS CD1892(>)	Putative sRNA candidate		
SQ1307	2199695	2199459	<	* Small RNA located in IGR between : CDS CD1894(>) and CDS CD1895(>)	Putative sRNA candidate		
SQ1327	2241110	2240851	<	* Small RNA located in IGR between : CDS CD1943(<) and CDS CD1944(<)	Putative sRNA candidate		sCD2242
SQ1326	2240669	2240925	>	* Small RNA located in IGR between : CDS CD1943(<) and CDS CD1944(<)	Putative sRNA candidate		sCD2241
SQ1332	2248341	2248111	<	* Small RNA located in IGR between : CDS CD1949(<) and CDS CD1950(>)	Putative sRNA candidate		
SQ1339	2257886	2257622	<	* Small RNA located in IGR between : CDS CD1957(<) and CDS CD1958(>)	Putative sRNA candidate		
SQ1338	2257440	2257701	>	* Small RNA located in IGR between : CDS CD1957(<) and CDS CD1958(>)	Putative sRNA candidate		
SQ1363	2293426	2293679	>	* Small RNA located in IGR between : CDS CD1987(<) and CDS trpP(<)	Putative sRNA candidate		sCD2294
SQ1369	2294983	2294722	<	* Small RNA located in IGR between : misc_RNA CDs044(<) and CDS CD1989(<)	Putative sRNA candidate		
SQ1371	2295603	2295837	>	* Small RNA located in IGR between : CDS CD1989(<) and CDS CD1990(<)	Putative sRNA candidate		sCD2296
SQ1379	2302998	2302756	<	* Small RNA located in IGR between : CDS CD1994A(<) and CDS CD1995(<)	Putative sRNA candidate		sCD2304
SQ1380	2303245	2303001	<	* Small RNA located in IGR between : CDS CD1994A(<) and CDS CD1995(<)	Putative sRNA candidate		
SQ1422	2321392	2321155	<	* Small RNA located in IGR between : CDS CD2010B(<) and CDS hydD(<)	Putative sRNA candidate		
SQ1426	2328223	2328451	>	* Small RNA located in IGR between : CDS CD2017(>) and CDS CD2018(<)	Putative sRNA candidate		
SQ1445	2354130	2353894	<	* Small RNA located in IGR between : CDS CD2040(>) and CDS CD2041(>)	Putative sRNA candidate		
SQ1447	2355722	2355447	<	* Small RNA located in IGR between : CDS CD2042(<) and CDS CD2043(>)	Putative sRNA candidate		
SQ1452	2359486	2359243	<	* Small RNA located in IGR between : CDS CD2046(<) and CDS CD2047(<)	Putative sRNA candidate	RT-PCR	
SQ1453	2361115	2361356	>	* Small RNA located in IGR between : CDS CD2047(<) and CDS CD2048(<)	Putative sRNA candidate		
SQ1465	2377580	2377335	<	* Small RNA located in IGR between : CDS CD2061(<) and CDS CD2062(<)	Putative sRNA candidate		sCD2378
SQ1484	2415991	2416221	>	* Small RNA located in IGR between : CDS CD2091(<) and CDS CD2092(<)	Putative sRNA candidate		sCD2417
SQ1485	2416050	2416289	>	* Small RNA located in IGR between : CDS CD2091(<) and CDS CD2092(<)	Putative sRNA candidate		
SQ1498	2442021	2441771	<	* Small RNA located in IGR between : CDS CD2112(<) and CDS CD2113(<)	Putative sRNA candidate	NB, RT-PCR	sCD2442
SQ1499	2446631	2446890	>	* Small RNA located in IGR between : CDS CD2115(<) and CDS bipA(>)	Putative sRNA candidate		
SQ1508	2463185	2462947	<	* Small RNA located in IGR between : CDS CD2126(>) and CDS CD2127(<)	Putative sRNA candidate		sCD2464
SQ1509	2463049	2463288	>	* Small RNA located in IGR between : CDS CD2126(>) and CDS CD2127(<)	Putative sRNA candidate		sCD2463
SQ1517	2471694	2471963	>	* Small RNA located in IGR between : CDS CD2134(<) and CDS cdsA(<)	Putative sRNA candidate	RT-PCR	sCD2472
SQ1535	2483356	2483092	<	* Small RNA located in IGR between : CDS CD2145(<) and CDS CD2146(<)	Putative sRNA candidate		
SQ1541	2490216	2490463	>	* Small RNA located in IGR between : CDS CD2151(<) and CDS CD2152(>)	Putative sRNA candidate		
SQ1543	2491702	2491936	>	* Small RNA located in IGR between : CDS CD2152(>) and CDS CD2153(>)	Putative sRNA candidate		
SQ1552	2512842	2513102	>	* Small RNA located in IGR between : CDS CD2171(<) and CDS CD2172(<)	Putative sRNA candidate		sCD2514
SQ1559	2527960	2528218	>	* Small RNA located in IGR between : CDS csdA(<) and CDS CD2184(<)	Putative sRNA candidate		sCD2529
SQ1564	2537555	2537280	<	* Small RNA located in IGR between : CDS CD2190(<) and CDS CD2191(<)	Putative sRNA candidate		sCD2538
SQ1566	2550739	2550968	>	* Small RNA located in IGR between : CDS CD2201(<) and CDS CD2202(>)	Putative sRNA candidate		
SQ1572	2560514	2560270	<	* Small RNA located in IGR between : CDS CD2209(<) and CDS CD2210(<)	Putative sRNA candidate		
SQ1586	2577434	2577187	<	* Small RNA located in IGR between : CDS sat(>) and CDS CD2227(<)	Putative sRNA candidate		sCD2578.1
SQ1587	2577156	2577411	>	* Small RNA located in IGR between : CDS sat(>) and CDS CD2227(<)	Putative sRNA candidate		sCD2578
SQ1605	2620606	2620858	>	* Small RNA located in IGR between : CDS CD2261(<) and CDS CD2262(<)	Putative sRNA candidate		
SQ1631	2660178	2659938	<	* Small RNA located in IGR between : CDS CD2293(>) and CDS CD2294(>)	Putative sRNA candidate		
SQ1638	2664321	2664082	<	* Small RNA located in IGR between : CDS CD2298(<) and CDS CD2299A(<)	Putative sRNA candidate		sCD2665
SQ1642	2665366	2665614	>	* Small RNA located in IGR between : CDS CD2299A(<) and CDS CD2300(<)	Putative sRNA candidate		
SQ1648	2670013	2669774	<	* Small RNA located in IGR between : CDS CD2305(>) and CDS CD2305A(<)	Putative sRNA candidate		sCD2670
SQ1656	2671694	2671929	>	* Small RNA located in IGR between : CDS CD2308(>) and CDS CD2309(>)	Putative sRNA candidate		
SQ1658	2673095	2672854	<	* Small RNA located in IGR between : CDS cspD(>) and CDS CD2311(>)	Putative sRNA candidate		
SQ1670	2704454	2704707	>	* Small RNA located in IGR between : CDS CD2337(<) and CDS abfH(<)	Putative sRNA candidate		
SQ1692	2756807	2756554	<	* Small RNA located in IGR between : CDS CD2386(<) and CDS CD2387(>)	Putative sRNA candidate		
SQ1691	2756372	2756622	>	* Small RNA located in IGR between : CDS CD2386(<) and CDS CD2387(>)	Putative sRNA candidate		
SQ1696	2765084	2765325	>	* Small RNA located in IGR between : CDS CD2395(<) and CDS CD2396(<)	Putative sRNA candidate		sCD2766
SQ1730	2832982	2832724	<	* Small RNA located in IGR between : CDS CD2455(<) and CDS CD2456(>)	Putative sRNA candidate		

SQ1828	2996813	2996569	<	* Small RNA located in IGR between : CDS dapF(<) and CDS CD2478(>)	Putative sRNA candidate		
SQ1834	3004454	3004215	<	* Small RNA located in IGR between : CDS pyrR(<) and CDS CD2596(<)	Putative sRNA candidate		sCD3005
SQ1836	3011885	3012117	>	* Small RNA located in IGR between : CDS CD2602(<) and CDS CD2603(>)	Putative sRNA candidate		
SQ1839	3015475	3015711	>	* Small RNA located in IGR between : CDS CD2603(>) and CDS trpS(<)	Putative sRNA candidate		
SQ1840	3015602	3015834	>	* Small RNA located in IGR between : CDS CD2603(>) and CDS trpS(<)	Putative sRNA candidate		sCD3016
SQ1851	3033126	3033374	>	* Small RNA located in IGR between : CDS CD2625(>) and CDS CD2626(<)	Putative sRNA candidate		
SQ1866	3067399	3067144	<	* Small RNA located in IGR between : CDS CD2657(<) and CDS mraW(<)	Putative sRNA candidate		
SQ1870	3073799	3074038	>	* Small RNA located in IGR between : CDS CD2662(>) and CDS CD2663(<)	Putative sRNA candidate		
SQ1879	3090841	3090598	<	* Small RNA located in IGR between : CDS appC(>) and CDS CD2675(<)	Putative sRNA candidate		
SQ1887	3107184	3106921	<	* Small RNA located in IGR between : CDS CD2687(>) and CDS sspA(<)	Putative sRNA candidate		sCD3107.1
SQ1896	3116386	3116641	>	* Small RNA located in IGR between : CDS CD2696(<) and CDS CD2697(<)	Putative sRNA candidate		
SQ1900	3119501	3119249	<	* Small RNA located in IGR between : CDS CD2699(<) and CDS CD2700(>)	Putative sRNA candidate		
SQ1899	3119067	3119316	>	* Small RNA located in IGR between : CDS CD2699(<) and CDS CD2700(>)	Putative sRNA candidate		
SQ1905	3127271	3127018	<	* Small RNA located in IGR between : CDS CD2704(>) and CDS CD2705(>)	Putative sRNA candidate		
SQ1916	3147677	3147909	>	* Small RNA located in IGR between : CDS CD2717(<) and misc_RNA CDs057(<)	Putative sRNA candidate		
SQ1955	3201384	3201623	>	* Small RNA located in IGR between : CDS CD2754(<) and CDS ptsI(<)	Putative sRNA candidate		
SQ1961	3217625	3217866	>	* Small RNA located in IGR between : CDS CD2767(<) and CDS CD2768(<)	Putative sRNA candidate		sCD3218
SQ1985	3249655	3249888	>	* Small RNA located in IGR between : CDS CD2791(<) and CDS secA2(<)	Putative sRNA candidate	NB, RT-PCR	sCD3250
SQ1999	3266839	3267087	>	* Small RNA located in IGR between : CDS CD2797(<) and CDS CD2798(<)	Putative sRNA candidate		sCD3268
SQ2015	3297440	3297677	>	* Small RNA located in IGR between : CDS CD2824(<) and CDS CD2825(<)	Putative sRNA candidate		sCD3298
SQ2025	3306893	3306644	<	* Small RNA located in IGR between : CDS CD2831(<) and CDS CD2832(<)	Putative sRNA candidate	NB, RT-PCR	sCD3307
SQ2036	3349169	3349421	>	* Small RNA located in IGR between : CDS CD2863(<) and CDS CD2864(<)	Putative sRNA candidate		
SQ2063	3398031	3398258	>	* Small RNA located in IGR between : CDS CD2907(<) and CDS CD2907A(<)	Putative sRNA candidate		sCD3398
SQ2079	3411911	3412150	>	* Small RNA located in IGR between : CDS CD2926(<) and CDS CD2927(<)	Putative sRNA candidate		
SQ2129	3515694	3515937	>	* Small RNA located in IGR between : CDS CD3026(<) and CDS crr(<)	Putative sRNA candidate	RT-PCR	sCD3516
SQ2139	3521234	3521475	>	* Small RNA located in IGR between : CDS CD3031(<) and CDS CD3032(>)	Putative sRNA candidate		sCD3522
SQ2150	3525884	3525645	<	* Small RNA located in IGR between : CDS CD3035(>) and CDS CD3036(<)	Putative sRNA candidate		
SQ2152	3528446	3528212	<	* Small RNA located in IGR between : CDS CD3036(<) and CDS CD3037(<)	Putative sRNA candidate		
SQ2155	3529000	3528754	<	* Small RNA located in IGR between : CDS CD3036(<) and CDS CD3037(<)	Putative sRNA candidate		sCD3529
SQ2153	3528569	3528818	>	* Small RNA located in IGR between : CDS CD3036(<) and CDS CD3037(<)	Putative sRNA candidate		
SQ2159	3528833	3529066	>	* Small RNA located in IGR between : CDS CD3036(<) and CDS CD3037(<)	Putative sRNA candidate		
SQ2163	3534423	3534664	>	* Small RNA located in IGR between : CDS pepI(<) and CDS CD3042(>)	Putative sRNA candidate		
SQ2182	3570148	3569880	<	* Small RNA located in IGR between : CDS CD3073(>) and CDS gatY(<)	Putative sRNA candidate		sCD3570.1
SQ2189	3574844	3575095	>	* Small RNA located in IGR between : CDS CD3077(<) and CDS CD3078(<)	Putative sRNA candidate		
SQ2224	3637027	3636785	<	* Small RNA located in IGR between : CDS CD3123(<) and CDS ascB2(<)	Putative sRNA candidate		sCD3637
SQ2227	3651453	3651709	>	* Small RNA located in IGR between : CDS CD3133(<) and CDS CD3134(<)	Putative sRNA candidate		
SQ2233	3654956	3655194	>	* Small RNA located in IGR between : CDS CD3135(<) and CDS bglA3(<)	Putative sRNA candidate		sCD3656
SQ2245	3664650	3664910	>	* Small RNA located in IGR between : CDS CD3144(<) and CDS CD3145(<)	Putative sRNA candidate		sCD3665
SQ2264	3700643	3700395	<	* Small RNA located in IGR between : CDS CD3167(>) and CDS CD3168(>)	Putative sRNA candidate		sCD3701
SQ2267	3702359	3702628	>	* Small RNA located in IGR between : CDS CD3169(>) and CDS secG(<)	Putative sRNA candidate		sCD3703
SQ2273	3704998	3705235	>	* Small RNA located in IGR between : CDS eno(<) and CDS gpmI(<)	Putative sRNA candidate		
SQ2280	3726836	3726585	<	* Small RNA located in IGR between : CDS dpaL2(<) and CDS CD3186(<)	Putative sRNA candidate		
SQ2281	3734142	3734403	>	* Small RNA located in IGR between : CDS CD3191(<) and CDS CD3192(>)	Putative sRNA candidate		
SQ2284	3736437	3736187	<	* Small RNA located in IGR between : CDS CD3192(>) and CDS CD3193(<)	Putative sRNA candidate	RT-PCR	
SQ2286	3738023	3737782	<	* Small RNA located in IGR between : CDS CD3193(<) and CDS CD3194(>)	Putative sRNA candidate		
SQ2289	3745231	3744971	<	* Small RNA located in IGR between : CDS cme(>) and CDS CD3199(<)	Putative sRNA candidate		sCD3745
SQ2303	3767027	3766769	<	* Small RNA located in IGR between : CDS CD3218(<) and CDS hslO(<)	Putative sRNA candidate		sCD3767
SQ2322	3784853	3784603	<	* Small RNA located in IGR between : CDS CD3232(<) and CDS CD3233(<)	Putative sRNA candidate		sCD3785
SQ2347	3809820	3810072	>	* Small RNA located in IGR between : CDS CD3254(<) and CDS CD3255(<)	Putative sRNA candidate		
SQ2363	3841098	3840839	<	* Small RNA located in IGR between : CDS CD3280(<) and CDS proC2(<)	Putative sRNA candidate		sCD3841
SQ2396	3893953	3893703	<	* Small RNA located in IGR between : CDS CD3329(<) and CDS CD3330(>)	Putative sRNA candidate		
SQ2397	3894182	3893938	<	* Small RNA located in IGR between : CDS CD3329(<) and CDS CD3330(>)	Putative sRNA candidate		
SQ2428	3931366	3931596	>	* Small RNA located in IGR between : CDS CD3365(>) and CDS CD3366(<)	Putative sRNA candidate		
SQ2429	3936132	3936367	>	* Small RNA located in IGR between : CDS CD3368A(<) and CDS CD3369(>)	Putative sRNA candidate		
SQ2480	4045015	4044764	<	* Small RNA located in IGR between : CDS lacC(<) and CDS CD3452(>)	Putative sRNA candidate		
SQ2484	4048471	4048217	<	* Small RNA located in IGR between : CDS CD3454(<) and CDS CD3455(<)	Putative sRNA candidate		
SQ2485	4048762	4048514	<	* Small RNA located in IGR between : CDS CD3454(<) and CDS CD3455(<)	Putative sRNA candidate		sCD4049
SQ2503	4079680	4079425	<	* Small RNA located in IGR between : CDS CD3489(<) and CDS spoIIE(<)	Putative sRNA candidate	NB, RT-PCR	sCD4080
SQ2504	4079472	4079703	>	* Small RNA located in IGR between : CDS CD3489(<) and CDS spoIIE(<)	Putative sRNA candidate	RT-PCR	
SQ2524	4111000	4110763	<	* Small RNA located in IGR between : CDS purR(<) and CDS murC(>)	Putative sRNA candidate		
SQ2534	4122480	4122721	>	* Small RNA located in IGR between : CDS CD3527(<) and CDS CD3528(<)	Putative sRNA candidate		sCD4123
SQ2562	4178651	4178415	<	* Small RNA located in IGR between : CDS mdeA(<) and CDS CD3578(<)	Putative sRNA candidate		sCD4179
SQ2564	4178748	4178487	<	* Small RNA located in IGR between : CDS mdeA(<) and CDS CD3578(<)	Putative sRNA candidate		
SQ2563	4178305	4178563	>	* Small RNA located in IGR between : CDS mdeA(<) and CDS CD3578(<)	Putative sRNA candidate		
SQ2570	4199162	4198923	<	* Small RNA located in IGR between : CDS pyrF(<) and CDS CD3593(<)	Putative sRNA candidate		
SQ2582	4217770	4217519	<	* Small RNA located in IGR between : CDS CD3610(<) and CDS CD3613(>)	Putative sRNA candidate		
SQ2584	4223875	4223641	<	* Small RNA located in IGR between : CDS CD3616(<) and CDS CD3617(<)	Putative sRNA candidate		
SQ2589	4231816	4231553	<	* Small RNA located in IGR between : CDS CD3624(<) and CDS CD3625(<)	Putative sRNA candidate		
SQ2596	4241162	4240925	<	* Small RNA located in IGR between : CDS CD3631(<) and CDS CD3632(<)	Putative sRNA candidate		sCD4266

Name	5'_start	3'_end	Strand	Annotation1	Annotation2	Validation
SQ27	45556	45324	<	==> Putative antisense to 3' UTR of CDS : clpC(>)	Putative sRNA candidate	
SQ39	71687	71431	<	==> Putative antisense to 3' UTR of CDS : CD0046(>)	Putative sRNA candidate	
SQ48	78708	78469	<	==> Putative antisense to 3' UTR of CDS : gltX(>)	Putative sRNA candidate	
SQ53	88738	88504	<	==> Putative antisense to 3' UTR of CDS : rplL(>)	Putative sRNA candidate	
SQ85	141180	140923	<	==> Putative antisense to 3' UTR of CDS : nrdG(>)	Putative sRNA candidate	
SQ105	161848	161607	<	==> Putative antisense to 3' UTR of CDS : CD0118(>)	Putative sRNA candidate	
SQ108	171891	171657	<	==> Putative antisense to 3' UTR of CDS : spoIIID(>)	Putative sRNA candidate	
SQ138	230732	230499	<	==> Putative antisense to 3' UTR of CDS : CD0173(>)	Putative sRNA candidate	
SQ146	239068	238838	<	==> Putative antisense to 3' UTR of CDS : CD0180(>)	Putative sRNA candidate	RT-PCR
SQ183	333544	333308	<	==> Putative antisense to 3' UTR of CDS : CD0272(>)	Putative sRNA candidate	
SQ295	525956	525719	<	==> Putative antisense to 3' UTR of CDS : rocR(>)	Putative sRNA candidate	
SQ359	648205	647959	<	==> Putative antisense to 3' UTR of CDS : CD0542(>)	Putative sRNA candidate	
SQ533	1020312	1020077	<	==> Putative antisense to 3' UTR of CDS : CD0843(>)	Putative sRNA candidate	
SQ569	1069641	1069401	<	==> Putative antisense to 3' UTR of CDS : CD0887(>)	Putative sRNA candidate	
SQ583	1097907	1097672	<	==> Putative antisense to 3' UTR of CDS : CD0915(>)	Putative sRNA candidate	
SQ587	1109755	1109520	<	==> Putative antisense to 3' UTR of CDS : CD0937(>)	Putative sRNA candidate	
SQ617	1139478	1139231	<	==> Putative antisense to 3' UTR of CDS : CD0973(>)	Putative sRNA candidate	RT-PCR
SQ682	1257126	1256888	<	==> Putative antisense to 3' UTR of CDS : CD1063C(>)	Putative sRNA candidate	
SQ685	1258391	1258147	<	==> Putative antisense to 3' UTR of CDS : ccpA(>)	Putative sRNA candidate	
SQ723	1320127	1319892	<	==> Putative antisense to 3' UTR of CDS : CD1122(>)	Putative sRNA candidate	
SQ764	1362394	1362159	<	==> Putative antisense to 3' UTR of CDS : rpmA(>)	Putative sRNA candidate	
SQ774	1377711	1377472	<	==> Putative antisense to 3' UTR of CDS : rpmF(>)	Putative sRNA candidate	
SQ808	1434312	1434082	<	==> Putative antisense to 3' UTR of CDS : CD1233A(>)	Putative sRNA candidate	
SQ840	1465430	1465200	<	==> Putative antisense to 3' UTR of CDS : CD1260(>)	Putative sRNA candidate	
SQ845	1470460	1470217	<	==> Putative antisense to 3' UTR of CDS : CD1264(>)	Putative sRNA candidate	
SQ850	1474150	1474378	>	==> Putative antisense to 3' UTR of CDS : CD1269(<)	Putative sRNA candidate	RT-PCR
SQ874	1523538	1523306	<	==> Putative antisense to 3' UTR of CDS : tlpB(>)	Putative sRNA candidate	
SQ877	1526313	1526075	<	==> Putative antisense to 3' UTR of CDS : rpsO(>)	Putative sRNA candidate	
SQ909	1615622	1615365	<	==> Putative antisense to 3' UTR of CDS : CD1394(>)	Putative sRNA candidate	
SQ944	1682859	1682628	<	==> Putative antisense to 3' UTR of CDS : CD1453(>)	Putative sRNA candidate	
SQ978	1734949	1734713	<	==> Putative antisense to 3' UTR of CDS : CD1497(>)	Putative sRNA candidate	
SQ983	1738744	1738512	<	==> Putative antisense to 3' UTR of CDS : tlpB(>)	Putative sRNA candidate	
SQ1043	1811050	1810787	<	==> Putative antisense to 3' UTR of misc_RNA : CDs028(>)	Putative sRNA candidate	
SQ1050	1820954	1820722	<	==> Putative antisense to 3' UTR of CDS : tlpB(>)	Putative sRNA candidate	
SQ1061	1831390	1831144	<	==> Putative antisense to 3' UTR of misc_RNA : CDs030(>)	Putative sRNA candidate	
SQ1076	1851335	1851585	>	==> Putative antisense to 3' UTR of CDS : map2(<)	Putative sRNA candidate	RT-PCR
SQ1157	1965708	1965465	<	==> Putative antisense to 3' UTR of CDS : CD1693(>)	Putative sRNA candidate	
SQ1175	1994912	1994680	<	==> Putative antisense to 3' UTR of CDS : tlpB(>)	Putative sRNA candidate	
SQ1189	2015908	2015672	<	==> Putative antisense to 3' UTR of CDS : CD1739(>)	Putative sRNA candidate	
SQ1200	2022304	2022535	>	==> Putative antisense to 3' UTR of CDS : gltC(<)	Putative sRNA candidate	
SQ1223	2053006	2052756	<	==> Putative antisense to 3' UTR of CDS : CD1771(>)	Putative sRNA candidate	
SQ1241	2080629	2080388	<	==> Putative antisense to 3' UTR of CDS : CD1798(>)	Putative sRNA candidate	
SQ1255	2104873	2104641	<	==> Putative antisense to 3' UTR of CDS : tlpB(>)	Putative sRNA candidate	
SQ1262	2116110	2115877	<	==> Putative antisense to 3' UTR of CDS : ftsH1(>)	Putative sRNA candidate	
SQ1287	2177877	2177634	<	==> Putative antisense to 3' UTR of CDS : vexP3(>)	Putative sRNA candidate	
SQ1298	2192450	2192204	<	==> Putative antisense to 3' UTR of CDS : CD1886(>)	Putative sRNA candidate	
SQ1308	2202501	2202738	>	==> Putative antisense to 3' UTR of CDS : CD1900(<)	Putative sRNA candidate	
SQ1356	2284971	2285215	>	==> Putative antisense to 3' UTR of misc_RNA : CDs043(<)	Putative sRNA candidate	
SQ1376	2300297	2300065	<	==> Putative antisense to 3' UTR of CDS : CD1992(>)	Putative sRNA candidate	
SQ1448	2356523	2356283	<	==> Putative antisense to 3' UTR of CDS : CD2043(>)	Putative sRNA candidate	
SQ1469	2380018	2379782	<	==> Putative antisense to 3' UTR of CDS : CD2064(>)	Putative sRNA candidate	RT-PCR
SQ1532	2481296	2481045	<	==> Putative antisense to 3' UTR of CDS : CD2144(>)	Putative sRNA candidate	
SQ1608	2620673	2620904	>	==> Putative antisense to 3' UTR of CDS : CD2262(<)	Putative sRNA candidate	
SQ1633	2661323	2661074	<	==> Putative antisense to 3' UTR of CDS : CD2295(>)	Putative sRNA candidate	
SQ1635	2662329	2662583	>	==> Putative antisense to 3' UTR of CDS : CD2298(<)	Putative sRNA candidate	
SQ1739	2841564	2841795	>	==> Putative antisense to 3' UTR of CDS : grpE(<)	Putative sRNA candidate	
SQ1822	2969657	2969417	<	==> Putative antisense to 3' UTR of CDS : CD2567(>)	Putative sRNA candidate	
SQ1888	3107145	3107385	>	==> Putative antisense to 3' UTR of CDS : sspA(<)	Putative sRNA candidate	
SQ1929	3169424	3169160	<	==> Putative antisense to 3' UTR of CDS : CD2732(>)	Putative sRNA candidate	
SQ1981	3245177	3244936	<	==> Putative antisense to 3' UTR of CDS : CD2788(>)	Putative sRNA candidate	
SQ2083	3412369	3412603	>	==> Putative antisense to 3' UTR of CDS : CD2927(<)	Putative sRNA candidate	
SQ2086	3415215	3415447	>	==> Putative antisense to 3' UTR of CDS : CD2930(<)	Putative sRNA candidate	
SQ2090	3424176	3424409	>	==> Putative antisense to 3' UTR of CDS : CD2946(<)	Putative sRNA candidate	
SQ2179	3563188	3562943	<	==> Putative antisense to 3' UTR of CDS : xylR(>)	Putative sRNA candidate	
SQ2198	3592341	3592104	<	==> Putative antisense to 3' UTR of CDS : treR(>)	Putative sRNA candidate	
SQ2206	3604800	3605039	>	==> Putative antisense to 3' UTR of CDS : CD3099(<)	Putative sRNA candidate	
SQ2209	3607983	3608225	>	==> Putative antisense to 3' UTR of CDS : CD3101(<)	Putative sRNA candidate	
SQ2211	3611332	3611566	>	==> Putative antisense to 3' UTR of CDS : CD3103(<)	Putative sRNA candidate	
SQ2215	3616772	3616525	<	==> Putative antisense to 3' UTR of CDS : CD3107(>)	Putative sRNA candidate	
SQ2308	3773470	3773230	<	==> Putative antisense to 3' UTR of misc_RNA : CDs061(>)	Putative sRNA candidate	
SQ2366	3845641	3845885	>	==> Putative antisense to 3' UTR of CDS : CD3284(<)	Putative sRNA candidate	
SQ2365	3845639	3845887	>	==> Putative antisense to 3' UTR of CDS : CD3284(<)	Putative sRNA candidate	
SQ2476	4039961	4040208	>	==> Putative antisense to 3' UTR of CDS : gatY(<)	Putative sRNA candidate	
SQ2479	4043894	4043649	<	==> Putative antisense to 3' UTR of CDS : CD3450(>)	Putative sRNA candidate	
SQ2523	4109571	4109799	>	==> Putative antisense to 3' UTR of CDS : purR(<)	Putative sRNA candidate	
SQ2540	4146385	4146629	>	==> Putative antisense to 3' UTR of CDS : CD3551A(<)	Putative sRNA candidate	
SQ2585	4223590	4223831	>	==> Putative antisense to 3' UTR of CDS : CD3617(<)	Putative sRNA candidate	
SQ2591	4234503	4234766	>	==> Putative antisense to 3' UTR of CDS : kdgA(<)	Putative sRNA candidate	
SQ2610	4257716	4257954	>	==> Putative antisense to 3' UTR of CDS : CD3649(<)	Putative sRNA candidate	

Name	5'_start	3'_end	Strand	Annotation1	Annotation2
SQ4	1647	1399	<	==> Putative antisense to 5' UTR of CDS : dnaN(>)	Putative sRNA candidate
SQ22	34298	34059	<	==> Putative antisense to 5' UTR of CDS : CD0020(>)	==> Putative antisense to 3' UTR of tRNA : tRNA-Val(>)
SQ43	73432	73181	<	==> Putative antisense to 5' UTR of CDS : proS(>)	==> Putative antisense to 3' UTR of misc_RNA : CDs003(>)
SQ61	101093	100842	<	==> Putative antisense to 5' UTR of CDS : tufB(>)	Putative sRNA candidate
SQ60	101090	100845	<	==> Putative antisense to 5' UTR of CDS : tufB(>)	Putative sRNA candidate
SQ382	692642	692892	>	==> Putative antisense to 5' UTR of CDS : CD0579(<)	Putative sRNA candidate
SQ477	904111	903880	<	==> Putative antisense to 5' UTR of CDS : glpK1(>)	Putative sRNA candidate
SQ582	1096460	1096213	<	==> Putative antisense to 5' UTR of CDS : CD0911(>)	Putative sRNA candidate
SQ593	1112780	1112543	<	==> Putative antisense to 5' UTR of CDS : CD0943(>)	==> Putative antisense to 3' UTR of CDS : CD0942(>)
SQ671	1251011	1250742	<	==> Putative antisense to 5' UTR of CDS : hbd(>)	Putative sRNA candidate
SQ673	1251929	1251697	<	==> Putative antisense to 5' UTR of CDS : thlA1(>)	==> Putative antisense to 3' UTR of CDS : hbd(>)
SQ733	1332352	1332120	<	==> Putative antisense to 5' UTR of CDS : CD1132(>)	Putative sRNA candidate
SQ817	1443216	1442951	<	==> Putative antisense to 5' UTR of CDS : vanZ(>)	Putative sRNA candidate
SQ1048	1815424	1815176	<	==> Putative antisense to 5' UTR of CDS : CD1568(>)	Putative sRNA candidate
SQ1087	1868376	1868121	<	==> Putative antisense to 5' UTR of CDS : CD1614(>)	Putative sRNA candidate
SQ1096	1873634	1873389	<	==> Putative antisense to 5' UTR of CDS : CD1617(>)	Putative sRNA candidate
SQ1113	1895870	1895626	<	==> Putative antisense to 5' UTR of CDS : CD1636(>)	Putative sRNA candidate
SQ1162	1972945	1973179	>	==> Putative antisense to 5' UTR of CDS : ribD(<)	Putative sRNA candidate
SQ1423	2322973	2323206	>	==> Putative antisense to 5' UTR of CDS : hydR(<)	Putative sRNA candidate
SQ1573	2565002	2565234	>	==> Putative antisense to 5' UTR of CDS : CD2212(<)	Putative sRNA candidate
SQ1653	2671248	2670994	<	==> Putative antisense to 5' UTR of CDS : CD2307(>)	Putative sRNA candidate
SQ1736	2839473	2839720	>	==> Putative antisense to 5' UTR of CDS : dnaJ(<)	Putative sRNA candidate
SQ1883	3102053	3102290	>	==> Putative antisense to 5' UTR of CDS : nifJ(<)	Putative sRNA candidate
SQ2075	3409848	3410108	>	==> Putative antisense to 5' UTR of CDS : CD2923(<)	==> Putative antisense to 3' UTR of CDS : CD2924(<)
SQ2145	3524190	3523947	<	==> Putative antisense to 5' UTR of CDS : CD3034(>)	Putative sRNA candidate
SQ2244	3664005	3664242	>	==> Putative antisense to 5' UTR of CDS : CD3144(<)	Putative sRNA candidate
SQ2330	3792434	3792686	>	==> Putative antisense to 5' UTR of CDS : CD3240(<)	Putative sRNA candidate
SQ2607	4256701	4256947	>	==> Putative antisense to 5' UTR of CDS : CD3646(<)	Putative sRNA candidate

Name	5'_start	3'_end	Strand	Annotation1	Annotation2	Validation
SQ8	10080	9847	<	==> Putative antisense to CDS : sigB(>)	==> Putative antisense to 3' UTR of CDS : rsbW(>)	
SQ10	13316	13077	<	==> Putative antisense to rRNA : 23s_rRNA(>)	Putative sRNA candidate	
SQ19	27528	27289	<	==> Putative antisense to rRNA : 23s_rRNA(>)	Putative sRNA candidate	
SQ20	27692	27459	<	==> Putative antisense to rRNA : 23s_rRNA(>)	Putative sRNA candidate	
SQ36	64043	63809	<	==> Putative antisense to CDS : CD0040(>)	Putative sRNA candidate	
SQ44	73534	73299	<	==> Putative antisense to CDS : proS(>)	Putative sRNA candidate	
SQ46	73885	73651	<	==> Putative antisense to CDS : proS(>)	Putative sRNA candidate	
SQ58	91256	91026	<	==> Putative antisense to CDS : rpoB(>)	Putative sRNA candidate	
SQ59	93107	92871	<	==> Putative antisense to CDS : rpoB(>)	Putative sRNA candidate	
SQ65	111161	110921	<	==> Putative antisense to CDS : rpsE(>)	Putative sRNA candidate	
SQ67	117033	116792	<	==> Putative antisense to CDS : rpsD(>)	Putative sRNA candidate	RT-PCR
SQ73	126655	126416	<	==> Putative antisense to rRNA : 23s_rRNA(>)	Putative sRNA candidate	
SQ74	126819	126586	<	==> Putative antisense to rRNA : 23s_rRNA(>)	Putative sRNA candidate	
SQ76	133602	133363	<	==> Putative antisense to rRNA : 23s_rRNA(>)	Putative sRNA candidate	
SQ77	133766	133533	<	==> Putative antisense to rRNA : 23s_rRNA(>)	Putative sRNA candidate	
SQ87	144151	143912	<	==> Putative antisense to rRNA : 23s_rRNA(>)	Putative sRNA candidate	
SQ88	144315	144082	<	==> Putative antisense to rRNA : 23s_rRNA(>)	Putative sRNA candidate	
SQ90	149545	149306	<	==> Putative antisense to rRNA : 23s_rRNA(>)	Putative sRNA candidate	
SQ91	149709	149476	<	==> Putative antisense to rRNA : 23s_rRNA(>)	Putative sRNA candidate	
SQ115	180084	179848	<	==> Putative antisense to CDS : CD0133(>)	Putative sRNA candidate	
SQ124	192691	192461	<	==> Putative antisense to CDS : prfB(>)	Putative sRNA candidate	
SQ137	230361	230131	<	==> Putative antisense to CDS : CD0173(>)	Putative sRNA candidate	
SQ151	243304	243074	<	==> Putative antisense to CDS : pyrB(>)	Putative sRNA candidate	
SQ156	253205	252973	<	==> Putative antisense to CDS : groEL(>)	Putative sRNA candidate	
SQ170	298530	298298	<	==> Putative antisense to CDS : fliS1(>)	Putative sRNA candidate	
SQ177	314593	314350	<	==> Putative antisense to CDS : fliI(>)	Putative sRNA candidate	
SQ178	318367	318125	<	==> Putative antisense to CDS : flgE(>)	Putative sRNA candidate	
SQ184	333822	333590	<	==> Putative antisense to CDS : htpG(>)	Putative sRNA candidate	
SQ218	379560	379321	<	==> Putative antisense to rRNA : 23s_rRNA(>)	Putative sRNA candidate	
SQ219	379724	379491	<	==> Putative antisense to rRNA : 23s_rRNA(>)	Putative sRNA candidate	
SQ225	398837	398601	<	==> Putative antisense to CDS : ppiB(>)	Putative sRNA candidate	
SQ231	401961	402192	>	==> Putative antisense to CDS : ppaC(<)	Putative sRNA candidate	
SQ304	538706	538460	<	==> Putative antisense to CDS : CD0453B(>)	Putative sRNA candidate	
SQ356	646371	646128	<	==> Putative antisense to CDS : CD0540(>)	Putative sRNA candidate	
SQ375	675836	675597	<	==> Putative antisense to CDS : CD0566(>)	Putative sRNA candidate	
SQ378	683611	683370	<	==> Putative antisense to misc_RNA : CDs016(>)	Putative sRNA candidate	
SQ394	721608	721378	<	==> Putative antisense to CDS : cotJC1(>)	Putative sRNA candidate	
SQ423	786895	786645	<	==> Putative antisense to CDS : tcdD(>)	Putative sRNA candidate	RT-PCR
SQ429	809886	809648	<	==> Putative antisense to CDS : CD0668(>)	Putative sRNA candidate	
SQ464	866310	866080	<	==> Putative antisense to CDS : CD0709(>)	Putative sRNA candidate	
SQ465	868119	867888	<	==> Putative antisense to CDS : CD0710(>)	Putative sRNA candidate	
SQ476	902987	903237	>	==> Putative antisense to CDS : CD0740(<)	Putative sRNA candidate	
SQ491	929265	929494	>	==> Putative antisense to CDS : plfB(<)	Putative sRNA candidate	
SQ495	939241	939000	<	==> Putative antisense to CDS : srlB(>)	Putative sRNA candidate	
SQ496	943573	943334	<	==> Putative antisense to CDS : sigF(>)	Putative sRNA candidate	
SQ499	957188	956958	<	==> Putative antisense to CDS : CD0784(>)	Putative sRNA candidate	
SQ557	1057061	1056824	<	==> Putative antisense to CDS : CD0879(>)	Putative sRNA candidate	
SQ561	1063491	1063255	<	==> Putative antisense to CDS : glgA(>)	Putative sRNA candidate	
SQ565	1067277	1067045	<	==> Putative antisense to CDS : CD0886(>)	Putative sRNA candidate	
SQ578	1094952	1094712	<	==> Putative antisense to CDS : CD0908(>)	Putative sRNA candidate	
SQ639	1172565	1172320	<	==> Putative antisense to CDS : fumA(>)	Putative sRNA candidate	
SQ654	1199112	1198876	<	==> Putative antisense to CDS : potD(>)	Putative sRNA candidate	
SQ655	1211598	1211359	<	==> Putative antisense to rRNA : 23s_rRNA(>)	Putative sRNA candidate	
SQ656	1211763	1211530	<	==> Putative antisense to rRNA : 23s_rRNA(>)	Putative sRNA candidate	
SQ662	1227306	1227075	<	==> Putative antisense to CDS : addB(>)	Putative sRNA candidate	
SQ663	1236225	1235985	<	==> Putative antisense to CDS : sbcC(>)	Putative sRNA candidate	
SQ771	1372946	1372711	<	==> Putative antisense to CDS : CD1173(>)	Putative sRNA candidate	
SQ807	1434260	1434020	<	==> Putative antisense to CDS : CD1233A(>)	Putative sRNA candidate	
SQ872	1515319	1515089	<	==> Putative antisense to CDS : dnaF(>)	Putative sRNA candidate	
SQ884	1541453	1541211	<	==> Putative antisense to CDS : CD1329(>)	Putative sRNA candidate	
SQ885	1541950	1541718	<	==> Putative antisense to CDS : CD1329(>)	Putative sRNA candidate	
SQ887	1548334	1548093	<	==> Putative antisense to CDS : CD1334(>)	Putative sRNA candidate	
SQ902	1573557	1573784	>	==> Putative antisense to CDS : CD1357(<)	Putative sRNA candidate	RT-PCR
SQ917	1629395	1629622	>	==> Putative antisense to CDS : CD1405(<)	Putative sRNA candidate	
SQ940	1655799	1656026	>	==> Putative antisense to CDS : CD1428(<)	Putative sRNA candidate	
SQ952	1692985	1692744	<	==> Putative antisense to CDS : CD1464(>)	Putative sRNA candidate	
SQ970	1729268	1729032	<	==> Putative antisense to CDS : CD1492(>)	Putative sRNA candidate	
SQ989	1746882	1746626	<	==> Putative antisense to CDS : CD1509(>)	Putative sRNA candidate	
SQ1019	1783438	1783188	<	==> Putative antisense to CDS : aspB(>)	Putative sRNA candidate	
SQ1037	1802948	1802716	<	==> Putative antisense to CDS : CD1555(>)	Putative sRNA candidate	
SQ1049	1816687	1816452	<	==> Putative antisense to CDS : CD1568(>)	Putative sRNA candidate	
SQ1097	1873739	1873504	<	==> Putative antisense to CDS : CD1617(>)	Putative sRNA candidate	
SQ1114	1898127	1897895	<	==> Putative antisense to CDS : CD1637(>)	Putative sRNA candidate	
SQ1149	1954146	1954400	>	==> Putative antisense to CDS : CD1678A(<)	Putative sRNA candidate	
SQ1179	1997164	1997397	>	==> Putative antisense to CDS : CD1723(<)	Putative sRNA candidate	
SQ1192	2017610	2017380	<	==> Putative antisense to CDS : grdF(>)	Putative sRNA candidate	
SQ1267	2128803	2128572	<	==> Putative antisense to CDS : tyrC(>)	Putative sRNA candidate	
SQ1285	2176866	2176634	<	==> Putative antisense to CDS : vexP3(>)	Putative sRNA candidate	
SQ1289	2178975	2178743	<	==> Putative antisense to CDS : vncS(>)	Putative sRNA candidate	

SQ1290	2179460	2179230	<	==> Putative antisense to CDS : vncS(>)	Putative sRNA candidate	
SQ1299	2192857	2192627	<	==> Putative antisense to CDS : CD1887(>)	Putative sRNA candidate	
SQ1316	2209732	2209500	<	==> Putative antisense to CDS : pduQ(>)	Putative sRNA candidate	
SQ1321	2234319	2234577	>	==> Putative antisense to CDS : accA(<)	Putative sRNA candidate	
SQ1322	2236155	2236384	>	==> Putative antisense to CDS : accC(<)	Putative sRNA candidate	
SQ1366	2294200	2294442	>	==> Putative antisense to misc_RNA : CDs044(<)	Putative sRNA candidate	
SQ1381	2304227	2304454	>	==> Putative antisense to CDS : CD1996(<)	Putative sRNA candidate	
SQ1395	2314179	2314407	>	==> Putative antisense to CDS : effD(<)	Putative sRNA candidate	
SQ1437	2343349	2343578	>	==> Putative antisense to CDS : argM(<)	Putative sRNA candidate	
SQ1464	2376523	2376752	>	==> Putative antisense to CDS : CD2061(<)	Putative sRNA candidate	
SQ1503	2456928	2456694	<	==> Putative antisense to CDS : CD2122(>)	Putative sRNA candidate	
SQ1511	2464709	2464949	>	==> Putative antisense to CDS : ispG(<)	Putative sRNA candidate	
SQ1514	2469450	2469215	<	==> Putative antisense to CDS : CD2133(>)	==> Putative long 3' UTR of CDS : CD2134(<)	
SQ1529	2477137	2477364	>	==> Putative antisense to CDS : CD2141(<)	Putative sRNA candidate	
SQ1549	2503480	2503233	<	==> Putative antisense to CDS : ldh(>)	Putative sRNA candidate	
SQ1625	2657464	2657709	>	==> Putative antisense to CDS : CD2289(<)	Putative sRNA candidate	
SQ1628	2660000	2659760	<	==> Putative antisense to CDS : CD2293(>)	Putative sRNA candidate	
SQ1634	2662061	2662304	>	==> Putative antisense to CDS : CD2297(<)	Putative sRNA candidate	
SQ1641	2665093	2665330	>	==> Putative antisense to CDS : CD2299A(<)	Putative sRNA candidate	
SQ1726	2822597	2822825	>	==> Putative antisense to CDS : CD2448(<)	Putative sRNA candidate	
SQ1741	2846733	2846971	>	==> Putative antisense to CDS : CD2466(<)	Putative sRNA candidate	
SQ1757	2861741	2861968	>	==> Putative antisense to CDS : CD2480(<)	Putative sRNA candidate	
SQ1771	2885929	2886166	>	==> Putative antisense to misc_RNA : CDs051(<)	Putative sRNA candidate	
SQ1772	2886516	2886749	>	==> Putative antisense to CDS : CD2501(<)	Putative sRNA candidate	
SQ1781	2908031	2908258	>	==> Putative antisense to CDS : CD2517A(<)	Putative sRNA candidate	NB, RT-PCR
SQ1783	2911229	2910997	<	==> Putative antisense to CDS : CD2519(>)	Putative sRNA candidate	
SQ1850	3030185	3030418	>	==> Putative antisense to CDS : CD2623(<)	Putative sRNA candidate	
SQ1877	3084977	3085205	>	==> Putative antisense to CDS : appF(<)	Putative sRNA candidate	
SQ1890	3108392	3108623	>	==> Putative antisense to CDS : CD2689(<)	Putative sRNA candidate	
SQ1923	3151625	3151855	>	==> Putative antisense to rRNA : 23s_rRNA(<)	Putative sRNA candidate	
SQ1924	3151789	3152025	>	==> Putative antisense to rRNA : 23s_rRNA(<)	Putative sRNA candidate	
SQ1941	3178155	3178384	>	==> Putative antisense to CDS : aspS(<)	Putative sRNA candidate	
SQ1942	3181035	3181263	>	==> Putative antisense to CDS : CD2741(<)	Putative sRNA candidate	
SQ1946	3185907	3186138	>	==> Putative antisense to CDS : apt(<)	Putative sRNA candidate	
SQ1960	3215965	3216199	>	==> Putative antisense to CDS : CD2767(<)	Putative sRNA candidate	
SQ2092	3437468	3437711	>	==> Putative antisense to CDS : ntpI(<)	Putative sRNA candidate	
SQ2098	3443918	3444158	>	==> Putative antisense to CDS : CD2965(<)	Putative sRNA candidate	
SQ2107	3470961	3471196	>	==> Putative antisense to CDS : CD2987(<)	Putative sRNA candidate	
SQ2121	3492333	3492093	<	==> Putative antisense to CDS : CD3006(>)	Putative sRNA candidate	
SQ2125	3502479	3502713	>	==> Putative antisense to CDS : CD3014(<)	Putative sRNA candidate	
SQ2146	3524476	3524245	<	==> Putative antisense to CDS : CD3034(>)	Putative sRNA candidate	
SQ2191	3586354	3586585	>	==> Putative antisense to CDS : hrsA(<)	Putative sRNA candidate	
SQ2225	3648007	3648236	>	==> Putative antisense to CDS : CD3131(<)	Putative sRNA candidate	
SQ2235	3655594	3655822	>	==> Putative antisense to CDS : bglA3(<)	Putative sRNA candidate	
SQ2254	3681728	3681957	>	==> Putative antisense to CDS : CD3152(<)	Putative sRNA candidate	
SQ2275	3709710	3709945	>	==> Putative antisense to CDS : gapB(<)	Putative sRNA candidate	
SQ2277	3712370	3712605	>	==> Putative antisense to CDS : glnF(<)	Putative sRNA candidate	
SQ2279	3718957	3719188	>	==> Putative antisense to CDS : pbuX(<)	Putative sRNA candidate	
SQ2339	3801556	3801789	>	==> Putative antisense to CDS : prdC(<)	Putative sRNA candidate	
SQ2355	3818621	3818850	>	==> Putative antisense to CDS : phoT(<)	Putative sRNA candidate	
SQ2358	3830129	3830356	>	==> Putative antisense to CDS : CD3270(<)	Putative sRNA candidate	
SQ2360	3833297	3833059	<	==> Putative antisense to CDS : feoB3(>)	Putative sRNA candidate	
SQ2372	3855880	3856108	>	==> Putative antisense to CDS : CD3293(<)	Putative sRNA candidate	
SQ2376	3864874	3865113	>	==> Putative antisense to CDS : lon(<)	Putative sRNA candidate	
SQ2418	3913836	3914073	>	==> Putative antisense to CDS : bclA3(<)	Putative sRNA candidate	
SQ2424	3924033	3924265	>	==> Putative antisense to CDS : CD3360(<)	Putative sRNA candidate	
SQ2437	3945013	3945246	>	==> Putative antisense to CDS : mgtB(<)	Putative sRNA candidate	
SQ2454	3977993	3978228	>	==> Putative antisense to CDS : CD3399(<)	Putative sRNA candidate	
SQ2478	4042029	4042256	>	==> Putative antisense to CDS : agaS(<)	Putative sRNA candidate	
SQ2507	4088027	4088270	>	==> Putative antisense to CDS : spoIIIF(<)	Putative sRNA candidate	
SQ2512	4096231	4096473	>	==> Putative antisense to CDS : CD3505(<)	Putative sRNA candidate	
SQ2521	4108500	4108734	>	==> Putative antisense to CDS : gcaD(<)	Putative sRNA candidate	
SQ2526	4112003	4111772	<	==> Putative antisense to CDS : murC(>)	Putative sRNA candidate	
SQ2533	4116868	4117095	>	==> Putative antisense to CDS : ksgA(<)	Putative sRNA candidate	
SQ2542	4149429	4149659	>	==> Putative antisense to rRNA : 23s_rRNA(<)	Putative sRNA candidate	
SQ2543	4149593	4149829	>	==> Putative antisense to rRNA : 23s_rRNA(<)	Putative sRNA candidate	
SQ2547	4160300	4160534	>	==> Putative antisense to CDS : ftsH2(<)	Putative sRNA candidate	
SQ2557	4171726	4171969	>	==> Putative antisense to CDS : CD3570A(<)	Putative sRNA candidate	
