# Supplementary material for: Genome-Wide Identification of Regulatory RNAs in the Human Pathogen Clostridium difficile
Source: PLoS Genet. 2013 May 9;9(5):e1003493. doi: 10.1371/journal.pgen.1003493 (PMC3649979; doi:10.1371/journal.pgen.1003493)
Supplement: Table S2 — Summary of sequenced 51-bp reads from NGS of C. difficile. (PDF) [file pgen.1003493.s007.pdf]

Table S2. Summary of sequenced 51-bp reads from NGS of *C. difficile*

	**RNA-seq**		**TAP+**		**TAP-**	
	**Number of reads**	**% of reads**	**Number of reads**	**% of reads**	**Number of reads**	**% of reads**
**Total***	37,139,449	100%	75,557,518	100%	38,125,969	100%
**Filter****	33,596,273	90%	70,790,096	93%	35,002,829	91%
**Remaining*****	3,543,176	10%	4,767,422	6.3%	3,123,140	8.2%
**Genome coverage******	**42x**		**57x**		**37x**	

NGS, next generation sequencing. For 5'-end RNA-seq analysis: TAP+, RNA treated with Tobacco acid pyrophosphatase, TAP-, RNA not treated with Tobacco acid pyrophosphatase. ***All reads after removal of adapter derived sequences; ** reads corresponding to rRNA sequences; *** reads after removal of rRNA sequences; ****** genome coverage for 51–bp reads corresponding to 4,290,252-bp *C. difficile* 630 genome.
