# Supplementary material for: Genome-Wide Identification of Regulatory RNAs in the Human Pathogen Clostridium difficile
Source: PLoS Genet. 2013 May 9;9(5):e1003493. doi: 10.1371/journal.pgen.1003493 (PMC3649979; doi:10.1371/journal.pgen.1003493)
Supplement: Table S3 — Complete list of sRNA detected by deep sequencing. (PDF) [file pgen.1003493.s008.pdf]

Table S3. Complete list of sRNA detected by deep sequencing

Name	Start	End	Strand	Position of TSS	Score	Annotation	Validation	Prediction by Chen et al.
***In silico* predicted sRNAs**								
SQ1002	1760892	1761128	-1	1760987	1.83	ncRNA IGR	NB, RT-PCR	
SQ1005	1762914	1763156	1	1762832	4.2	ncRNA IGR		sCD1763
SQ1021	1786569	1786832	-1	1786848	1	ncRNA IGR		
SQ1033	1801913	1802177	1	1801808	1.22	ncRNA IGR		sCD1802
SQ1038	1803950	1804191	1	1803812	1.46	ncRNA IGR		
SQ1051	1821341	1821592	1	1821308	1	ncRNA IGR		
SQ1076	1851335	1851585	1	1851258	3.42	Antisense 3'UTR	RT-PCR	
SQ1241	2080388	2080629	-1	2080621	2.94	Antisense 3'UTR		
SQ1296	2186776	2187027	-1	2187037	1.79	ncRNA IGR		
SQ1316	2209500	2209732	-1	2209631	4	Antisense CDS		
SQ1339	2257622	2257886	-1	2257754	3.92	ncRNA IGR		
SQ1369	2294722	2294983	-1	2295200		ncRNA IGR		
SQ1376	2300065	2300297	-1	2300340	1.85	Antisense 3'UTR		
SQ1445	2353894	2354130	-1	2354252	1	ncRNA IGR		
SQ1498	2441771	2442021	-1	2441927	1.51	ncRNA IGR	NB, RT-PCR	sCD2442
SQ1517	2471694	2471963	1	2471726		ncRNA IGR	RT-PCR	sCD2472
SQ1549	2503233	2503480	-1	2503927	1	Antisense CDS		
SQ1628	2659760	2660000	-1	2659830	1.06	Antisense CDS		
SQ1633	2661074	2661323	-1	2661358	4	Antisense 3'UTR		
SQ1641	2665093	2665330	1	2665201		Antisense CDS		
SQ1642	2665366	2665614	1	2665201	2.08	ncRNA IGR		
SQ1648	2669774	2670013	-1	2670017	1	ncRNA IGR		sCD2670
SQ1653	2670994	2671248	-1	2671359	4	Antisense 5'UTR		
SQ1656	2671694	2671929	1	2671800	6	ncRNA IGR		
SQ173	308709	308944	1	308776	1.41	ncRNA IGR, Cdi1_3	NB, RT-PCR	sCD309.1
SQ1730	2832724	2832982	-1	2832868	3.76	ncRNA IGR		
SQ178	318125	318367	-1	318363	4	Antisense CDS		
SQ1781	2908031	2908258	1	2908008	4	Antisense CDS	NB, RT-PCR	
SQ1828	2996569	2996813	-1	2996709	3.65	ncRNA IGR		
SQ1870	3073799	3074038	1	3073441	3.05	ncRNA IGR		
SQ190	340758	341018	1	340774	4	ncRNA IGR		sCD341
SQ1900	3119249	3119501	-1	3119058	6.55	ncRNA IGR		
SQ1905	3127018	3127271	-1	3127455	4	ncRNA IGR		
SQ1981	3244936	3245177	-1	3245146	1.95	Antisense 3'UTR		
SQ1999	3266839	3267087	1	3266478	1	ncRNA IGR		sCD3268
SQ2150	3525645	3525884	-1	3525953	4	ncRNA IGR		
SQ2155	3528754	3529000	-1	3528949	4	ncRNA IGR		sCD3529
SQ2179	3562943	3563188	-1	3563322	1.25	Antisense 3'UTR		
SQ22	34059	34298	-1	34576	4	Antisense 5'UTR		
SQ2224	3636785	3637027	-1	3637231	1.17	ncRNA IGR		sCD3637
SQ2233	3654956	3655194	1	3654997	3.76	ncRNA IGR		sCD3656
SQ2303	3766769	3767027	-1	3767006	1.26	ncRNA IGR		sCD3767
SQ2397	3893938	3894182	-1	3894176	4	ncRNA IGR		
SQ2424	3924033	3924265	1	3924016	4	Antisense CDS		
SQ2429	3936132	3936367	1	3936016	1	ncRNA IGR		
SQ2485	4048514	4048762	-1	4048661	1	ncRNA IGR		sCD4049
SQ2503	4079425	4079680	-1	4079622	2.07	ncRNA IGR	NB, RT-PCR	sCD4080
SQ2533	4116868	4117095	1	4116656	4	Antisense CDS		
SQ327	578736	578986	1	578661	4	ncRNA IGR		
SQ367	654653	654911	1	654671	1.14	ncRNA IGR	NB, RT-PCR	sCD655
SQ403	750956	751203	1	750947	4	ncRNA IGR		
SQ408	765236	765478	1	765252	1	ncRNA IGR		
SQ431	812408	812638	-1	812574	1.25	ncRNA IGR		
SQ437	832423	832662	1	832368	4	ncRNA IGR		
SQ476	902987	903237	1	902874	4	Antisense CDS		
SQ495	939000	939241	-1	939080	4	Antisense CDS		
SQ523	1000001	1000248	-1	1000152	4	ncRNA IGR	RT-PCR	
SQ583	1097672	1097907	-1	1097733		Antisense 3'UTR		
SQ587	1109520	1109755	-1	1109583	4	Antisense 3'UTR		
SQ623	1144264	1144513	1	1144307	1	ncRNA IGR		
SQ666	1237859	1238114	-1	1238323		ncRNA IGR		
SQ670	1246231	1246479	-1	1246596	3.76	ncRNA IGR		sCD1247
SQ695	1287192	1287443	-1	1287695	4	ncRNA IGR		sCD1288
SQ726	1322459	1322697	-1	1323156	1.21	ncRNA IGR		
SQ733	1332120	1332352	-1	1332463	2.01	Antisense 5'UTR		
SQ808	1434082	1434312	-1	1434155	1.76	Antisense 3'UTR		
SQ814	1441963	1442204	1	1442085	3.92	ncRNA IGR		
SQ915	1622454	1622699	1	1622607	3.84	ncRNA IGR		sCD1623
SQ995	1749408	1749672	1	1749167	3.57	ncRNA IGR		sCD1750
**New sRNAs detected by deep sequencing**								
CD630_n00010	171529	171631	-1	171631	4	Antisense CDS		
CD630_n00020	182480	182582	-1	182582	4	Antisense CDS		
CD630_n00030 (RCd2)	240878	241078	-1	241078	1.19	Antisense CDS	NB, RT-PCR	
CD630_n00040	280449	280676	1	280449	1.33	ncRNA IGR	NB, RT-PCR	
CD630_n00050	293177	293279	-1	293279	3.76	Antisense CDS		
CD630_n00060	303604	303706	-1	303706	4	Antisense CDS		
CD630_n00070	309860	309962	-1	309962	4	Antisense CDS		
CD630_n00080	312822	312924	-1	312924	4	Antisense CDS		
CD630_n00090	313159	313261	-1	313261	2.96	Antisense CDS		
CD630_n00100	317845	317947	-1	317947	3.84	Antisense CDS		
CD630_n00110	330446	330548	-1	330548	4	Antisense CDS		
CD630_n00120	332212	332314	-1	332314	4	Antisense CDS		
CD630_n00130	335081	335183	-1	335183	4	Antisense CDS		
CD630_n00140	436599	436699	1	436599	4	Antisense CDS		
CD630_n00150	523347	523449	-1	523449	4	Antisense CDS		
CD630_n00160	525168	525270	-1	525270	4	Antisense CDS		

CD630_n00170 (RCd6)	560200	560340	-1	560340	1.24	ncRNA IGR	NB, RT-PCR	
CD630_n00180	577165	577265	1	577165	4	ncRNA IGR		
CD630_n00190	606995	607095	1	606995	1.59	ncRNA IGR		sCD607
CD630_n00200	612749	612840	1	612749	1.1	Antisense CDS		sCD613
CD630_n00210 (RCd4)	655072	655670	1	655072	2.94	ncRNA IGR	RT-PCR	
CD630_n00220	729947	730047	1	729947	2.28	ncRNA IGR		sCD730
CD630_n00230	737885	737985	1	737885	4	ncRNA IGR		
CD630_n00240	811900	812020	1	811900	4	ncRNA IGR		
CD630_n00250	830399	830499	1	830399	4	ncRNA IGR		
CD630_n00260	863008	863108	1	863008	3.19	ncRNA IGR		
CD630_n00270	875830	875990	1	875830	1.9	ncRNA IGR		
CD630_n00280	916647	916747	1	916647	20	ncRNA IGR		
CD630_n00290	920230	920366	-1	920366	1.25	ncRNA IGR		sCD921
CD630_n00300	929839	929939	1	929839	4	Antisense CDS		
CD630_n00310	943984	944084	-1	944084	4	Antisense CDS		
CD630_n00320	963842	963942	1	963842	1.99	Antisense CDS		
CD630_n00330	1052640	1052823	-1	1052823	1.28	ncRNA IGR	NB	sCD1053.1
CD630_n00340	1081635	1081787	-1	1081787	1.53	Antisense CDS	NB	sCD1082
CD630_n00350	1089477	1089730	1	1089477	1.23	ncRNA IGR		
CD630_n00360	1093542	1093640	1	1093542	1.67	Antisense CDS		
CD630_n00370	1124239	1124339	-1	1124339	30.38	Antisense CDS		
CD630_n00380	1124818	1125820	1	1124818	1	ncRNA IGR		
CD630_n00390	1142270	1142666	-1	1142666	3.44	Antisense CDS	NB	
CD630_n00400	1210746	1210962	1	1210746	3	ncRNA IGR		
CD630_n00410 (RCd3)	1259900	1260092	1	1259900	1.02	ncRNA IGR	NB, RT-PCR	
CD630_n00420	1284373	1284971	1	1284373	2.16	Antisense CDS	NB	
CD630_n00430	1284418	1284518	-1	1284518	2.52	Antisense CDS		
CD630_n00440	1322923	1323162	1	1322923	3.84	ncRNA IGR		sCD1323
CD630_n00450	1355629	1355729	-1	1355729	1.69	Antisense CDS		
CD630_n00460	1434587	1435639	1	1434587	1.01	ncRNA IGR		
CD630_n00470	1508308	1508439	-1	1508439	1.6	ncRNA IGR		
CD630_n00480	1513942	1514042	-1	1514042	4	Antisense CDS		
CD630_n00490	1574651	1574751	1	1574651	1	Antisense CDS		
CD630_n00500	1644858	1644958	-1	1644958	4	Antisense CDS		
CD630_n00510	1645024	1646018	1	1645024	2	ncRNA IGR		
CD630_n00520	1688358	1688458	1	1688358	2.02	Antisense CDS		
CD630_n00530	1710541	1710641	-1	1710641	3.92	Antisense CDS		
CD630_n00540	1729907	1730007	-1	1730007	4	Antisense CDS		
CD630_n00550	1746534	1746634	-1	1746634	4	Antisense CDS		
CD630_n00560	1756109	1756833	1	1756109	17.23	ncRNA IGR		
CD630_n00570	1906200	1906300	1	1906200	4	Antisense CDS		
CD630_n00580	1906245	1906345	-1	1906345	2.1	Antisense CDS		
CD630_n00590	1915872	1915972	-1	1915972	2.42	ncRNA IGR		
CD630_n00600	1935311	1936495	1	1935311	1	ncRNA IGR		
CD630_n00610	1937370	1937470	-1	1937470	1	Antisense CDS		
CD630_n00620	1943181	1943450	1	1943181	2.03	ncRNA IGR	NB	
CD630_n00630	2001190	2001290	1	2001190	1.44	Antisense CDS		
CD630_n00640	2180693	2181055	1	2180693	4	ncRNA IGR		
CD630_n00650	2198177	2198277	-1	2198277	1.16	Antisense CDS		
CD630_n00660 (RCd1)	2199358	2199543	1	2199358	3	ncRNA IGR	NB, RT-PCR	sCD2200
CD630_n00670	2228012	2228062	-1	2228062	2.68	Antisense CDS	NB	
CD630_n00680 (RCd5)	2285913	2286311	1	2285913	14.43	ncRNA IGR	NB, RT-PCR	
CD630_n00690	2298609	2298940	-1	2298940	1.92	ncRNA IGR		
CD630_n00700	2340778	2340878	1	2340778	4	Antisense CDS		
CD630_n00710	2353662	2353762	-1	2353762	1.77	Antisense CDS		
CD630_n00720	2450669	2450999	-1	2450999	1.18	ncRNA IGR	NB, RT-PCR	
CD630_n00730	2460535	2460635	1	2460535	4	Antisense CDS		sCD2461
CD630_n00740	2462334	2462434	-1	2462434	4	Antisense CDS		
CD630_n00750	2575450	2575550	1	2575450	1.6	Antisense CDS		
CD630_n00760	2625112	2625715	-1	2625715	2.07	ncRNA IGR		sCD2626
CD630_n00770	2641882	2641982	1	2641882	1.79	ncRNA IGR		
CD630_n00780	2658008	2658108	1	2658008	4	Antisense CDS		sCD2658
CD630_n00790	2662916	2664038	-1	2664038	1.21	ncRNA IGR		
CD630_n00800	2668800	2668935	1	2668800	1.06	ncRNA IGR		
CD630_n00810	2676302	2676402	-1	2676402	2.49	Antisense CDS		
CD630_n00820	2680182	2680319	-1	2680319	4.14	Antisense CDS	NB	
CD630_n00830	2720238	2720338	1	2720238	1.54	Antisense CDS		
CD630_n00840	2861552	2861680	-1	2861680	1.92	ncRNA IGR	NB, RT-PCR	sCD2862
CD630_n00850	2907218	2907622	1	2907218	4	Antisense CDS	NB, RT-PCR	
CD630_n00860	2907464	2908058	-1	2908058	4.58	ncRNA IGR	NB, RT-PCR	
CD630_n00870	2922509	2922609	-1	2922609	1.54	Antisense CDS		
CD630_n00880	2938802	2938902	-1	2938902	1.44	Antisense CDS		
CD630_n00890	2966178	2966343	1	2966178	2.78	Antisense CDS		
CD630_n00900	3111912	3112012	-1	3112012	2.05	Antisense CDS		
CD630_n00910	3137370	3137456	-1	3137456	9.81	Antisense CDS	NB	
CD630_n00920	3172077	3172177	1	3172077	1.12	Antisense CDS		
CD630_n00930	3196622	3196979	-1	3196979	1	ncRNA IGR	RT-PCR	
CD630_n00940	3207485	3207585	-1	3207585	2.8	Antisense CDS		
CD630_n00950	3327549	3327757	-1	3327757	1.98	Antisense CDS		
CD630_n00960	3339986	3340086	1	3339986	1.72	Antisense CDS		
CD630_n00970	3376903	3377003	1	3376903	1.6	Antisense CDS		
CD630_n00980	3379972	3380375	1	3379972	4	Antisense CDS		
CD630_n00990	3396821	3397912	-1	3397912	4	ncRNA IGR	NB	
CD630_n01000	3398302	3398600	1	3398302	30.38	Antisense CDS	NB	
CD630_n01010	3455483	3456904	-1	3456904	1.37	ncRNA IGR		
CD630_n01020	3483594	3483694	1	3483594	4	Antisense CDS		
CD630_n01030	3527684	3527784	-1	3527784	1.52	ncRNA IGR		
CD630_n01040	3587825	3587925	1	3587825	3.92	Antisense CDS		
CD630_n01050	3688590	3688771	-1	3688771	4	ncRNA IGR		
CD630_n01060	3731584	3731705	1	3731584	1	Antisense CDS		

CD630_n01070	3739268	3739368	-1	3739368	3.2	Antisense CDS			
CD630_n01080	3752985	3753119	-1	3753119	1.61	Antisense CDS			
CD630_n01090	3826608	3826694	-1	3826694	1.04	ncRNA IGR			sCD3827
CD630_n01100	3937782	3938212	-1	3938212	3.26	ncRNA IGR			sCD3938.1
CD630_n01110	4081910	4082308	1	4081910	1.26	Antisense CDS			
CD630_n01120	4106130	4106469	-1	4106469	1.01	ncRNA IGR		NB, RT-PCR	sCD4107
CD630_n01130	4217515	4217615	1	4217515	4	Antisense CDS			
**Riboswitch/antitermination/T-box/ribozyme**						**Rfam search**			
CD630_s0010	17634	17855	1	17593	2.51	T-box (Ser)			
CD630_s0030	73019	73242	1	72926	3.49	T-box (Pro)			
CD630_s0050	174147	174250	1	174122	4	SAM			
CD630_s0060	196732	196849	1	196709	2.95	FMN			
CD630_s0160	683173	683427	1	683150	4.97	T-box (Thr)			
CD630_s0170	727944	728413	1	727944	1.42	Group I intron			
CD630_s0190	917371	917598	1	917345	6.59	T-box			
CD630_s0200	990470	990939	1	990470	2.96	Group I intron			
CD630_s0210	1152624	1152886	1	1152580	3.46	T-box (Leu)			
CD630_s0220	1364065	1364171	1	1364048	1.18	SAM			
CD630_s0230	1488489	1488742	1	1488461	2.4	T-box (Ala)			
CD630_s0260	1736753	1737222	1	1736753	2.96	Group I intron			
CD630_s0290	1818880	1819348	1	1818880	4	Group I intron			
CD630_s0300	1830948	1831204	1	1829848	11.25	T-box; T-box			
CD630_s0330	1917499	1917604	-1	1917608	1.65	SAM			
CD630_s0340	1918846	1919022	1	1918828	4.05	Lysine			
CD630_s0350	1930135	1930604	1	1930135	1.34	Group I intron			
CD630_s0360	1973231	1973352	-1	1973366	4	FMN			
CD630_s0380	1992919	1993388	1	1992919	2.96	Group I intron			
CD630_s0390	2055462	2055740	1	2055427	1.51	T-box			
CD630_s0400	2066659	2066883	1	2066622	11.84	T-box (Arg)			
CD630_s0410	2102880	2103349	1	2102880	1.27	Group I intron			
CD630_s0420	2110692	2110794	1	2110675	1.9	SAM			
CD630_s0430	2285202	2285301	-1	2285309	6.26	TPP			
CD630_s0440	2294385	2294663	-1	2294701	39.94	T-box (Trp)			
CD630_s0450	2347170	2347400	-1	2347427	5.02	T-box (Arg)			
CD630_s0460	2368975	2369143	-1	2369166	5.09	Lysine			
CD630_s0470	2436110	2436208	-1	2436221	2.83	Purine			
CD630_s0480	2451328	2451572	1	2451297	21.22	T-box (Thr)			
CD630_s0490	2598418	2598668	-1	2598701	16.38	T-box (Asn)			
CD630_s0500	2694683	2694785	-1	2694785	3.92	Purine			
CD630_s0510	2886067	2886295	-1	2886324	2.87	T-box (Arg)			
CD630_s0530	2954861	2955331	-1	2955331	2.96	Group I intron			
CD630_s0540	3017752	3018007	-1	3018036	1.41	T-box (Trp)			
CD630_s0550	3027704	3027953	-1	3027981	1.28	T-box (Ile)			
CD630_s0560	3114793	3115065	-1	3115126	5.5	T-box (Asn)			
CD630_s0570	3148001	3148211	-1	3148258	1.07	RNase P			
CD630_s0590	3607770	3607949	-1	3608396	1.83	Lysine			
CD630_s0600	3687389	3687738	-1	3687890	1.63	tmRNA			
CD630_s0610	3773053	3773227	1	3773030	5.76	Lysine			
CD630_s0620/Cdi2_1	3800624	3801050	-1	3801140	10.03	c-di-GMP-II		RT-PCR	
CD630_s0640	3861645	3861760	1	3861632	4	FMN			
CD630_s0660	4136360	4136623	-1	4136660	4	T-box (Met)			
CDmisc_RNA_16	2725664	2725774	-1	2725857	9.41	Glycine			
CDmisc_RNA_17	2640424	2640508	1	2640394	1.28	Glycine			
CDmisc_RNA_5	578842	578948	1	578824	1.28	ykkC-yxkD			
CDmisc_RNA_7	2891492	2891604	-1	2891615	4.51	0 (yybP-ykoY)			
CD630_01200	163160	163327	1	163369	1.93		0		
CD630_03240	391011	391203	1	391363	1.39	Cobalamin			
CD630_14890	1724139	1724245	1	1724103	1.75	SAM			
CD630_15470	1795496	1795799	1	1795496	8.64		0		
CD630_32560	3813925	3814267	-1	3814267	8.9	T-box			
CD630_33590	3921928	3922366	1	3921928	3.13		0		
CD630_33681	3935442	3935864	-1	3935864	5.52		0		
CD630_34350	3861645	3861760	-1	4027273	1.56		0		
CD630_31170	3631070	3631206	-1	3631206	1.95		0		
CD630_35980	4206264	4206459	-1	4206459	1.58		0		
Cdi1_1	2296352	2296523	-1	2296523	4	GEMM RNA motif		NB, RT-PCR	
Cdi1_2	3266536	3266886	-1	3266886	2.63	GEMM RNA motif		RT-PCR	
Cdi1_3	308776	309272	1	308776	1.41	GEMM RNA motif		NB, RT-PCR	
Cdi1_4	3379972	3380472	1	3379972	4	GEMM RNA motif		RT-PCR	
Cdi1_5	1142166	1142666	-1	1142666	3.44	GEMM RNA motif		RT-PCR	
Cdi1_6	2285913	2286352	1	2285913	14.43	GEMM RNA motif		NB, RT-PCR	
Cdi1_7	2907218	2907318	1	2907218	4	GEMM RNA motif		NB, RT-PCR	
Cdi1_8	2297483	2297816	1	2297483	6	GEMM RNA motif		NB, RT-PCR	
Cdi1_9	2671800	2671951	1	2671800	6	GEMM RNA motif		NB, RT-PCR	
Cdi1_10	1653767	1653925	-1	1653925	6	GEMM RNA motif		NB, RT-PCR	
Cdi1_11	3936231	3936389	1	3936231	6	GEMM RNA motif		NB, RT-PCR	
Cdi1_12	3303255	3303464	-1	3303464	3.06	GEMM RNA motif		NB, RT-PCR	
Cdi2_3	3306644	3306893	-1	3306816		c-di-GMP-II		RT-PCR	
Cdi2_2	3826608	3826694	-1	3826694	1.04	c-di-GMP-II		RT-PCR	
Cdi2_4	4105635	4105873	-1	4105873	2.9	c-di-GMP-II		NB, RT-PCR	
