# Supplementary material for: Genome-Wide Identification of Regulatory RNAs in the Human Pathogen Clostridium difficile
Source: PLoS Genet. 2013 May 9;9(5):e1003493. doi: 10.1371/journal.pgen.1003493 (PMC3649979; doi:10.1371/journal.pgen.1003493)
Supplement: Table S4 — sRNA extremity identification by 5′RACE and 5′/3′RACE. (PDF) [file pgen.1003493.s009.pdf]

Table S4. sRNA extremity identification by 5'RACE and 5'3'RACE

Name	5'end RACE position	5'-end RNA-seq 5' position	Strand	3'end 5'3'RACE position
SQ173	308770	308776	+	
SQ1002	1761105	1761105	-	1760914
	1761106	1760987		1760904
		1761212		
SQ1498	2441928	2441927	-	
	2441933			
SQ2025	3306816		-	
	3306807	cleavage 3306807		
	3306797			
	3306788			
CD630_n00030	241079	241078	-	240905
(RCd2)	241065			240730
	241067			
CD630_n00170	560340	560340	-	560195
(RCd6)	560320			560196
				560197
CD630_n00210	655066	655119	+	
(RCd4)	655075	655072		
CD630_n00680	2285913	2285913	+	2286288
(RCd5)				
