# Supplementary material for: Genome-Wide Identification of Regulatory RNAs in the Human Pathogen Clostridium difficile
Source: PLoS Genet. 2013 May 9;9(5):e1003493. doi: 10.1371/journal.pgen.1003493 (PMC3649979; doi:10.1371/journal.pgen.1003493)
Supplement: Table S5 — Conservation of regulatory RNAs in bacterial strains. (PDF) [file pgen.1003493.s010.pdf]

Table S5. Conservation of regulatory RNAs in bacterial strains

Species	Strains	sRNAs	%	Riboswitches	%
C. difficile	R20291	172	81%	57	79%
C. difficile	CD196	170	80%	57	79%
C. difficile	QCD-23m63	151	71%	51	71%
C. difficile	QCD-32g58	167	79%	41	57%
C. difficile	QCD-37x79	172	81%	54	75%
C. difficile	QCD-63q42	179	84%	52	72%
C. difficile	QCD-66c26	170	80%	54	75%
C. difficile	QCD-76w55	170	80%	52	72%
C. difficile	QCD-97b34	170	80%	49	68%
C. difficile	NAP07	145	68%	50	69%
C. difficile	NAP08	146	69%	50	69%
C. difficile	VPI	183	86%	51	71%
C. difficile	CD37*	174	82%	-	-
C. acetobutylicum	ATCC824	1	0.5%	4	5.6%
C. beijerinckii	NCIMB8052	4	1.9%	2	2.8%
C. botulinum	ATCC3502	3	1.4%	3	4.2%
C. cellulolyticum	ATCC35319	1	0.5%	0	0.0%
C. cellulovorans	743B	2	0.9%	4	5.6%
C. kluyveri	DSM555	1	0.5%	4	5.6%
C. ljungdahlii	ATCC49587	2	0.9%	4	5.6%
C. novyi	NT	1	0.5%	5	6.9%
C. perfringens	ATCC13124	5	2.4%	1	1.4%
C. phytofermentans	ISDg	2	0.9%	0	0.0%
C. saccharolyticum	WM1	1	0.5%	1	1.4%
C. sticklandii	DSM519	2	0.9%	4	5.6%
C. tetani	E88	4	1.9%	4	5.6%
C. thermocellum	ATCC27405	1	0.5%	1	1.4%
B. subtilis	168	0	0.0%	3	4.0%
M. tuberculosis	H37Rv	0	0.0%	0	0.0%
E. coli	K12	0	0.0%	0	0.0%
H. pilori	P12	0	0.0%	0	0.0%
E. faecalis	V583	0	0.0%	0	0.0%

**Genome without annotation*
