# Supplementary material for: Genome-Wide Identification of Regulatory RNAs in the Human Pathogen Clostridium difficile
Source: PLoS Genet. 2013 May 9;9(5):e1003493. doi: 10.1371/journal.pgen.1003493 (PMC3649979; doi:10.1371/journal.pgen.1003493)
Supplement: Table S6 — Strains and plasmids used in this study. (PDF) [file pgen.1003493.s011.pdf]

Table S6. Strains and plasmids used in this study

Strain	Genotype	Origin
E. coli		
TOP 10	F- *mcrA* Δ(*mrr-hsdRMS-mcrBC*) f80*lacZ*DM15 Δ*lacX*74 *deoR, recA1 araD139* Δ(*ara-leu*)7697 *galK rpsL(StrR) endA1 nupG*	Invitrogen
HB101 (RP4)	*supE44 aa14 galK2 lacY1* Δ(*gpt-proA*) 62 *rpsL20 (*Str^R*)xyl-5 mtl-1 recA13* Δ(*mcrC-mrr*) *hsdS*$_B$ (r_B-m_B-) RP4 (Tra^+ IncP Ap^R Km^R Tc^R)	Laboratory stock
C. difficile		
630	Sequenced reference strain	Laboratory stock
630Δ*erm*	630 Δ*ermB*	Laboratory stock (Hussain et al. 2005)
R20291	PCR-ribotype 027 hypervirulent strain	Laboratory stock
CDIP51 (630/p)	630 Δ*erm* strain carrying pRPF185 vector	pRPF185→630Δ*erm*
CDIP93 (630/pSQ1498)	630 Δ*erm* strain carrying pDIA5981	pDIA5981→630Δ*erm*
CDIP94 (630/pAS-SQ1498)	630 Δ*erm* strain carrying pDIA5983	pDIA5983→630Δ*erm*
CDIP291 (630/pRCd6)	630 Δ*erm* strain carrying pDIA6178	pDIA6178→630Δ*erm*
CDIP205 (630/pAS-RCd6)	630 Δ*erm* strain carrying pDIA6109	pDIA6109→630Δ*erm*
CDIP289 (630/pRCd2)	630 Δ*erm* strain carrying pDIA6174	pDIA6174→630Δ*erm*
CDIP207 (630/pAS-RCd2)	630 Δ*erm* strain carrying pDIA6111	pDIA6111→630Δ*erm*
CDIP96 (630/p*dccA*)	630 Δ*erm* strain carrying pDIA5987	pDIA5987→630Δ*erm*
CDIP219 (630/p)	630 Δ*erm* strain carrying pRPF185Δ*gusA* vector	pDIA6103→630Δ*erm*
CDIP274 (630/p*CD0183*)	630 Δ*erm* strain carrying pDIA6168	pDIA6168→630Δ*erm*
**Plasmid**		
pRPF185	P_{tet}-*gusA* Tm^R expression and cloning vector	(Fagan and Fairweather 2011)
pDIA6103	pRPF185Δ*gusA* vector derivative	This work
pDIA5981	pRFF185 derivative carrying P_{tet}-SQ1498 for inducible SQ1498 expression	This work
pDIA5983	pRFF185 derivative carrying P_{tet}-AS_SQ1498 for inducible antisense RNA expression to SQ1498	This work
pDIA6178	pRFF185 derivative carrying P_{tet}-CD630_n00170 (RCd6) for inducible RCd6 expression	This work
pDIA6109	pRFF185 derivative carrying P_{tet}-AS_ RCd6 for inducible antisense RNA expression to RCd6	This work

pDIA6174	pRFF185 derivative carrying P_{tet}-CD630_n00030 (RCd2) for inducible RCd2 expression	This work
pDIA6111	pRFF185 derivative carrying P_{tet}-AS_ CD630_n00030 (AS-RCd2) for inducible antisense RNA expression to RCd2	This work
pDIA5987	pRFF185 derivative carrying P_{tet}-CD1420 for inducible *dccA* (*CD1420)* expression	This work
pDIA6168	pRFF185 derivative carrying P_{tet}-CD0183 for inducible *CD0183* expression	This work

Fagan RP, Fairweather NF. 2011. *Clostridium difficile* has two parallel and essential Sec secretion systems. *J Biol Chem* **286**(31): 27483-27493.

Hussain HA, Roberts AP, Mullany P. 2005. Generation of an erythromycin-sensitive derivative of *Clostridium difficile* strain 630 (630Delta*erm*) and demonstration that the conjugative transposon Tn916DeltaE enters the genome of this strain at multiple sites. *J Med Microbiol* **54**(Pt 2): 137-141.
