# Supplementary material for: Genome-Wide Identification of Regulatory RNAs in the Human Pathogen Clostridium difficile
Source: PLoS Genet. 2013 May 9;9(5):e1003493. doi: 10.1371/journal.pgen.1003493 (PMC3649979; doi:10.1371/journal.pgen.1003493)
Supplement: Table S7 — C-di-GMP riboswitches. (PDF) [file pgen.1003493.s012.pdf]

Table S7. C-di-GMP riboswitches

Name	*In silico* prediction	Adjacent gene	Strand	5'_start	Score	Promoter	3'_end	Size, nt	Northern blot, nt	CDIP51/ CDIP96 ratio*
**c-di-GMP I**										
Cdi1_1		*CD1990* chp	-	2296523	4	-10 TATTAT	2296135	388	600	0.07
Cdi1_2	SQ1999	*CD2797* adhesion protein	-	3266886	2.63		3266579	307		2.2
Cdi1_3	SQ173	*CD0245 flgB*	+	308776	1.41	σ^A TATACT 17 bp TTTAAA	309167	391	160	2.4
Cdi1_4		*CD2889* phage protein	+	3379972	4	σ^A TATTAT 17 bp TTGAAC	3380375	403		2.1
Cdi1_5		*CD0977.1* phage protein	-	1142666	3.44	σ^A TATAAC 16 bp TTCTAT	1142270	396	280, 350	2.1
Cdi1_6 RCd5		*CD1981* transcriptional regulator	+	2285913	14.43	σ^A TATTAT 17 bp TTGAAA	2286311	398		10.1
Cdi1_7		*CD2517.1* chp	+	2907218	4	σ^A TATTAT 17 bp TTGAAT	2907622	404		4.02
Cdi1_8		*CD1990.3* chp	+	2297483	6	σ^A TATTAT 17 bp TTGAAT	2297888	405		5.8
Cdi1_9	SQ1656	*CD2309* chp	+	2671800	6	σ^A TATTAT 17 bp TTGAAT	2672197	397	140, 400	6.8
Cdi1_10		*CD1424* chp	-	1653925	6	σ^A TATTAT 17 bp TTGAAC	1653521	404		6.8
Cdi1_11		*CD3368.2* chp	+	3936231	6	σ^A TATTAT 17 bp TTGAAT	3936635	404		6.8
Cdi1_12	SQ2429	*CD2830* chp	-	3303464	3.06	σ^A TATAAT 17 bp TAGACA	3303075	389	170, 145	4.2
**c-di-GMP II**										
Cdi2_1 CD630_s0620		*CD3246* putative surface protein	-	3801406	10.03	σ^A TATAAT 17 bp TAGACA	3801064	342		0.1 (Lee, Baker et al. 2010; Chen, Sudarsan et al. 2011)
Cdi2_2 CD630_n01090		*CD3267* response regulator	-	3826694	1.04	-10 TTTAAT	3826608	86		0.2
Cdi2_3	SQ2025	*CD2831* collagen-binding protein precursor	-	3306816		σ^A TATAAT 17 bp TAGACA	3306682	134	105, 145, 160, 200	0.4
				4105873	2.9	-10 TTTAAT	4105797	77		
Cdi2_4		*CD3513* pilin	-	4105967		σ^A TATATA 17 bp TTTCAA		170	100, 160, 200	0.08

The “Cdi” are numbered as previously described for c-di-GMP I and for c-di-GMP II riboswitches (Sudarsan, Lee et al. 2008; Lee, Baker et al. 2010). Corresponding “SQ” names from our *in silico* prediction are also indicated. The position of 5’ start was identified by 5’-end RNA-seq analysis with indicated score for comparison between TAP treated and TAP non-treated sample. The position of 3’ end is from *in silico* analysis (Sudarsan, Lee et al. 2008; Lee, Baker et al. 2010). The presence of -10 and -35 boxes for σ^A-dependent promoters is indicated. The size of the transcripts detected in Northern blot is indicated. “chp” conserved hypothetical protein. * The CDIP51/CDIP96 (630/p / 630/p*dccA*) fold change was determined by qRT-PCR analysis.

Chen, A. G., N. Sudarsan, et al. (2011). "Mechanism for gene control by a natural allosteric group I ribozyme." RNA 17(11): 1967-1972.

Lee, E. R., J. L. Baker, et al. (2010). "An allosteric self-splicing ribozyme triggered by a bacterial second messenger." Science 329(5993): 845-848.

Sudarsan, N., E. R. Lee, et al. (2008). "Riboswitches in eubacteria sense the second messenger cyclic di-GMP." Science 321(5887): 411-413.
