# Supplementary material for: Genome-Wide Identification of Regulatory RNAs in the Human Pathogen Clostridium difficile
Source: PLoS Genet. 2013 May 9;9(5):e1003493. doi: 10.1371/journal.pgen.1003493 (PMC3649979; doi:10.1371/journal.pgen.1003493)
Supplement: Table S8 — CRISPR arrays. (PDF) [file pgen.1003493.s013.pdf]

Table S8. CRISPR arrays

Name	Spacer number	*cas* genes	Strand	5'_start	Score	Promoter	3'_end	Size, nt	Rfam search
**CD630_n00380*** CRISPR 3/4	6+3	0	+	1124818	1		1125820	1002	CRISPR-DR14
CD630_n00400 CRISPR 5	1	0	+	1210746	3		1210962	216	0
**CD630_n00460** CRISPR 6	13	0	+	1434587	1.01	-10 TATATT 17 bp TTGGTA	1435639	1052	CRISPR-DR14
**CD630_n00510** CRISPR 7	12	0	+	1645024	2	-10 TATAAT	1646018	994	CRISPR-DR14
CD630_n00560* CRISPR 8	8	0	+	1756109	17.23	-10 TATAAT	1756833	724	CRISPR-DR14
CD630_n00600* CRISPR 9	15	0	+	1935311	1	-10 TATAAT	1936495	1184	CRISPR-DR14
CD630_n00690 CRISPR 10	2	0	-	2298940	1.92	-10 TATAAT	2298609	331	CRISPR-DR14
**CD630_n00790*** CRISPR 11	14	0	-	2664038	1.21	-10 TATAAT	2662916	1122	CRISPR-DR14
CD630_n00860 CRISPR 12	6	*CD2451-CD2455*	-	2908058	4.58	σ^A TATAAT 16 bp TTCAAA	2907464	594	CRISPR-DR14
**CD630_n00990*** CRISPR 15/16	3+6	0	-	3397912	4		3396821	1091	CRISPR-DR14
CD630_n01010 CRISPR 17	19	*CD2975-CD2982*	-	3456904	1.37	σ^A TAAAAT 16 bp TTGCAG	3455483	1421	CRISPR-DR14

The numbering for CRISPR RNA is from CRISPRdb database (Grissa, Vergnaud et al. 2007)(http://crispr.u-psud.fr/crispr/). CRISPR 5 corresponds to a questionable structure predicted in rRNA region and carrying only one spacer separating two divergent repeat sequences. The names of CRISPR RNA within prophage regions are shown in bold. "*" highly expressed CRISPR RNA. The position of 5' start was identified by 5-end RNA-seq analysis with indicated score for comparison between TAP treated and TAP non-treated sample. The position of 3' end was identified by *in silico* analysis (http://crispr.u-psud.fr/crispr/). The presence of -10 and -35 boxes for σ^A-dependent promoters is shown. RNA sequences were analysed for matches in Rfam database http://rfam.sanger.ac.uk/, "0", no matches.

Grissa, I., G. Vergnaud, et al. (2007). "The CRISPRdb database and tools to display CRISPRs and to generate dictionaries of spacers and repeats." BMC Bioinformatics 8: 172.
