# Supplementary material for: Genome-Wide Identification of Regulatory RNAs in the Human Pathogen Clostridium difficile
Source: PLoS Genet. 2013 May 9;9(5):e1003493. doi: 10.1371/journal.pgen.1003493 (PMC3649979; doi:10.1371/journal.pgen.1003493)
Supplement: Table S9 — Oligonucleotides used in this study. (PDF) [file pgen.1003493.s014.pdf]

Table S9. Oligonucleotides used in this study.

Name	Sequence (5'-3')	Description
**pRPF185 cloning**		
IMV507	GGGATTTCTCACATAAAATAGAG	5’pRPF185
IMV508	TAAAATAAGCTTGATCGTAGCG	3’pRPF185
IMV530	CCCGAGCTCTTTTGGAGAAATTAATATGTTTA	5'CD1420-SacI
IMV531	GGGGATCCACAATGTTAATAATCATTTTTATCA	3'CD1420-BamHI
LS89	CCCGAGCTCAGAAGCTTAAAACCATAAAATACACCA	5’SQ1498-SacI
LS88	GGGGATCCTATTTATTTTTCCCAAAGTACATA	3’SQ1498-BamHI
LS85	GGGGATCCAGAAGCTTAAAACCATAAAATACACCA	5'-AS-SQ1498-BamHI
LS86	CCCGAGCTCGATGACTTATATGGAGGATGTAAGGA	3'AS-SQ1498-SacI
OS505	CCCCGAGCTCACATAGAAGATAGTAAAACTAGCT	5'CD630_n00170-SacI
OS506	CGGGATCCCAAATTGTTGAAATGATGAA	3'CD630_n00170-BamHI
OS507	CGGGATCCACATAGAAGATAGTAAAACTAGCT	5'AS-CD630_n00170-BamHI
OS508	CCCCGAGCTCCCCAAATTGTTGAAATGATGAA	3'AS-CD630_n00170-SacI
OS521	CCCCGAGCTCAATAAAATAAATAAATTAGCTTATCCA	5'CD630_n00030-SacI
OS522	CGGGATCCGTATATTAATGATAGTTGCTATTGAT	3'CD630_n00030-BamHI
OS523	CGGGATCCAATAAAATAAATAAATTAGCTTATCCA	5'AS-CD630_n00030-BamHI
OS524	CCCCGAGCTCGTATATTAATGATAGTTGCTATTGAT	3'AS-CD630_n00030-SacI
OS616	GAAGGCCTAAATAATGTATATTTAGGTGAC	5'-CD183-StuI
OS617	GGGGATCCGTATTTATCAATTTGTCATATTAC	3'-CD183-BamHI
OS618	CCTCGAGCTCATTTTAATTATACTCTATCA	5'-halfStuI-SacI-Ptet
OS620	CCTTCTCGAGAGGATCCATAAATATAAAATAAAATAGAGGCT	3'-halfStuI-XhoI-BamHI-ter
OS621	CGATGCCCTGGACTTCATGAA	5'-Ptet-pRPF185
**Northern blot/ Quantitative RT-PCR**		
OS353	GCCAGTTGCCAAAAAGAGTC	5'SQ173
OS354	CAAAATGACTGCCAAAAAGG	3'SQ173
OS359	GAAGGCTTTTGCTTTCTGACTT	5’SQ1002
OS360	GGCAAGATGCCTATCATACAAA	3'SQ1002
OS400	AATATTAAATGGAAGAAAGAAATTTCAAG	5’SQ1985
OS401	TCAGCTTCTTTTTCTGTGTGAGA	3'SQ1985
OS363	TGAAGTATATTCTATTATTGGGCACCT	5'SQ2025
OS364	TTATATTCATAGCCGGTTGCAC	3'SQ2025
OS365	CAAATGTAATTGAACATATGGAGTGA	5’SQ367
OS366	GGGTTTAATACTTTTACTTTGACTCG	3’SQ367
OS367	TGTTGAAAAGTTAGAGAGTACAATCAAG	5’SQ931
OS368	AAGTGCTTTGTTGACAGAGTTCA	3’SQ931
OS369	AGAAGCTTAAAACCATAAAATACACCA	5’SQ1498
OS370	GATGACTTATATGGAGGATGTAAGGA	3’SQ1498
OS379	GTTAGATTTGAGGTTGGAAAAGG	5’CD630_n00660
OS380	GGCGTATCCTGCATCTCTATT	3’CD630_n00660
OS385	GTTGCACCACTAACTCAATATGC	5’Cdi2_4
OS386	ATAAAATAGAAACGTTGATTTATGTTCTG	3’Cdi2_4

OS421	GGGAGACCGTGTCTGGTAAA	5'CD630_n00410
OS422	GAAACACCGGTGGCTAAGTAA	3'CD630_n00410
OS435	ACTTATATATTCCACCTCTACCATATAAAGACTCTAC	3'spacer1-CRISPR16
OS446	TTTGTACCATTCGAGGTTAAAGTG	5'CD630_n00030
OS447	AAAAAATATACGCCCTATAAAAGCG	3'CD630_n00030
OS460	GTGGCAACTCTCCCTCACAT	5'CD630_n00170
OS461	CCCAAATTGTTGAAATGATGAA	3'CD630_n00170
OS466	GTATATTTAGGTGACATATTGGATAAGCTAA	3'CD630_n00030
OS519	AAGATGGCGAAGGTTGGTTA	5'CD0183
OS520	TTCCTTTTTCCAGCTCTTCAA	3'CD0183
OS557	TAGAATAAAACTAAATCGGCAAAACT	5'Cdi1_9
OS558	CTTTTTGGCAACTGGCTGA	3'Cdi1_9
OS572	GTAAACCATGCCAGCCAGTT	5'Cdi1_12
OS573	TTTGAGTTAGGCCGGGAGTA	3'Cdi1_12
**Quantitative RT-PCR**		
OS271	GCAAACAATCAAATGGGAAGA	5'CD0239
OS272	ATATTGCAAGTCCAGCAGCA	3'CD0239
OS377	GCATATTGGGAATAATGAAAAAGG	5'CD630_n00210
OS378	TGATTACTCTTTTAACCTACCCCCTA	3'CD630_n00210
OS419	GTCAGCCAGTTGCCAAAGA	5'Cdi1_6
OS420	AATTGCAGTAATCGCGTGAA	3'Cdi1_6
OS431	TGACGCAAAGCTATAGGGACT	5'Cdi1_7
OS432	CTCTTTGGCAACTGGCTGAC	3'Cdi1_7
OS452	CAGCCAGTTGCCAAAAAGAT	5'Cdi1_5
OS453	CATCTTTATTATATCGCATTTTGAGAA	3'Cdi1_5
OS513	TGATGCTATGCCTAAAATAGAAGAAA	5'CD0245
OS514	CCATTTGCAAAACTTATCAAAGC	3'CD0245
OS528	TGACCCATTAACTGGAGCAT	5'CD1420
OS529	CTTGTCTCCTACATTATGACCTTCA	3'CD1420
OS553	CATGAAAAAGGCAATATCTTGTG	5'Cdi1_2
OS554	TTCAACTGCCCTTAGCTTCC	3'Cdi1_2
OS561	CAGCCAGTTGCCAAAAAGAT	5'Cdi1_1
OS562	CATCTTAAGCCCTCCTCTTGC	3'Cdi1_1
OS565	AACTAAATCGGCAAAACTAGAGAAA	5'Cdi1_8
OS566	CTCTTTGGCAACTGGCTGAC	3'Cdi1_8
QRTBD37	GGGAGACTTGAGTGCAGGAG	5'16S
QRTBD38	GTGCCTCAGCGTCAGTTACA	3'16S
**5' and 5'3' RACE**		
OS404	AGTCCCTATAGCTTTGCGTCA	SQ173
OS405	TTTGTATGATAGGCATCTTGCC	SQ1002
OS406	AAGTCAGAAAGCAAAAGCCTTC	SQ1002
OS407	CCCAACTTTGTATGATAGGCATC	SQ1002
OS408	GCAAAAGCCTTCTGACTCTATG	SQ1002
OS411	AGGTGCCCAATAATAGAATATACTTCA	SQ2025
OS412	GTGCAACCGGCTATGAATATAA	SQ2025
OS413	CCTTTTTCATTATTCCCAATATGC	CD630_n00210
OS414	TAGGGGGTAGGTTAAAAGAGTAATCA	CD630_n00210
OS503	GTCCCTATAGCTTTGCGTCACTA	CD630_n00680
OS504	AGGGGGATATATGATATATGTTTATGG	CD630_n00680
OS509	CTATTTGTATAGGGTATTATATTATGT	CD630_n00170
OS510	CCTCAATTGTTGCCACTATT	CD630_n00170
LS98	TGGTGTATTTTATGGTTTTAAGCTTCT	SQ1498
LS99	GTCATCATTATAGTAACCCAACAAT	SQ1498
